# Supplementary material for: Adhesion differentials control the rheology of biomimetic emulsions
Source: Biophys J. 2026 Mar 5;125(7):1686–700. doi: 10.1016/j.bpj.2026.03.004 (PMC13351756; doi:10.1016/j.bpj.2026.03.004)
Supplement: Document S1. Figures S1–S15 [file mmc1.pdf]

Biophysical Journal, Volume *125*

Supplemental information

Adhesion differentials control the rheology of biomimetic emulsions

Quentin Guigue, Marc Besse, Raphael Voituriez, Alexis M. Prevost, Elie Wandersman, Matthias Merkel, and Lea-Laetitia Pontani

Supplementary information:

Adhesion differentials control the rheology of biomimetic emulsions

Quentin Guigue, Marc Besse, Raphael Voituriez, Alexis M. Prevost, Elie Wandersman, Matthias Merkel†, Lea-Laetitia Pontani†

† Corresponding authors:

lea-laetitia.pontani@sorbonne-universite.fr
matthias.merkel@univ-amu.fr

This PDF file includes:

- Supplementary text
- Figures S1 to S15
- SI References

Contents

1 DNA sequences **3**

2 Experimental set-up **3**

3 Straight channel geometry **4**

4 Image analysis **4**

5 P14 sequences **6**

6 Red/Green droplet proportion across conditions and unmixing **6**

7 Quasi-staticity **7**

8 Droplet shape and shear decomposition **7**

8.1 Triangle-based quantities . . . 7
8.2 Locally averaged quantities . . . 8
8.3 Quantification of droplet shape variation . . . 9
8.4 Fitting of Q_* from experimental f_r curves . . . 9
8.5 Beyond a Q_{proj}-dependent f_r . . . 9

9 Quantification of the flow velocity for the prediction of $Q_{xx}(x)$ **11**

10 Packing fraction evolution **12**

11 Estimation of interface tension ratio from contact angles at triple junctions **12**

12 Estimation of the parameter α **16**

13 A gradient in packing fraction suggests pumping **16**

13.1 Hypothesis . . . 16
13.2 Flow velocity measurements . . . 16

14 Supplementary Videos **18**

14.1 Movie S1: flow of an heterogeneous emulsion . . . 18
14.2 Movie S2: flow of an heterogeneous emulsion with fluorescent tracers in the continuous phase . . . 18

1 DNA sequences

The DNA sequences presented below share the same structure : a common backbone spacer sequence of 49 bp and a specific sticky end of various length (underlined part in the sequences) separated by a single non-hybridizable base, serving as a flexible junction. A biotin group for grafting of the sequence onto the lipids is ligated to the 3' end of the sequences through the intermediary of a triethylene glycol spacer. Sequences are named after the number of base pairs constitutive of the sticky end, varying between 0 bp and 14 bp.

A complementary sequence (CS) to the backbone is hybridized to these sequences before their grafting onto the droplets. This stiffens the spacer to the sticky and favors adhesion [3, 2].

Palindromic DNA sequences

P0: 5'-G CAT TAC TTT CCG TCC CGA GAG ACC TAA CTG ACA CGC TTC CCA TCG CTA[BtnTg]-3'

P6: 5'- <u>TAC GTA</u> A G CAT TAC TTT CCG TCC CGA GAG ACC TAA CTG ACA CGC TTC CCA TCG CTA[BtnTg]-3'

P10: 5'-<u>AAT CAT GAT T</u> A G CAT TAC TTT CCG TCC CGA GAG ACC TAA CTG ACA CGC TTC CCA TCG CTA[BtnTg]-3'

P14: 5'-<u>TAT GCA TAT GCA TA</u> A G CAT TAC TTT CCG TCC CGA GAG ACC TAA CTG ACA CGC TTC CCA TCG CTA[BtnTg]-3'

Complementary sequence

CS: 5'-TAG CGA TGG GAA GCG TGT CAG TTA GGT CTC TCG GGA CGG AAA GTA ATG C-3'

2 Experimental set-up

Once the emulsion is prepared, the emulsion mix is left to cream, in a tube with pressure control, and in a water bath at ≈ 40°C to avoid any preliminary interaction between the droplets. The emulsion is then injected into the passivated microchannel through a Tygon connected to a pressure pump (MFCS-8C Fluigent), at a typical pressure of ∼20 mBar. We let the emulsion flow in the channel until a high packing fraction is reached (note that imposed pressure can be modulated to help the filling and packing processes). The outlet and drain of the channel are both connected to a waste collection Eppendorf.

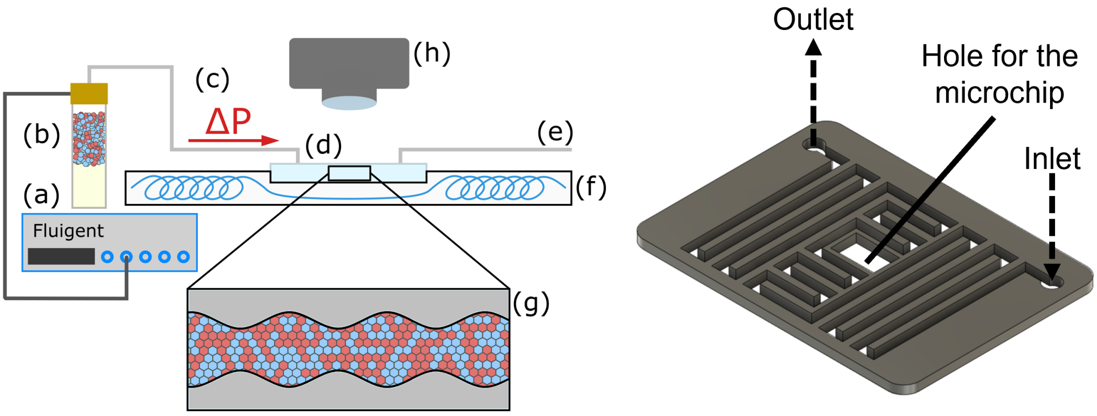

Figure 1: (Left) Microfluidic setup. A pressure controller (a) is connected to a reservoir containing the creamed emulsion (b). We impose a pressure (c) to push the emulsion from the reservoir to the microchip (d) set on a cooling stage (f). The emulsion flow in the microchip undergoes oscillatory shear due to the design of the chip (g). Waste is collected at the outlet of the chip (e). Images are acquired using spinning disk confocal microscopy (h). (Right) Schematic of the custom cooling stage.

Due to the sensibility of DNA hybridization to temperature, the microfluidic chip is mounted on a custom cooling microscope stage. It consists of three PMMA plates assembled together with optical glue. The middle plate contains channels, which allow us to circulate cold water through the stage. All three plates of the stage have a 15×25 mm rectangular hole in the middle to accommodate for the micro-chip and its tubing during the experiment. The custom stage is connected to a CC-K6 Cooling bath thermostat (Huber). Cold water is therefore circulated into the microscope stage which cools down the micro-chip by conduction. This allows us to maintain the chip temperature at ≈ 18°C, ensuring proper adhesion of all DNA sequences.

3 Straight channel geometry

The design of the straight channel is overall similar to the undulated one, except that the oscillations were removed. In order to find the equivalent position between the two channels, we added some marks along the straight channel that are separated by 420µm, the periodicity of the undulated channel.

Note that the constriction at the exit of the channel is still present to help for the packing of the droplets. As this constriction inherently applies a mechanical perturbation on the droplets, no acquisition was proceeded in this position.

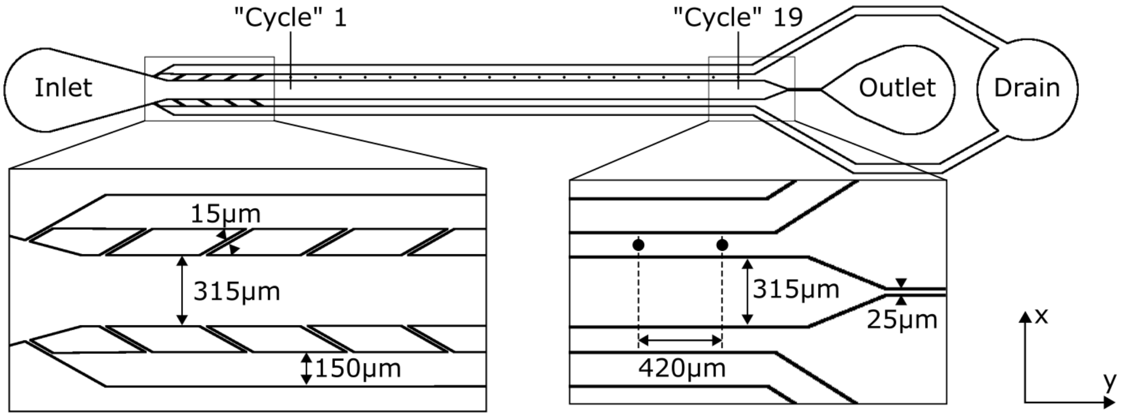

Figure 2: Schematic and dimensions of the straight channel used for control experiments.

4 Image analysis

Fluorescence channels are initially analyzed separately. In Figure 3A, we show the two superimposed channels of fluorescence for better visualization. A typical static experiment corresponds to 4 to 10 acquisitions of the twenty positions of the channel (nineteen in the case of the straight channel; an example of a raw image is given in Figure 3A). For each experiment and each fluorescence channel, we therefore construct a sample consisting of 2 images per acquisition of the full channel. This sample is then used to classify pixels between foreground (droplets contour) and background using a Random Forest Algorithm with Ilastik [1] parameterized with the following features: Color/Intensity 3.5-5-10 σ, Edge 3.5-5 σ, Texture 3.5-5 σ. The algorithm is manually trained until the classifier is able to correctly predict the contour of the droplets. A manual check is performed for each image in order to limit detection errors. An example of the resulting segmentation is shown in Figure 3B. A custom Fiji routine is then used to identify the droplets out of the resulting binary images. In particular, we use the Analyze Particles module to detect objects that are larger than 1500 pixels^2 ($\simeq 76\mu m^2$) and with a circularity above 0.70.

Once the process is completed for both channels, the obtained binarized masks are summed to recover a complete picture of the image and a surface Voronoi tessellation is produced using Fiji as shown in Figure 3C. This tessellation will later allow us to select the droplets that will be use or not in the upcoming analysis, identify the neighborhood of each droplet and evaluate their local packing fraction. Next, we use the Python Sci-kit image library to geometrically characterize each droplet. We focus on the droplets whose corresponding Voronoi cell are not touching neither the left/right borders of the image (as they correspond to cropped droplet in between two waves) nor the top/bottom borders (corresponding to outer droplets touching the border of the channel and therefore inherently having a different neighborhood). An example of those selected droplets is shown by yellow colored cells on Figure 3C.

For each remaining droplet we use the regionprops function to measure its position, orientation, area and perimeter. Position and orientation are obtained by fitting the droplets with an ellipse that has the same second moment (resulting orientation and example of fitted ellipse are shown in Figure 3D-E). The area is evaluated by pixel counting and droplets with an area larger than 10^5 pixels^2 $\simeq 5000\,\mu m^2$ are excluded. The perimeter is approached by the length of a line fitting the border pixels using a 4-connectivity, an example of those identified contours is shown in Figure 3F-G.

The area a is then used together with the area of the corresponding Voronoi cell to compute a local packing fraction as $\phi_{\mathrm{loc}} = a/a_v$, a_v being the area of the Voronoi cell. Droplets with a local packing fraction lower than 0.94 are discarded in further quantification.

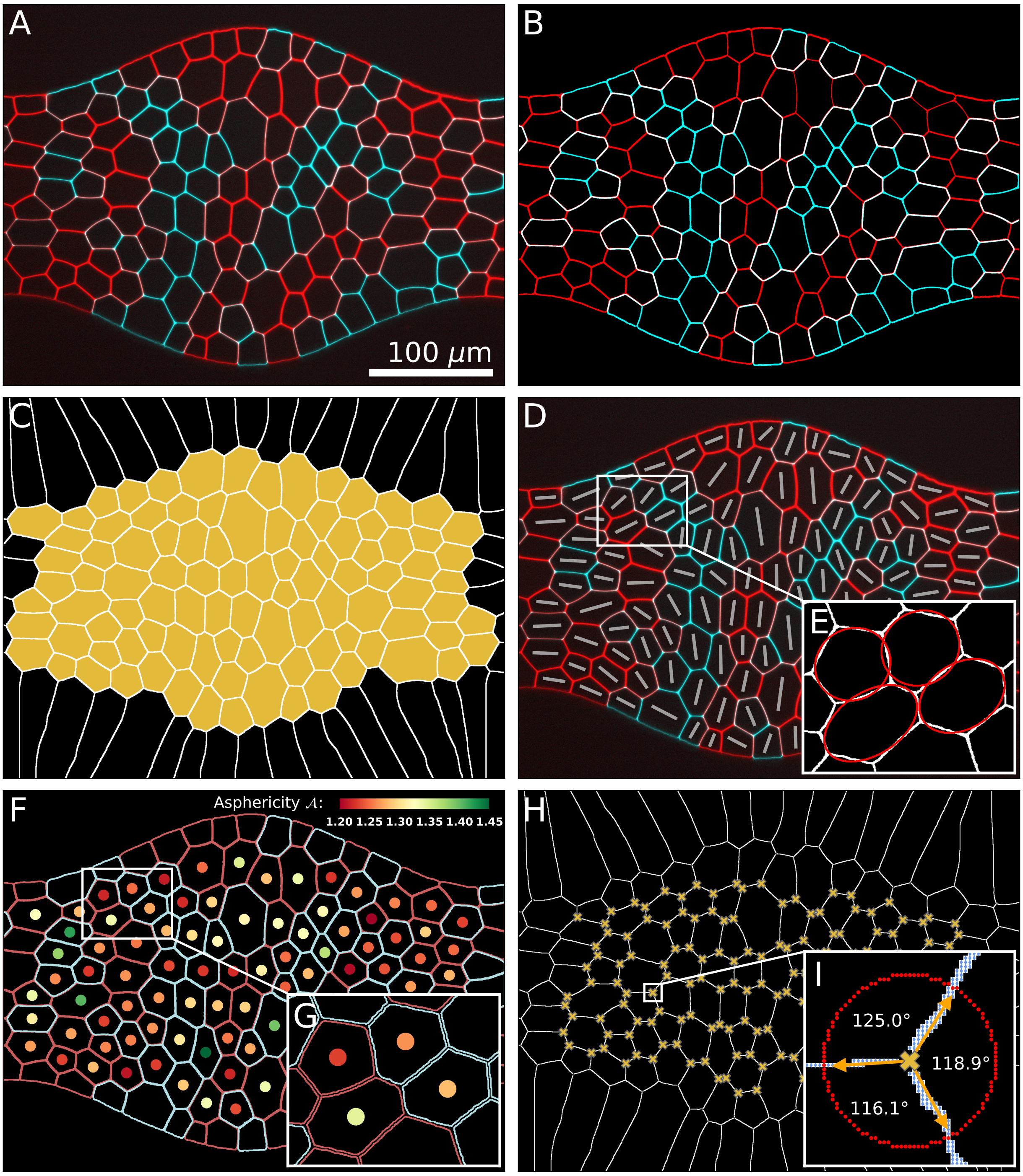

Figure 3: (A) Confocal image of two populations of droplets grafted with palindromic sequences. Red-red droplets interact through 10bp, blue-blue droplets interact through 0bp, i.e. they do not interact through specific adhesion, and similarly, red-blue droplets do not interact. (B) Result of the segmentation after training on Ilastik. (C) A surface Voronoi tessellation is performed in order to exclude droplets on the edge of the channel and select the inner droplets (here highlighted in yellow). (D-E) Droplets are fitted by ellipses (E) in order to extract their position and orientation (grey segments) with respect to the horizontal axis (D). (F-G) The area a of each droplet is approximated through pixel counting while the perimeter p is approximated by the length of a line fitting the pixels of the contour (red and blue lines). Based on this information, we compute the shape factor as $\mathcal{A} = p^2/4\pi a$ (colored dots). (H-I) Edges and vertex of the network can be identified separately. Each inner vertex (yellow cross, vertex constituted exclusively by inner droplets identified in (C)) is associated to its contributing edges from which we identify the point coordinates (blue dots) that we use to fit a line and find the direction vector (orange arrow) to further quantify the pairwise angles between edges around the vertex (I). Inset (I) shows an example of the resulting quantification.

A shape factor index is also computed for each droplet as $\mathcal{A} = p^2/4\pi a$, with p the perimeter of the droplet. This shape factor represents the deviation of a given shape compared to a perfect disk which would yield $\mathcal{A} = 1$, the higher the deformation the higher $\mathcal{A}$ is. Colored dots on Figure 3F-G represent the intensity of the measured shape factor of the associated droplet.

Using the Voronoi tessellation Figure 3C, we are able to separate vertex and edges of the network Figure 3H (yellow crosses and white lines respectively) and keep only inner vertices, i.e. vertices formed exclusively by inner droplets. Constitutive edges of the vertex are identified by drawing a small circle around its center (red dotted circle in Figure 3I). We focused only on cases where the vertex is formed by exactly three droplets. Each vertex is then broken down into pixel-points coordinates (blue dots in Figure 3I) that are fitted by a straight line, allowing us to approximate a direction vector (orange arrows in Figure 3I). Once the three vectors of the vertex are computed, we pair-wisely quantify the angles between the three edges of the vertex and repeat for each vertex of the image.

5 P14 sequences

Similarly to the results presented in Fig. 2A, we imaged emulsions made with P0/P14 (pink crosses) and P6/P14 (purple diamonds) mixtures as they progress inside the channel cycles. Compared to the PO/P10 and P6/P10 experiments, these conditions are thus associated with a higher average binding energy, which in turn leads to a higher deformation at the beginning of the channel (see Figure 4-Left for shape factor and Figure 4-Right for ΔQ_{xx} measurements). In addition to this, the highest adhesion differential, namely P0P14, also leads to a larger increase in deformation across cycles.

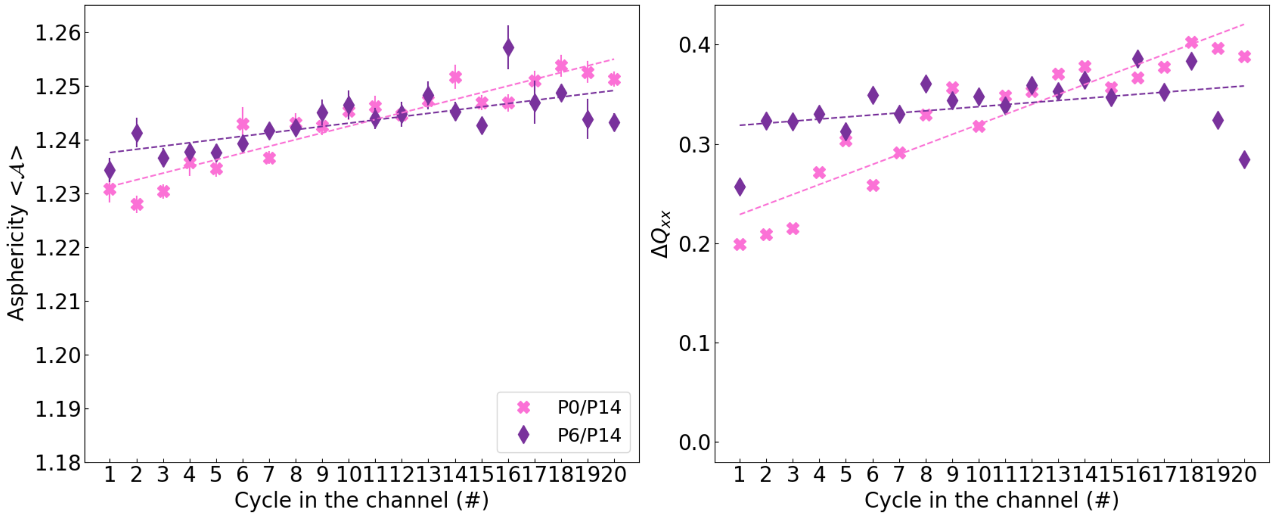

Figure 4: Deformation of droplets in P0/P14 (pink crosses) and P6/P14 (purple diamonds) emulsions. (Left) Average asphericity $\langle \mathcal{A} \rangle$ computed over all the droplets in each undulation cycle for both conditions. The analysis was performed on static images acquired after the flow was arrested in the channel. Error bars represent the standard error of the mean across experimental repetitions. (Right) Evolution of ΔQ_{xx}, calculated as the amplitude of $Q_{xx}(x)$ variations within a channel oscillation, as a function of the undulation.

6 Red/Green droplet proportion across conditions and unmixing

The proportion of droplets from each population remains stable over the successive shear cycles (Figure 5 Left), indicating an absence of emulsion sorting along the channel. A slight deviation from a 1:1 proportion of each population appears only when the most adhesive droplets P14 are involved but still no sorting of the emulsion along the channel is observed in that case. Moreover, these emulsions are not used for most of the characterization and modelling in the main text.

In addition, we quantified the mixing in heterogeneous emulsions as the ratio between the total heterotypic interface length (i.e. red/blue contacts) and the total interface length (red/blue, blue/blue and red/red contacts), along the oscillatory channel's positions (Figure 5 Right). This analysis reveals that the mixing remains stable across shear cycles.

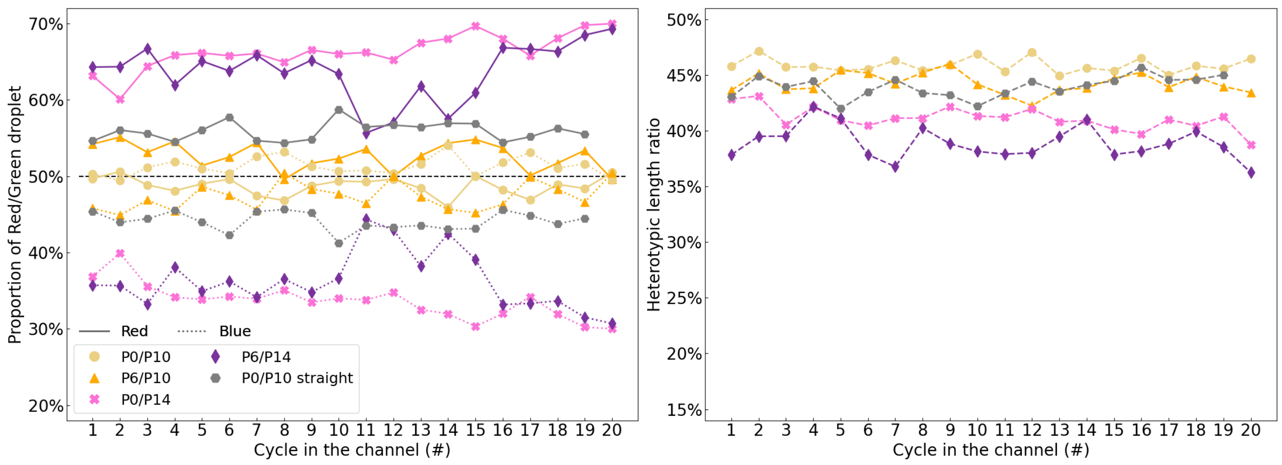

Figure 5: (Left) Proportion of red (solid lines) and blue (doted lines) droplets across channel positions averaged over all heterogeneous emulsions used in static acquisitions. (Right) Heterotypic length ratio, i.e. total heterotypic interface length (red/blue contacts) divided by the total interface length, as a function of the position in the channel averaged over all heterogeneous emulsions used in static acquisitions.

7 Quasi-staticity

T1 transitions and the associated elastic relaxation typically take place on a time scale below the image acquisition time interval of $\Delta t = 5 \times 10^{-2}$ s. This is much faster than the applied deformation: the fastest flow speed of 50 µm/s corresponds to a travel time of 8.4 s for any droplets across a single shear cycle. Therefore, T1 events and elastic relaxation occur on much shorter timescales than the shear timescale, which is defined by the channel geometry.

In addition, we examined how droplet asphericity ($\mathcal{A}$) and anisotropy (Q_{xx}) depend on flow velocity. Figure 6 displays the results for P10 homogeneous emulsions. Both the asphericity (Figure 6 Left) and the droplet anisotropy (Figure 6 Right) are independent of the average flow speed.

8 Droplet shape and shear decomposition

8.1 Triangle-based quantities

We follow Ref. [5] in quantifying triangle and droplet shape. Briefly, in a first step, we triangulate the segmented droplet image. To this end, we take any vertex (or triangular aqueous region). If the three adjacent droplets are fully visible, we define a triangle by connecting the three barycenters of the droplets. If more than three droplets meet at an aqueous region, we proceed as for many-fold vertices in Ref. [5]. Briefly, in this case, we compute the average $\boldsymbol{c}$ of the barycenters of all adjacent droplets, and then define triangles between two adjacent droplet centers and $\boldsymbol{c}$, respectively.

For each triangle n, we then compute a shear rate tensor $\tilde{V}_{ij}^n$ and a shape anisotropy tensor Q_{ij}^n in the following way. Briefly, to compute the shear rate tensor for some time step $t \to t' = t + \delta t$, we consider the affine transformation matrix M_{ij}^n required to transform triangle n at time t into triangle n at time t'. Then, the triangle-based velocity gradient is computed as $V_{ij}^n = (M_{ji}^n - \delta_{ij})\delta t$. Finally, the shear rate tensor $\tilde{V}_{ij}^n$ is computed as the symmetric, traceless part of V_{ij}^n.

To compute the shape tensor Q_{ij}^n of any triangle at any time point t, we proceed again following Ref. [5]. Briefly, we first consider the affine transformation matrix $\boldsymbol{S}^n$ that transforms an equilateral reference triangle into triangle n at time t. The matrix $\boldsymbol{S}^n$ can be expressed as a tensor product:

$$\boldsymbol{S}^n = \left(\frac{a^n}{a_0}\right)^{1/2} \exp(\boldsymbol{Q}^n) \cdot \mathbf{R}(\theta^n). \tag{1}$$

The first term represents an isotropic scaling, where a^n and a_0 are the area of the triangle and the reference triangle, respectively. The second term is a shear transformation parameterized by the triangle shape anisotropy tensor Q_{ij}^n, which is symmetric and traceless. θ^n is an absolute rotation angle of the triangle, where $\mathbf{R}(\theta^n)$ denotes a counter-clockwise rotation by θ^n. Note that the orientation angle of Q_{ij}^n and θ^n two different and completely independent angles. The product decomposition in (1) is unique. In particular, it uniquely defines

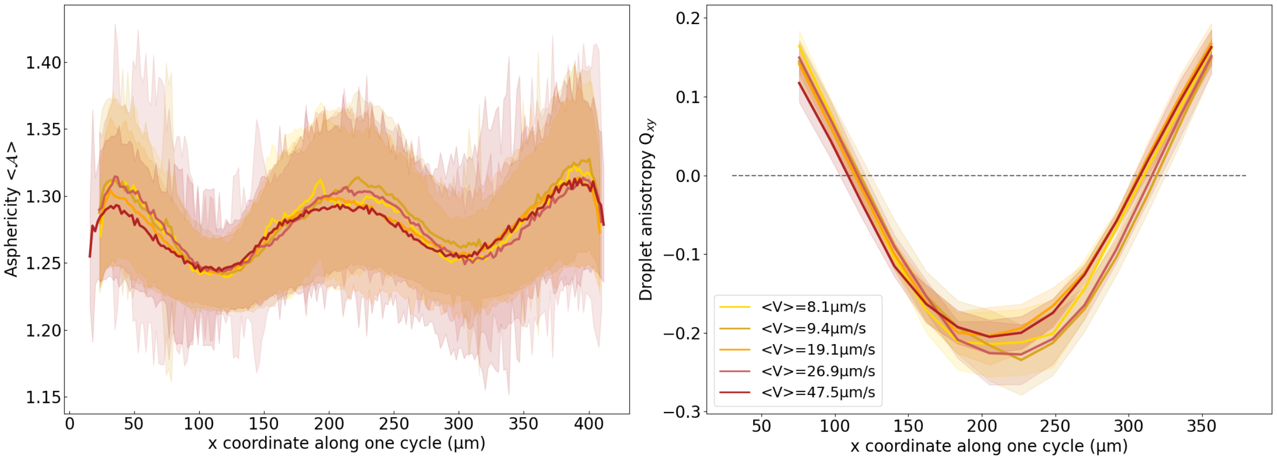

Figure 6: Droplet shape properties over one shear cycle in the channel for different flow velocities, here shown for P10 homogeneous emulsion in the fifth position of the undulated channel. (Left) Shape factor $\mathcal{A} = p^2/4\pi a$ as a function of the position. (Right) Droplet anisotropy Q_{xx} as a function of the position (see section 8). For both $\mathcal{A}$ and Q_{xx}, shaded areas represent the standard deviation. Both plots indicate that droplet shape is independent of flow velocity.

Q^n_{ij} [5]. Furthermore, one can show that its magnitude, $Q = \sqrt{Q^2_{xx} + Q^2_{xy}}$, corresponds to $\log(\mathrm{AR})/2$, where AR is the aspect ratio of the uniquely defined ellipse whose perimeter goes through all three triangle points and whose barycenter coincides with the triangle barycenter [5].

8.2 Locally averaged quantities

To obtain locally averaged quantities for droplet shape anisotropy Q_{xx}, shear rate $\tilde{V}_{xx}$, and the reversible fraction f_r, we first divide our images into a grid of $a^b_0 = 96 \times 96\,\mathrm{pixel}^2$ (i.e. $a^b_0 = 21.5\,\mu\mathrm{m} \times 21.5\,\mu\mathrm{m}$) boxes, which we label here by their center positions $b = (x, y)$. We next compute the intersection of each box b with each triangle n, which respectively results in a polygon of area $a^{n\cap b}$. Clearly, the sum of these polygon areas over all boxes corresponds to the corresponding triangle area $\sum_b a^{n\cap b} = a^n$. Conversely, the sum over all polygon areas over all triangles is $\sum_n a^{n\cap b} = a^b$, which can be smaller than the full box area a^b_0, since some boxes (i.e. those at the at the image boundaries) are not entirely covered by triangles. Yet, in all our quantifications, we only took boxes into account that, at the time point of interest, are entirely covered by triangles, i.e. those boxes for which $a^b = a^b_0$.

Then, the average droplet shape anisotropy for any given box b is given by the area-weighted average of the shape tensors of the covering triangles:

$$Q^b_{ij} = \frac{1}{a^b} \sum_n a^{n\cap b} Q^n_{ij} \tag{2}$$

with $i, j \in \{x, y\}$. Similarly, the average shear rate of a given box b is given by:

$$\tilde{V}^b_{ij} = \frac{1}{a^b} \sum_n a^{n\cap b} \tilde{V}^n_{ij}. \tag{3}$$

To be able to compute the reversible fraction, we also need to quantify the droplet shape derivative $\mathrm{d}\boldsymbol{Q}/\mathrm{d}t = \partial\boldsymbol{Q}/\partial t + \mathrm{v}_i \partial_i \boldsymbol{Q}$ for a given box for some time interval $t \to t' = t + \delta t$. The partial derivative part is computed using (2) at two subsequent time points, and dividing their difference by δt. Yet, we observe that this contribution is negligible as compared to the advective term (stationary state; see Figure 8A). To compute the advective term, we consider for each triangle n at time t a modified version n' at time t'. We therefore remember that n was created by placing its corners onto the barycenters of three droplets. We then construct n' from the barycenters of these droplets *at time* t'. Note that n' is not necessarily in the usual triangulation at n', since the three droplets may not necessarily be neighbors at time t' any more. Then, the advective term for box b is computed as

$$\Delta Q^b_{\mathrm{adv.},ij} = \frac{1}{2a^b_0} \sum_n \left(a^{n\cap b} - a^{n'\cap b}\right)\left(Q^n_{ij} + Q^{n'}_{ij}\right). \tag{4}$$

We further compute a corotational term $\Delta Q^b_{\mathrm{corot.},ij}$ following Ref. [5], which we find to be negligible as well (Figure 8A).

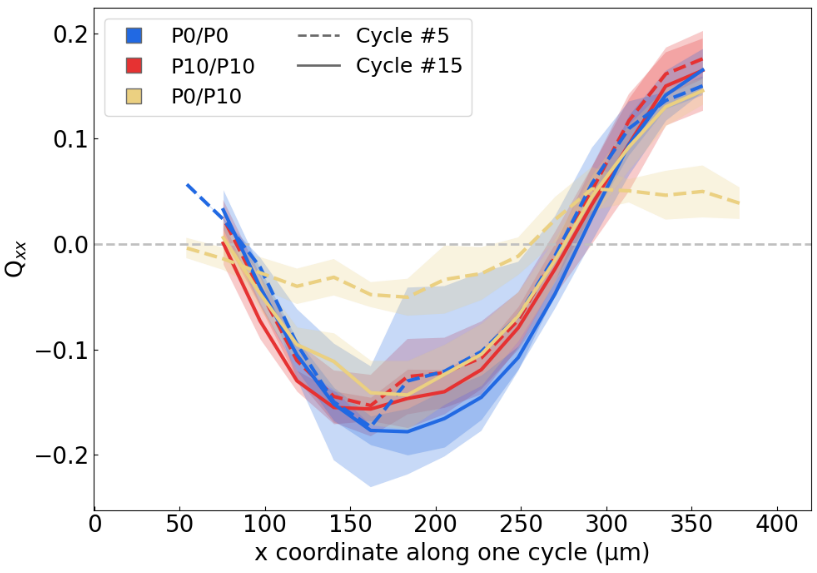

Figure 7: Examples of the Q_{xx} component of droplet shape plotted as a function of the x position in the channel, and averaged over time and across the channel width y. An increase of amplitude between position #5 (dashed lines) and position #15 (solid lines) can be observed for heterogeneous emulsions (P0/P10) (yellow curve), but not for the homogeneous emulsions P10 (red curves) and P0 (blue curves). Shaded areas represent the standard deviation.

In a next step, we average these box-based quantities, Q_{xx}, $\tilde{V}_{xx}$, $\Delta Q^b_{\mathrm{adv.},ij}$, and $\Delta Q^b_{\mathrm{corot.},ij}$, among all boxes with the same x coordinate. For this averaging, we first average over time, where we select only those boxes that are fully covered at least 95% of all time points. This yields a grid of boxes with the time-averaged quantities. At the end, we average over y in order to obtain averages for each column, i.e. quantities that only depend on x. Finally, for each x column, we compute the reversible fraction as:

$$f_r(x) = \frac{\langle Q^b_{xx}(t+\delta t) - Q^b_{xx}(t) + \Delta Q^b_{\mathrm{adv.},xx} + \Delta Q^b_{\mathrm{corot.},xx}\rangle_{t,y}}{\langle \tilde{V}_{xx}\rangle_{t,y}}. \tag{5}$$

Here δt is the length of the time interval between two movie frames and $b = (x, y)$ denotes the box position.

8.3 Quantification of droplet shape variation

To quantify how droplet shape $\boldsymbol{Q}$ changes across a cycle, we fit the measured x-dependent $Q_{xx}(x)$ to a polynomial of 5th degree, and then determine its minimum Q^{min}_{xx} and maximum Q^{max}_{xx}. We then define the amplitude for that cycle $\Delta Q_{xx} := Q^{\mathrm{max}}_{xx} - Q^{\mathrm{min}}_{xx}$. This is shown in Figure 9 (left) for data from a P0/P10 emulsion in a wavy channel. Indeed, the amplitudes in Q_{xx} increase with cycle.

8.4 Fitting of Q_* from experimental f_r curves

In the main text Figure 4D, we plot the 50% droplet anisotropy, Q_*, for given experimental reversible fraction curves $f_r(Q_{\mathrm{proj}})$. These curves are given by pairs of (Q_{proj}, f_r) values, each corresponding to one of the 96 pixel-wide columns described before. To obtain Q_* from these data, we consider values of f_r lying between 0 and 1.2 with their associated Q_{proj} and sort them. We then construct a set of consecutive data points that include the up to two lowest (respectively highest) f_r values larger (respectively smaller) than 0.5. We require to have at least one data points below and one above $f_r = 0.5$, and ignore $f_r(Q_{\mathrm{proj}})$ curves that do not fulfill this criterion. Thus, the size of the set of selected data points varies between 2 and 4. A linear fit is performed across those data points and the Q_* value is computed as the intercept of this linear fit with $f_r = 0.5$.

8.5 Beyond a Q_{proj}-dependent f_r

While we showed that a single dependency of f_r on Q_{proj} can explain most of our experimental observations, we also saw indications of additional effects.

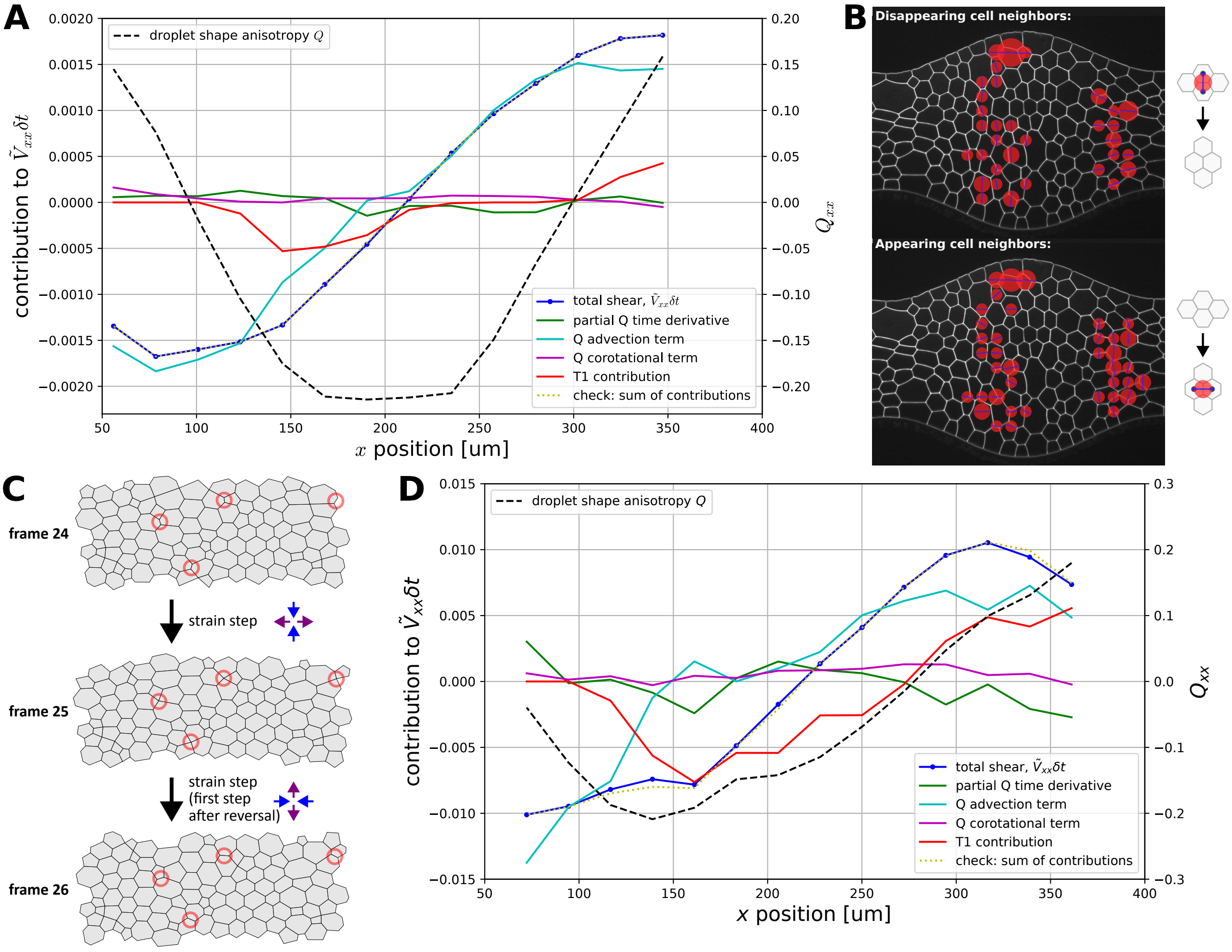

Figure 8: (A) Example of the shear rate decomposition (Eq. (1) in the main text) for a P10 emulsion across the length of a single cycle. All quantities are averaged over time and y. The overall local shear between two subsequent frames, $\tilde{V}_{xx}\delta t$, is shown as blue solid curve. We decompose this into four contributions: shear due to local change in Q_{xx} (partial time derivative, green solid curve), the advective contribution $\Delta Q^b_{\text{adv.},xx}$ (turquoise solid curve), a corotational contribution $\Delta Q^b_{\text{corot.},xx}$ (magenta solid curve), and the contribution due to T1 transitions (red solid curve). The yellow dotted curve is the direct sum of these four contributions; it overlaps with the overall shear curve as a sanity check. The black dashed line shows the droplet shape Q_{xx}. (B) As another sanity check, for the same experiment as in A, we detect individual T1 transitions by either a disappearing cell-cell neighborship (top) or an appearing cell-cell neighborship (bottom). The circle area indicates the number of T1 transitions in that region, and the blue bar indicates the average orientation of the cell-cell connection (scaled such that the bar length equals the circle diameter if all events are aligned). These data are consistent with the red curve in panel A. (C) In our simulations at shear reversals, we find that $f_r > 1$. As illustration, we show vertex model simulation snapshots for 100 cells, a strain amplitude of 0.5, and a T1 cutoff of 0.3. We observe a strong $f_r > 1$ signature in these simulations. This is consistent with T1 transitions observed in these snapshots: With the shear reversal occurring at frame 25, the red circles indicate three places where a T1 fusion occurs from frame 24 to frame 25, which continues by a T1 resolution from frame 25 to frame 26. Notably, the T1 resolution in frames 25-26 does *not* revert the fusion, but continues in the way it has started, thus leading to a T1 transition extending along the horizontal in these snapshots. Meanwhile, while overall the tissue is stretched along the vertical in frames 25-26. As a consequence of the transversal T1 transitions, droplet shape stretches along the vertical more than the tissue overall shear, implying $f_r > 1$. (D) Shear rate decomposition like in panel A, but for a P0 emulsion. Note that around $x = 250\,\mu\text{m}$, T1 transitions occur opposite to the local shear direction. We interpret this as a consequence of non-local interactions between adjacent regions of the emulsions.

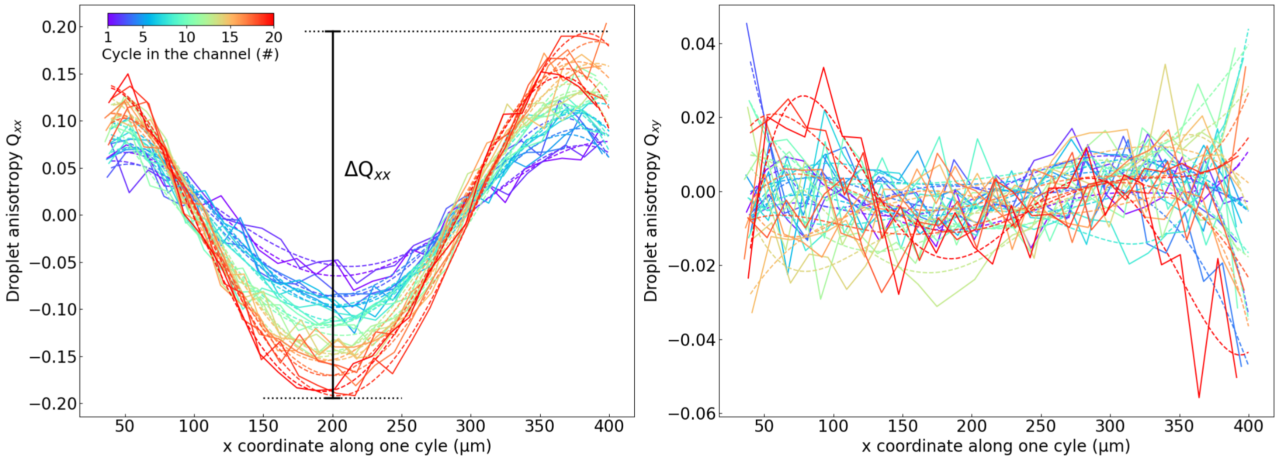

Figure 9: (Left) Spatial variation of the Q_{xx} component of droplet shape, averaged over the channel width for each cycle (color) for the case of a P0/P10 emulsion. Dashed lines represent the fit of the curves by a degree-5 polynomial. (Right) Quantification of the Q_{xy} component of droplet shape in the same experimental condition.

For instance, we saw that both in experiments and simulations, f_r became larger than one for the smallest, negative values of Q_{proj}. In the simulations, we saw that this was related to the switching of the shear direction: In Figure 8C, we show three frames for a vertex model simulation around the switch of the shear direction. We find that in the transition leading up to the switch, frames 24-25, a few edges fuse into manyfold vertices (T1 fusions). Meanwhile, after the switch of shear directions, in frames 25-26, these manyfold vertices revolve, but instead of going back to how they were before, they continue. In other words, the T1 transitions continue to expand horizontally, while tissue overall expands vertically (contracting horizontally). As a consequence, droplet shapes expand vertically, but due to the T1 orientation more than the tissue overall, i.e. $f_r > 1$ for the transition between frames 25-26. This is some form of short-term memory.

In the experiment, the reason for $f_r > 1$ might be the same. Alternatively or additionally, $f_r > 1$ in the experiments may also be created by elastic non-local effects: Whenever there is some local deformation of a given droplet in the emulsion, this will also affect the shapes of close-by droplets. We can observe this for example during T1 transitions, where we see a quasi-instantaneous deformation of surrounding droplets. This deformation will slightly change the shapes of the surrounding droplets, and may trigger another T1 transition. The transitions that we observe at shear reversal in the experiments may in part due to such non-local effects.

We also see indications for such non-local effects in our shear decomposition for some emulsions. In Figure 8D, we show the shear decomposition for a P0 emulsion. Notably, around $x = 250\,\mu\text{m}$, the emulsion is sheared extending along the horizontal (positive values of blue curve), but T1 transitions occur along the vertical (negative values of red curve). This would be impossible if emulsion behavior would be only controlled locally, since shearing horizontally would induce T1 transitions horizontally. Hence, this suggests a role of non-local effects, for instance by the nearby region between $x = 100 \dots 200\,\mu\text{m}$ undergoing vertically extending T1 transitions, whose elastic propagators may push a sufficient number of droplets in the $x = 250\,\mu\text{m}$-region to also undergo vertically extending T1 transitions.

9 Quantification of the flow velocity for the prediction of $Q_{xx}(x)$

In order to predict $Q_{xx}(x)$ for the reversible fraction function, we need the velocity v_x, at least up to a prefactor (see Methods in main text). Because the emulsions are essentially incompressible, the total flux of emulsion should be the same at every position x when integrated across the width of the channel. Using the approximation that v_x only depends on x, but not on y, and denoting the channel width at some position x by $h(x)$, we thus have:

$$\text{v}_x(x) = \frac{h(0)\text{v}_x(0)}{h(x)}. \tag{6}$$

Yet, we found that this yields a slightly imprecise prediction for the shear rate $\tilde{V}_{xx}$ averaged over time and y as described in the previous section. This is due to the fact that the triangulations do not cover the entire channel; there is a region close to the boundary not covered by triangles since the triangles only reach up until the barycenters of the boundary droplets. This boundary region is captured by (6), but it is not captured by $\tilde{V}_{xx}$. Since what is most relevant for the prediction of the Q_{xx} is what happens to the triangular region, we thus

decided to base our Q_{xx} prediction on $\tilde{V}_{xx}$.

To compute the prediction described in the Methods part of the main text, we need a smooth function, while experimentally we measured $\tilde{V}_{xx}$ only averaged over the discrete x columns. We thus fitted the velocity and velocity gradient data to a generic Fourier expansion:

$$\mathrm{v}_x^{\mathrm{fit}}(x) = a_0 + a_1 \cos\left(\frac{2\pi(x - x_1)}{\lambda}\right) + a_2 \cos\left(\frac{4\pi(x - x_2)}{\lambda}\right) \tag{7}$$

Here, $a_{0/1/2}$ are fit parameters corresponding to Fourier mode amplitudes and $x_{1/2}$ are fit parameters corresponding to phase shifts. The constant $\lambda = 420\,\mu\mathrm{m}$ is the wave length of the cycles. In practise, we first fit $\mathrm{dv}_x^{\mathrm{fit}}/\mathrm{d}x$ to the measured $\tilde{V}_{xx}$ over x data (note that $\tilde{V}_{xx} = \partial_x v_x$ because of incompressibility). This allows us to fix $a_{1/2}$ and $x_{1/2}$. Afterwards, we obtain a_0 by fitting $\mathrm{v}_x^{\mathrm{fit}}$ to the column-wise averaged velocities of the droplet centers, keeping the previously determined $a_{1/2}$ and $x_{1/2}$ fixed and focusing on the triangulated region.

10 Packing fraction evolution

The packing fraction of the emulsions is estimated from 2D Voronoi images as shown in Figure 10A-B. Since the equatorial plane used for this quantification is chosen manually, we estimate the error induced by this choice by measuring the local packing fractions in two slices that are located 1 µm above and 1 µm below the chosen equatorial plane. Doing this for two distinct conditions, namely P0/P10 and P6/P10 emulsions (data not shown), yields differences in the average packing fractions measured above and below the identified equatorial planes that are significantly smaller than the standard deviation of the values measured within each plane.

In the case of heterogeneous emulsions (P0/P10) submitted to oscillatory perturbations, the progressive increase of deformation is accompanied by a progressive increase of local packing fraction (quantified for each droplet as the ratio between droplet area and area of its associated Voronoi cell) as observed in Figure 10 (C, middle row) by a continuous shift of the packing fraction distributions towards higher values as the emulsions progress in the channel. Conversely, unperturbed P0/P10 emulsions (Figure 10, C, top row) and homogeneous P10 emulsions (Figure 10, C, bottom row) do not display the same range of evolution.
Alternatively, one can also examine specific emulsions in static acquisitions. As shown in Figure 10 (D), no matter the initial value, heterogeneous P0/P10 emulsions show a significant increase of packing fraction, all tending towards $\phi \sim 1$. In contrast, unperturbed heterogeneous P0/P10 emulsions and homogeneous P10 emulsions exhibit no increase of packing fraction even when the initial packing fraction at the entry of the channel compares to that of their heterogeneous counterpart.

11 Estimation of interface tension ratio from contact angles at triple junctions

Any internal angle between interfaces of an emulsion with heterotypic interface tensions is given by [6]:

$$\cos\theta_1 = \frac{\lambda_1^2 - \lambda_2^2 - \lambda_3^2}{2\lambda_2\lambda_3}, \tag{8}$$

where θ_1 and $\lambda_{1/2/3}$ are defined as indicated in Figure 11 left. In the special case where $\lambda_1 = \lambda_2 = \lambda_3$, the right-hand side evaluates to $-1/2$, which corresponds to the Plateau-rule angle of $\theta_1 = 120°$.

In our heterogeneous P0/10 emulsions, there are four possibilities for the kinds of droplets that meet in a triplet junction: (i) three P10 droplets, (ii) two P10 and one P0 droplets, (iii) one P10 and two P0 droplets, and (iv) three P0 droplets. For cases (i), (iii), and (iv), we would expect equal interface tensions areound the triple junction, and thus Plateau angles of 120°. The only possibility where we would expect heterotypic interface tensions around the triple junction is (ii), two P10 and one P0 droplets (Figure 11 right). In this case, we expect a 10-bp adhesion between the two P10 droplets, which leads to an effective interface tension of λ_{P10}, but no adhesion between P10 and P0 droplets, corresponding to an interface tension of λ_{P0} (Figure 11 right). In this case, we obtain from (8):

$$\frac{\lambda_{\mathrm{P10}}}{\lambda_{\mathrm{P0}}} = \sqrt{2\big(1 + \cos\theta_{\mathrm{P10}}\big)} \qquad \text{and} \qquad \frac{\lambda_{\mathrm{P10}}}{\lambda_{\mathrm{P0}}} = -2\cos\theta_{\mathrm{P0}}, \tag{9}$$

where the angles θ_{P10} and θ_{P0} are defined as indicated in Figure 11 right. Given that we adhesion should reduce reduce the effective interface tension, we expect the upper limit $\lambda_{\mathrm{P10}}/\lambda_{\mathrm{P0}} \leq 1$.

From the segmented snapshots, we quantified the inter-interface angles around each inner vertex by fitting each edge with a straight lines from the junction (see Section 4 for analysis details and Figure 3I for examples).

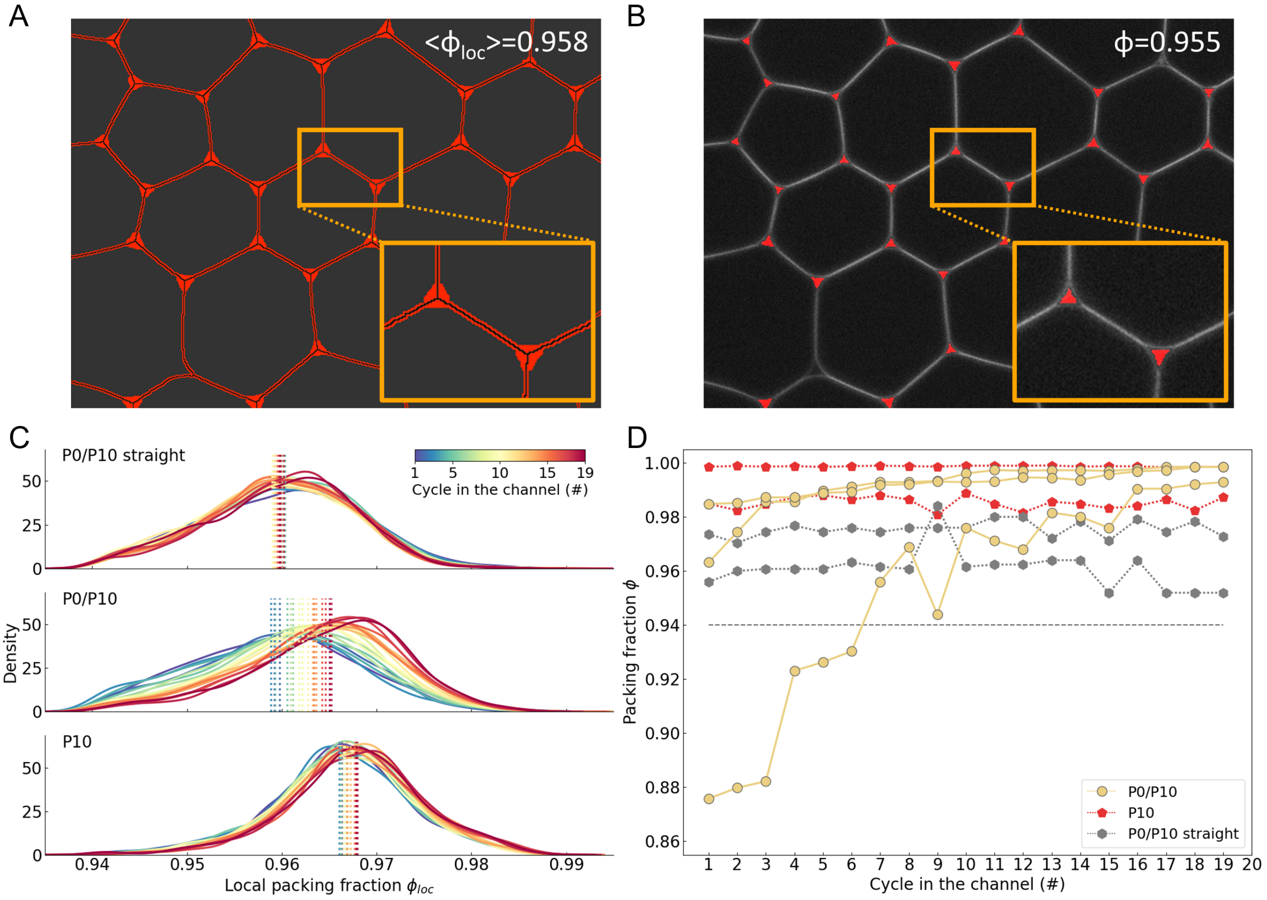

Figure 10: (A) Superimposition of the detected droplets (grey) on the corresponding surface Voronoi tessellation (red). Dividing the droplet area by the area of the corresponding Voronoi cell yields a local packing fraction ϕ_{loc}. On this image, the average local packing fraction is of about 0.958. (B) Packing fraction can also be estimated from the area of the triangles at tri-cellular junctions, which is the method we used in panel D, and to analyze the movies. With this method the packing fraction yields ∼0.955 (on the same image as (A)). (C) Distribution of the local packing fraction in static acquisitions for different experimental conditions (top to bottom: heterogeneous emulsions P0/P10 in a straight channel, heterogeneous emulsions P0/P10 in a wavy channel and homogeneous emulsions P10 in a wavy channel), for all position (purple (position #1) to red (position #19)). Periodically perturbed heterogeneous emulsions exhibit a continuous shift towards higher values of packing fraction along the oscillatory channel until matching the distribution of the homogeneous emulsions in the same channel, which remains constant across positions. In contrast, heterogeneous emulsions in the straight channels exhibits a similar initial distribution as the same emulsions in the wavy channel but no evolution as moving further in the channel. Dashed lines represent the average packing fraction corresponding to each distributions. (D) Examples of the evolution of ϕ across channels measured in static acquisitions. Noticeably, all curves from the P0/P10 emulsions subject to oscillatory shear (P0/P10, yellow circles) exhibit an increase of packing fraction towards a similar high value, independent of the initial packing fraction. In contrast, for unperturbed heterogeneous emulsions (P0/P10 in straight channel, grey hexagons) or homogeneous ones (P10, red pentagons), even with a similar starting point as the P0/P10 perturbed emulsion, the curves remains stable across the whole channel. The grey dashed line at $\phi = 0.94$ represents the cutoff used to filter the data in static acquisitions.

We measure these angles automatically for more than 10^5 tri-cellular junctions in P0/P10 and P10 emulsions in undulated channels, as well as P0/P10 emulsions in straight channels (respectively: 196155,142833 and 220332 angles were measured), all positions of the channel combined. This analysis yields distributions with the following averages and standard deviations: P0/P10 emulsion in undulated channel: $120 \pm 8.85°$, P10 emulsion in undulated channel: $120 \pm 10.48°$ and P0/P10 emulsion in straight channel: $119.98 \pm 8.58°$ (Fig. 12).

For now, our angle analysis code does not take into account the droplet identity. In other words, the measured values of the standard deviation includes all angles in all three-droplet junctions independent of droplet identity. Thus, to turn the obtained results into an upper bound estimate for the interface tension ratio, we first derive an expression for the angle standard deviation σ_θ: The average measured angle is, as

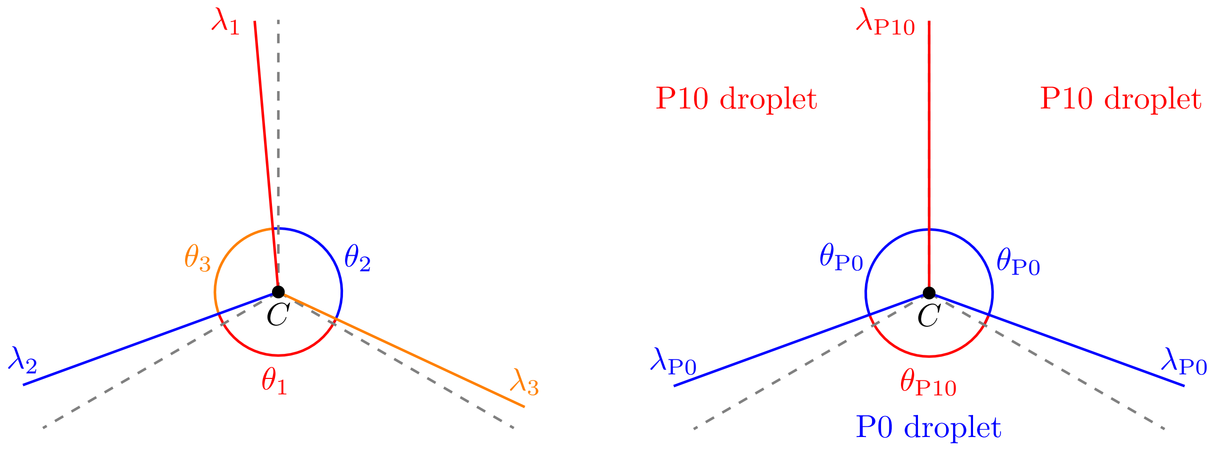

Figure 11: Mechanical equilibrium at the tricellular junction. The dashed gray lines corresponds to the 120°-Plateau rule. (Left) Generic case with three distinct line tensions λ_1, λ_2 and λ_3. (Right) Situation of two P10 droplets and one P0 droplet.

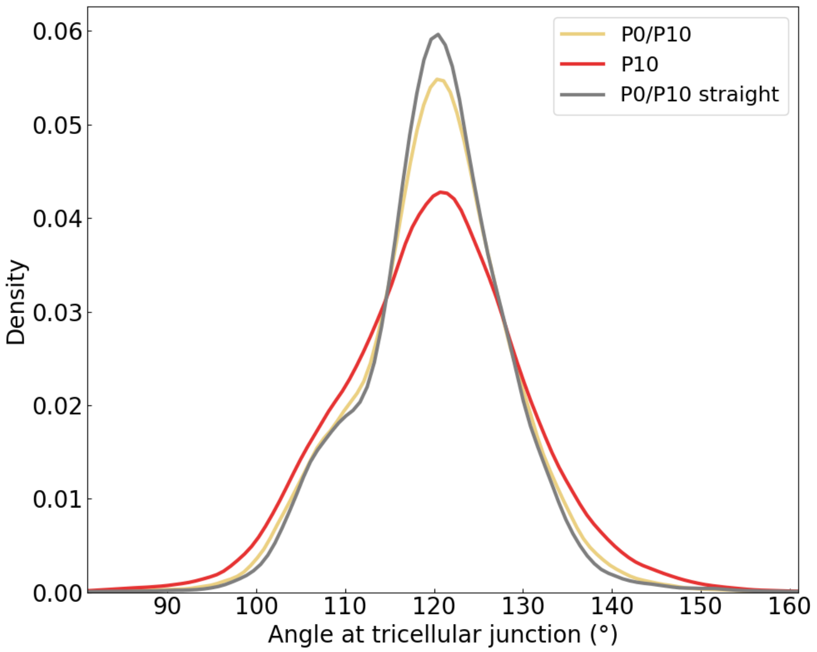

Figure 12: Distributions of measured angles around triple junctions in P0/P10 and P10 emulsions in undulated channels, as well as P0/P10 emulsions in straight channels.

expected 120°. To obtain the expected variance assuming a heterogeneous interface tension, we note that only in the droplet configuration "two P0 and one P10", we expect to see angles other than 120°. In a well mixed sample, this three-droplet configuration appears with a probability of 3/8, and it contributes three angles to the variance: two angles are $\theta_{P0} = 120^\circ - \Delta\theta_0$, with some constant deviation $\Delta\theta_0$, and the third angle is $\theta_{P10} = 120^\circ + 2\Delta\theta_0$ (compare Fig. 11 right). Combined with the fact that our measured standard deviation also includes measurement noise, we obtain the following lower bound: $\sigma_\theta \geq \Delta\theta_0\sqrt{3}/2$. For the P0/P10 emulsion in the undulated channel, we got $\sigma_\theta = 8.85^\circ$, and using (9), we obtain the lower limit for the line tension ratio $\lambda_{P10}/\lambda_{P0} = -2\cos\left(120^\circ - \Delta\theta_0\right) \geq -2\cos\left(120^\circ - 2\sigma_\theta/\sqrt{3}\right) \approx 0.68$.

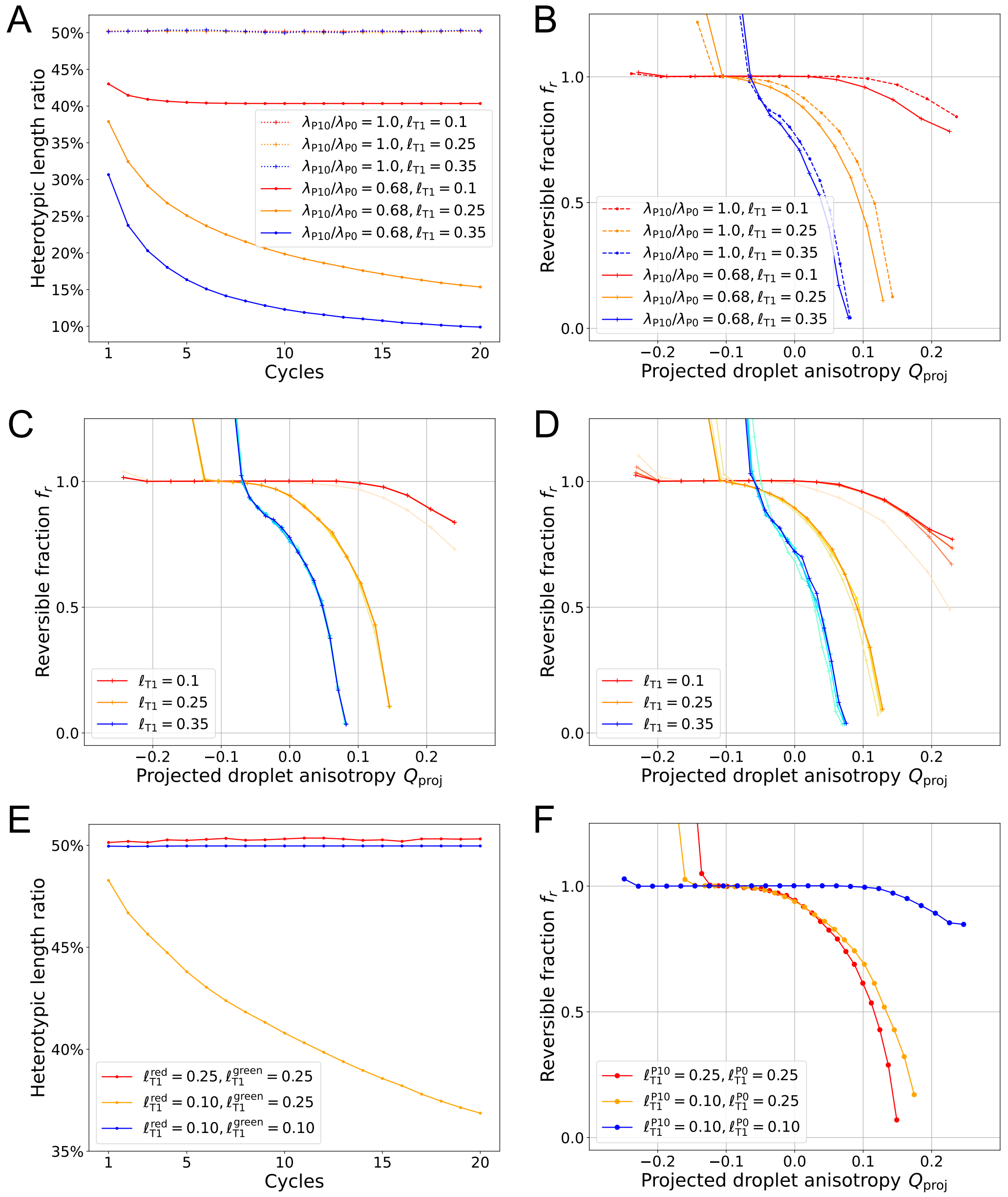

Figure 13: Vertex model simulation results to test whether adhesion directly affects heterogeneous interface tensions or the T1 cutoff. (A) Evolution of the heterotypic length ratio (total length of heterotypic interfaces didived by total length of all interfaces) as a function of the number of shear cycles. The heterogeneous line tension vertex model demixes (solid lines, $\lambda_{P10}/\lambda_{P0} = 0.68$), and demixing speed increases with the value of the T1 cutoff, ℓ_{T1}, as more T1 transitions are allowed. (B) Reversible fraction curves $f_r(Q_{\mathrm{proj}})$ for the simulations in panel A, each averaged over the last 5 out of 20 cycles. The heterogeneous vertex model with $\lambda_{P10}/\lambda_{P0} = 0.68$ (solid lines) yields slightly more easily than its homogeneous counterpart (dashed lines). Moreover, an increase in the T1 cutoff ℓ_{T1} generally leads to an easier yielding. (C,D) Drift in the yielding behavior: reversible fraction curves $f_r(Q_{\mathrm{proj}})$ for different T1 cutoffs (curve color), averaged over cycles: 1-5 (curve of the lightest shade for a given color), 6-10, 11-15, and 16-20 (curve of the darkest shade for a given color). We show both homogeneous (C) and heterogeneous interface tensions with $\lambda_{P10}/\lambda_{P0} = 0.68$ (D). For both homogeneous and heterogeneous interface tensions, and for small T1 cutoff ($\ell_{T1} = 0.1$, red curves), we observe a transient drift in the yielding behavior, roughly over the first 5 cycles (from light to dark shades), where yielding becomes harder over time. This is likely related to some transient relaxation of the initial structural disorder [4]. Such a drift is not observed for higher ℓ_{T1}, possibly because such a structural relaxation may occur much faster in these cases. (E) Evolution of the heterotypic length ratio as a function of the number of shear cycles. We observe that a vertex model with heterogeneous T1 cutoffs demixes (orange curve), contrary to its homogeneous counterparts (blue and red curves). (F) Associated reversible fraction curves f_r, averaged over the last 5 out of 20 cycles. We first observe that the vertex models with homogeneous T1 cutoffs yield easier for larger ℓ_{T1} (consistent with panel B). Second, we see that the yielding behavior for heterogeneous T1 cutoffs (orange curve) is intermediate between the homogeneous cases (blue and red curves), but closer to the one with the larger T1 cutoff. The values for the T1 cutoffs in these simulations were chosen to roughly cover the range that corresponds to our experimental data (see Figure 4C,D in the main text).

12 Estimation of the parameter α

In order to estimate the parameter α in Figure 4D in the main text, we used the `minimize` function from the `scipy.optimize` python library to perform a least-squares fit comparing the packing fraction values ϕ between experiments and vertex model simulations.

To evaluate the square deviation function for a given choice of α, we compute for each experimental data point $(\phi^{\mathrm{exp}}, Q_*^{\mathrm{exp}})$ an interpolated theoretical value ϕ^{interp}. To this end, we first use the vertex model simulation data, which consists of data points $(\ell_{\mathrm{T1}}^{\mathrm{sim}}, Q_*^{\mathrm{sim}})$ to linearly interpolate the $\ell_{\mathrm{T1}}^{\mathrm{interp}}$ value corresponding to Q_*^{exp}. We do this using the `interp` function from the `numpy` python library. Finally, we compute the packing fraction ϕ^{interp} from the interpolated T1 cutoff using $\phi^{\mathrm{interp}} = 1 - (\ell_{\mathrm{T1}}^{\mathrm{exp}}/\alpha)^2$. The function to be minimized is then given by $(\phi^{\mathrm{exp}} - \phi^{\mathrm{interp}})^2$ summed over all experimental data points whose Q_*^{exp} lies between the minimal and maximal Q_*^{sim}.

This fitting procedure yields a value of $\alpha \approx 4.4$.

13 A gradient in packing fraction suggests pumping

13.1 Hypothesis

The continuous phase is incompressible. Hence, under stationary conditions, the total volume flux of the continuous phase should be independent of the position in the channel:

$$h(x)\phi_c(x)\mathrm{v}_c(x) = \mathrm{const.} \tag{10}$$

Here, h is the channel width, $\phi_c = 1 - \phi$ is the packing fraction of the continuous phase, and v_c is the velocity of the continuous phase. Using periodicity of the channel width, $h(x) = h(x + n\lambda)$ for any $n \in \mathbb{Z}$ and λ being the wavelength, we obtain:

$$\phi_c(x)\mathrm{v}_c(x) = \phi_c(x + n\lambda)\mathrm{v}_c(x + n\lambda). \tag{11}$$

Hence, a decrease in ϕ_c across cycles, as we observe it for instance in P0/P10 emulsions, corresponds to an increase in the magnitude of v_c across cycles. Yet, note that in principle, the sign of v_c could in principle be either positive or negative. Yet, preliminary observations using tracer particles suggest that it is positive.

Meanwhile, the same equation as (11) holds also for the discontinuous/droplet phase:

$$\phi_d(x)\mathrm{v}_d(x) = \phi_d(x + n\lambda)\mathrm{v}_d(x + n\lambda). \tag{12}$$

Here, $\phi_d \equiv \phi \simeq 1$, and thus the velocity v_d of the droplet phase is essentially constant among subsequent cycles.

Taken together, a decrease of $\phi_c \ll 1$ corresponds to a change in the velocity of the continuous phase across cycles, while the velocity of the droplet phase is essentially constant. Since there is friction between both phases, such a difference in velocity can only be maintained if there is an effective mechanism that "pumps" both phases with respect to each other, i.e. that drives a non-vanishing relative flow.

13.2 Flow velocity measurements

In order to evidence this potential pumping mechanism, we performed preliminary experiments aiming to analyze the flows of both the droplets and the continuous aqueous phase. To do so, we studied these flows in a heterogeneous P0/P10 emulsion, in which the continuous phase is supplemented with 0.1% v/v of fluorescent particles (FluoSpheres carboxylate, 0.1μm red 580/605, Invitrogen). The emulsion was then injected in the channel and movies were acquired in the red channel at 10 Hz. Note that here the particles were quite big compared to the interdroplet space in a compressed emulsion and therefore acquisitions were performed slightly above the focal plane from which they would be otherwise excluded. In the following we describe the procedure developed in order to analyze those movies and present some preliminary results.

The flow of the continuous phase is analyzed through imaging of the tracers (see Figure 14A and movie S2). We selected a small window of $560 \times 315 px^2$ in the center of the image and applied a threshold in order to isolate the tracers from the background. Since some tracers remained stuck to the glass wall of the channel, we got rid of them but summing all the slices of the movie and by making a mask in which we only kept the dots of highest intensity corresponding to the stuck particles. Subtracting this mask from the original thresholded movie, we obtained a clear binary movie of our tracers that we then tracked using the LAP tracker from Trackmate. We discarded tracks shorter than 1/3 of the track of maximal duration (in terms of timepoints) which represents 3965 tracks. The 246 remaining tracks were then used to compute the speed of the particles in the continuous phase.

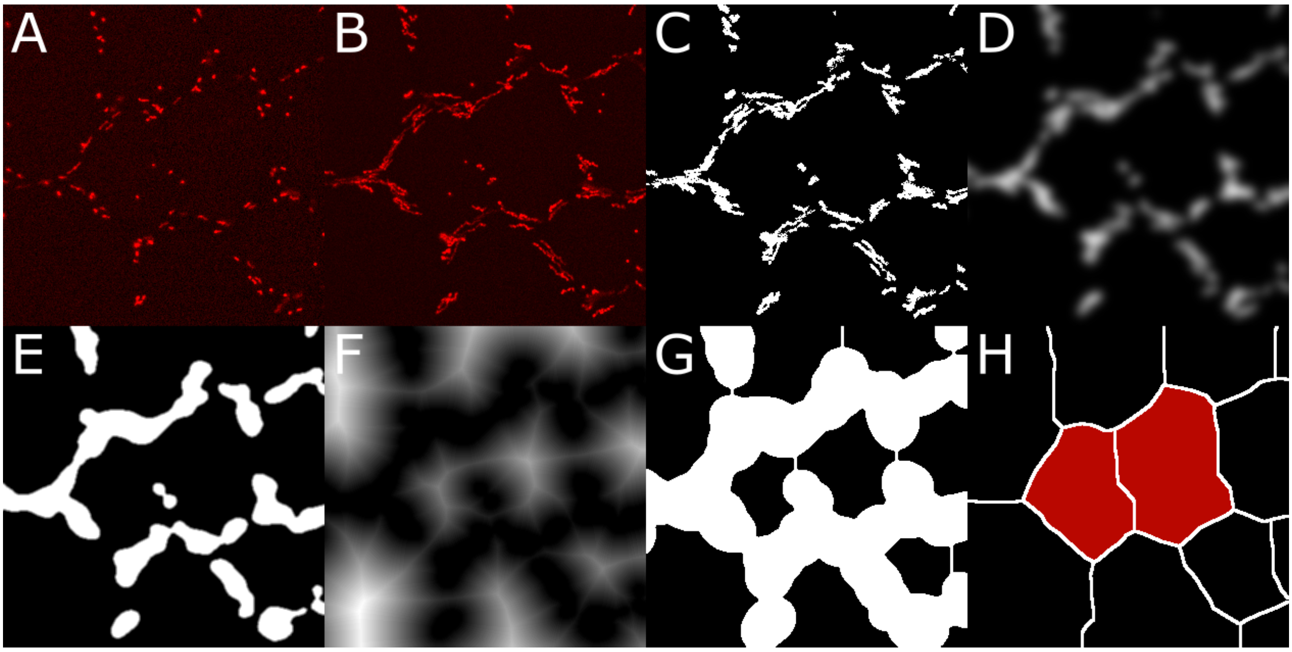

Figure 14: Droplet identification from movies with fluorescent tracers. (A) We define a small window in the middle of the channel on the raw image. (B) Images of the movie are projected five by five to recreate a movie in which each image is the projection of the max intensity of five consecutive images from the original movie. (C) Projections are manually segmented to get a binary image of the tracers in which stuck immobile particles are filtered out. (D) We apply a Gaussian blue filter of 5px to image (C), and threshold this image to obtain (E), from which we perform a distance map of the pixels compared to the identified contours (F). (G) The distance map is thresholded and we apply a watershed on the obtained binary mask. (H) Last, we perform a surface Voronoi tessellation of the mask to get the final contour of the droplets.

Since the droplets could not be imaged together with the tracers, they were only identified from the negative of the particles in the fluid phase. Although this doesn't give the precise contours of the droplets, it allows one to analyze their position and hence their flow. To do so, we first used the "grouped z projection" function from Fiji to obtain a movie in which each image is the projection of the max intensity of five consecutive images (dividing the number of images in the movie by 5, thanks to the relatively low velocity of the droplets this did not impaired the detection of droplets' movement). This process allowed us to get some slightly more continuous contours for the droplets (see Figure 14B). We then applied a manual threshold on this image, removed immobile tracers as described above, and created a binary image of the contours (see Figure 14C). We next applied a Gaussian blur of 5px and once again manually thresholded the image (see Figure 14D-E). After inversion of the image, we created a distance map of our droplets (see Figure 14F). After thresholding, we used the Fiji watershed function to separate groups of droplets identified as one result after this step (see Figure 14G). Finally, we performed a surface Voronoi tessellation which gave us the final contour of the droplets visible in Figure 14H.

In the following, we considered only the droplets that were not touching the borders of the image and that were continuously detected over time (red droplets in Figure 14H). Droplets were tracked using the LAP tracker from Trackmate yielding instantaneous displacements along x and y. As each image was obtained from the projection of 5 consecutive images, in a first approximation we divided by 5 these displacements before computing the speed. From this analysis, we find that the velocity along the x-axis (Figure 15-Left) is two to three times higher for the tracers in the continuous phase than for the droplets, while the velocity along the y-axis is in both cases oscillating around zero (Figure 15-Right). Interestingly, we can observe some spikes of velocity of the droplets both along the x and the y directions that may correspond to T1 events (e.g. around 5, 10, 25 and 30 in Figure 15).

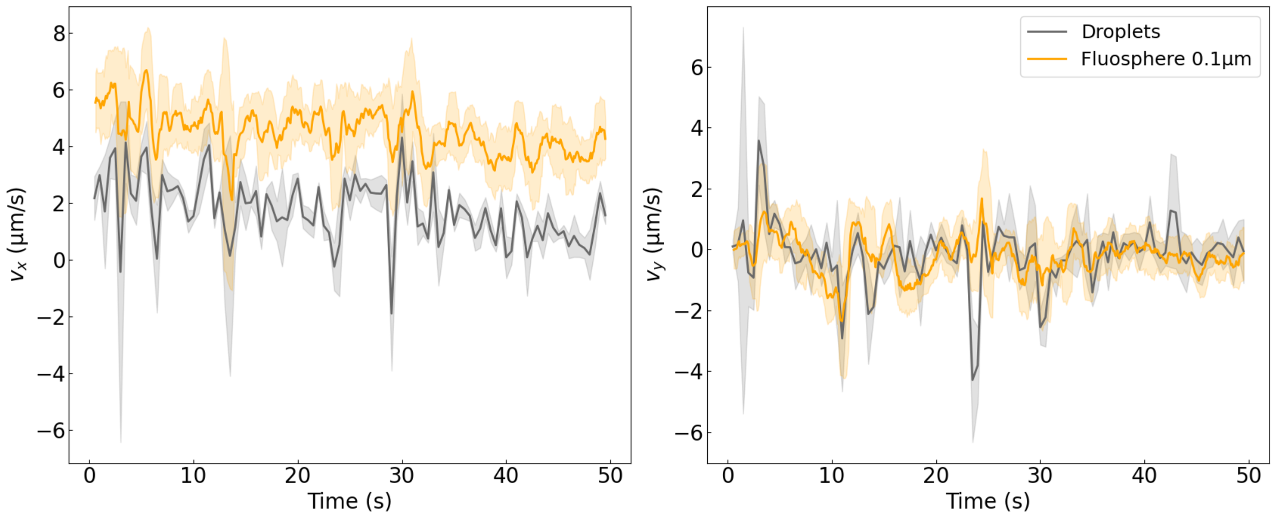

Figure 15: Measured velocity of the droplets and the continuous phase in a P0P10 emulsion. (Left) and (Right) show the velocity along the x and y directions respectively for the tracers (yellow curves) and the identified droplets (grey curves). In the case of the droplets, each point is averaged over all droplets of 5 consecutive timepoints, errorbars represent the standard deviation. For the tracers, each point is an average over all particles combined with a rolling window of 10 frames, errorbars represent the standard deviation of the average in this time-window.

14 Supplementary Videos

14.1 Movie S1: flow of an heterogeneous emulsion

Video showing a P0/P10 emulsion flowing through the fifth undulation of the wavy channel with an average measured speed of $24\mu m/s$. The packing fraction of the emulsion was evaluated at $\phi = 0.997$ using the holes measurement method. Original frame rate is twenty images per second.

14.2 Movie S2: flow of an heterogeneous emulsion with fluorescent tracers in the continuous phase

Video showing a P0/P10 emulsion flowing through the fifth undulation of the wavy channel. Only the small window analyzed in subsection 13.2 is shown. Droplets were functionalized as usual and the continuous phase was supplemented with fluorescent particles (FluoSpheres carboxylate, $0.1\mu m$ red 580/605, Invitrogen) in order to characterize the flow of the continuous phase. Acquisition was performed slightly above the focal plane. Original frame rate is ten images per second.

References

[1] Stuart Berg et al. "Ilastik: interactive machine learning for (bio) image analysis". In: *Nature methods* 16.12 (2019), pp. 1226–1232.

[2] Rémi Dreyfus et al. "Simple quantitative model for the reversible association of DNA coated colloids". In: *Physical review letters* 102.4 (2009), p. 048301.

[3] Lang Feng et al. "Specificity, flexibility and valence of DNA bonds guide emulsion architecture". In: *Soft Matter* 9.41 (2013), p. 9816. ISSN: 1744-683X. DOI: 10.1039/c3sm51586a. URL: http://xlink.rsc.org/?DOI=c3sm51586a.

[4] Alexandre Kabla and Georges Debregeas. "Quasi-Static Rheology of Foams. Part 1. Oscillating Strain". In: *Journal of Fluid Mechanics* 587 (2007), pp. 23–44. ISSN: 14697645. DOI: 10.1017/S0022112007007264.

[5] Matthias Merkel et al. "Triangles bridge the scales: Quantifying cellular contributions to tissue deformation". In: *Physical Review E* 95.3 (Mar. 2017), p. 032401. ISSN: 24700053. DOI: 10.1103/PHYSREVE.95.032401/FIGURES/17/MEDIUM. arXiv: 1607.00357. URL: https://journals.aps.org/pre/abstract/10.1103/PhysRevE.95.032401.

[6] John Shipley Rowlinson and Benjamin Widom. *Molecular theory of capillarity*. Courier Corporation, 2013.
