# Supplementary material for: Adhesion differentials control the rheology of biomimetic emulsions
Source: Biophys J. 2026 Mar 5;125(7):1686–700. doi: 10.1016/j.bpj.2026.03.004 (PMC13351756; doi:10.1016/j.bpj.2026.03.004)
Supplement: Document S2. Article plus supporting material [file mmc4.pdf]

Biophysical Journal
Article

Biophysical Society

Adhesion differentials control the rheology of biomimetic emulsions

Quentin Guigue,[1,2,3] Marc Besse,[1,2] Raphael Voituriez,[1,2] Alexis M. Prevost,[1,2] Elie Wandersman,[1,2] Matthias Merkel,[4,*] and Lea-Laetitia Pontani[1,2,*]

[1]Sorbonne Université, CNRS, Laboratoire Jean Perrin, LJP, Paris, France; [2]Sorbonne Université, CNRS, Inserm, Institut de Biologie Paris-Seine, IBPS, Paris, France; [3]Aix Marseille Univ, CNRS, CINAM, Turing Centre for Living Systems, Marseille, France; and [4]Aix Marseille Univ, Université de Toulon, CNRS, CPT (UMR 7332), Turing Center for Living Systems, Marseille, France

ABSTRACT Animal morphogenesis involves complex tissue deformation processes, which require tight control over tissue rheology. Yet, it remains insufficiently understood how tissue rheology results from the interplay between cellular packing and forces, such as cortical tension or cell-cell adhesion. We follow a biomimetic approach to study this interplay, using oil droplets with tunable adhesion strength to mimic adhesive cells. We expose emulsions to cyclic shear and use a geometric method to quantify their rheology using only imaging data. We find that emulsions made of two droplet types change yielding behavior across subsequent shear cycles. Combining this with vertex model simulations, we show that this shift is due to a progressive compaction, which only occurs with a high adhesion differential and only under oscillatory shear. Our work thus demonstrates how gradients observed during development can lead to gradients in tissue rheology. Moreover, progressive compaction suggests the emergence of a pumping mechanism, which potentially acts in many cellular materials, from foams to tissues.

SIGNIFICANCE During animal development a large number of cells coordinate to progressively mold the organism and its tissues into their adult shapes. Yet, how exactly biological tissues change their shape crucially depends on their mechanical properties. Here we study how tissue mechanical properties depend on individual cell behavior, including cell-cell adhesion. To this end, we mimic tissues using emulsions of oil droplets in water, whose mechanics we probe using oscillatory deformations. Surprisingly, when there are strong adhesion differences between the droplets, the emulsions progressively pack more tightly, changing their mechanical properties across subsequent deformation cycles. Our results point to a mechanism creating cell packing gradients in developing animals, and show how such gradients affect tissue mechanics.

INTRODUCTION

In broad strokes, animal development consists of patterning, i.e., ensuring cells take on the correct biochemical identity in the right places, and morphogenesis, i.e., ensuring tissues deform to obtain their correct adult shapes (1). Changes of tissue shape are driven by internal and external forces (2), but the way these forces translate into tissue deformation crucially depends on the tissue mechanical properties, i.e., tissue rheology. Tissue rheology depends in turn on cellular forces (3–7), cell mechanical properties (8–12), and on the tissue structure (4,13,14), i.e., the way cells are packed within the tissue. Yet, these properties are modulated by the biochemical identities of the involved cells (15). Moreover, whenever forces and deformations are applied to tissues, they could in turn modify the cellular packing. How all these effects combine to affect tissue rheology and thus morphogenesis is still not fully understood.

A cell-scale force that is particularly important for tissue function and development overall is cell-cell adhesion. Specifically, it has been shown to play a key role in the patterning of germ layers (16,17), in shape changes during gastrulation (18,19), in the emergence of epithelial cell polarity (20), and it could play a role during vertebrate axis formation (21). Indeed, synthetic biological systems explicitly demonstrated the capacity of heterogeneous adhesion to separate cell populations (22,23). From a theoretical perspective, understanding such cell population separation by heterotypic adhesion was first discussed by Steinberg

Submitted November 8, 2025, and accepted for publication March 2, 2026.

*Correspondence: matthias.merkel@univ-amu.fr or lea-laetitia.pontani@sorbonne-universite.fr

Editor: Baohua Ji.

https://doi.org/10.1016/j.bpj.2026.03.004

© 2026 The Author(s). Published by Elsevier Inc. on behalf of Biophysical Society.
This is an open access article under the CC BY license (http://creativecommons.org/licenses/by/4.0/).

and co-workers (24,25), where adhesion was proposed to reduce the effective interface tension between tissues. Yet, later studies showed that cell-cell adhesion molecules also affect cortical contractility, substantially amplifying the effect that adhesion has on effective interface tensions (26–29). Moreover, models for biological tissues, such as the vertex model, the Potts model, or continuum models, often describe the effect of adhesion by reducing cell-cell interface tensions (27,30,31). The effect of adhesion can also be captured differently, for instance by means of a higher energy barrier toward separating individual cells (28,32), which would also lead to a higher barrier toward cell rearrangements within a tissue. Such barriers provide another way in which cell adhesion can affect tissue rheology (33). Taken together, while the role of differential adhesion for the separation of cell populations and the related tissue surface tension has been firmly established, a direct effect of differential adhesion on tissue bulk rheology has never been reported to the best of our knowledge.

Here, we study how cell-cell adhesion affects tissue rheology using a well-controlled biomimetic system. We use aqueous emulsions of oil droplets whose adhesions can be tuned to mimic the minimal adhesive and passive mechanical properties of cells in soft tissues, as first described in (34). Of course, our model system cannot precisely mimic all aspects of the mechanics of biological tissues. Instead, our system allows us to study in isolation how the interplay between adhesion and cellular structure affects the material rheology. In previous studies, we used such biomimetic systems to show how adhesion tunes droplet shapes and the material response to a single mechanical perturbation (35–37). However, so far, the role of heterogeneous adhesion on the tissue-scale rheology in such biomimetic systems remains unclear.

In contrast to previous studies with homogeneous adhesion, we here introduce mixtures of different droplet types that are associated with different adhesion strengths, and we study how these differences affect the yielding behavior of the emulsions. Adhesion modulations in tissues have been shown to be critical for developmental processes such as the establishment of tissue boundaries (38) or the formation of functional cellular patterns (39). Our minimal system, in which we control the different binding energies at stake, allows us to explore how such differentials can affect the mechanical properties of the material. Instead of using cadherins as in earlier studies (40), we introduce differential adhesion through distinct DNA binders on two droplet types. This allows us to easily and unambiguously tune the binding energy while keeping the rest of the bond structure the same. We probe the yielding behavior of these emulsions by flowing them through an undulated 2D microfluidic channel to apply oscillatory pure shear deformations. Surprisingly, we find that high adhesion differentials lead to an increase of droplet shape anisotropy across subsequent shear cycles. To understand these observations, we decompose the applied shear into contributions by droplet shape change and droplet rearrangements (41). Here, we extend this method, defining a reversible fraction, f_r, which is the fraction of the overall shear that is created by droplet shape changes. We show that f_r depends mainly on the local droplet shape anisotropy. This relationship can act as a rheological constitutive relation, capturing most of the yielding behavior of the emulsion, which we demonstrate by predicting the observed droplet shape variations. Guided by simulations of a cell-based model, we could show that 1) the yielding behavior of the emulsions strongly depends on the droplet packing fraction and 2) the emulsions with heterogeneous adhesions show a progressive compaction in terms of an increase of droplet packing fraction across subsequent shear cycles. Yet, this compaction occurred exclusively for emulsions with heterogeneous adhesion and only when an oscillatory shear is applied. Taken together, our findings show that an adhesion differential modifies the flow properties of adhesive emulsions, leading to their compaction under repeated shear deformations, an associated shift in their rheological properties, and an increase of droplet shape anisotropy. We expect our work to help better understand dynamic changes that biological tissues undergo during morphogenesis. This includes for instance the formation of packing fraction gradients (4,13,14) and mechanisms creating hydraulic extracellular flows (42).

MATERIALS AND METHODS

Emulsion preparation

All products are obtained from Sigma-Aldrich (Burlington, MA, United States), unless mentioned otherwise. We first prepare an oil-in-water emulsion using a pressure emulsifier (Internal Pressure Type, SPG Technology, Miyazaki, Japan). Silicone oil (50 cSt) is emulsified through an SPG membrane (Shirasu porous glass, hydrophilic, pore size = 10 μm ⌀) in a 10 mM sodium dodecyl sulfate solution (SDS). The obtained emulsion of oil droplets in water can be kept at room temperature for weeks.

The droplets are stabilized with phospholipids through the following procedure (43,44): 9 mg of egg L-α-phosphatidylcholine and 1 mg of DSPE-PEG(2000)-biotin are dried under nitrogen and resuspended in 500 μL of dimethyl sulfoxide. This solution is diluted with 4.5 mL of a 5 mM SDS aqueous buffer (5 mM SDS, 10 mM Tris [pH 7.5]) and sonicated for 30 min at room temperature. Two milliliters of creamed emulsion is then added to this solution, incubated overnight at 4°C, and washed in the morning with 250 mL of the 5 mM SDS buffer in a separating funnel. We repeat this procedure once in a 1 mM SDS buffer (1 mM SDS buffer, 10 mM Tris). The emulsion is finally washed with 250 mL of the 1 mM SDS buffer and stored at 4°C for several weeks. Average diameter $\langle D \rangle = 29.6$ μm; polydispersity index: 13.06%.

Emulsion functionalization

All DNA sequences share the same structure: a biotin molecule, followed by a 49 bp sequence that is common to every strand and that we call the backbone, and a palindromic sticky end sequence whose length ranges from 0 to 14 bp (P0, P6, P10, P14), the backbone and the sticky end being separated by a 1 bp passive spacer. All sequences are detailed in the supporting material.

The chosen binding energies are low enough to allow the droplets to detach during rearrangements without pulling the lipids from the oil/water interface, which is verified by the fact that droplets keep their color integrity throughout all experiments.

Each DNA sequence is prepared separately. First, the backbone is hybridized with a 49 bp complementary sequence (CS) to obtain a stiff double-stranded spacer between the sticky end and the biotin anchor. To do so, 192 pmol of the desired sequence and 192 pmol of CS are dissolved in 200 μL of filtered TS buffer (1 mM SDS, 10 mM Tris [pH 7.5], 10 mM NaCl) in a 0.5 mL DNA Lo-Bind tube (Eppendorf), and incubated at 35°C during 30 min. We then add 5.7 μL of 1 mg/mL streptavidin, Alexa Fluor conjugate (Thermo Fisher Scientific, Waltham, MA, United States) to the DNA sequences and incubate the solution for 30 min in the dark at 35°C. An excess of DNA is used at this step so that all the streptavidins carry at least one DNA strand. This prevents the presence of free streptavidin in solution that could induce droplet adhesion through biotin-streptavidin-biotin bridges. We use streptavidin, Alexa 594, for the palindrome with the longest sticky end (*red droplets* in all images), and streptavidin, Alexa 488, for the other sequence with a shorter sticky end (*blue droplets*). Finally, 100 μL of creamed emulsion, prepared as indicated in the previous section, are added to the DNA solution and incubated in the dark at 35°C for 1 h. Gentle agitation is applied every 20 min to resuspend the emulsion.

After this final incubation the droplets are rinsed 3 times with 200 μL of filtered TS buffer and once with a filtered TS_{gly} buffer (1 mM SDS, 10 mM Tris [pH 7.5], 10 mM of NaCl in a solution of 1:1 w:w glycerol/water). This buffer ensures a better match between the refractive indices of the oil and aqueous phases, which in turn facilitates imaging of the fluorescent droplet edges. Immediately before the experiment, 100 μL of each droplet population are mixed together in 1 mL of filtered TN30S-buffer (1 mM SDS, 10 mM Tris, 30 mM NaCl, 0.05 mg/mL β-casein from bovine milk, in a solution of 1:1 (w:w) glycerol/water [pH 7.5]).

Experimental setup

The microfluidic channels are engineered as described in (35). The whole channel is 30 μm high, which confines the droplets in a 2D monolayer, and consists of three main parts. The first section of the channel is 315 μm wide and 1.65 mm long and is lined with ten 15 μm wide evacuation channels. These evacuation channels allow us to reach reproducible high packing fractions downstream. In the second area, the width of the channel presents 20 oscillations between 315 and 185 μm, imposing 20 shear cycles, with a periodicity of 420 μm. The last area contains a constriction that decreases over a length of 385 μm from a width of 315 to 25 μm. Afterward the channel keeps its width of 25 μm for 583 μm before the final outlet.

Once mounted, the channel is passivated by flowing a solution of casein at 0.25 mg/mL for an hour (β-casein from bovine milk), before injecting the emulsion in the channel using a pressure pump (MFCS-8C, Fluigent, Le Kremlin-Bicêtre, France). The microfluidic device is maintained at a temperature ranging between 18 and 20°C thanks to a custom microscope stage made of PMMA (see supporting material), in which we circulate cooled water from a thermoregulated bath (cooling bath thermostat, CC-K6 Huber).

For static acquisition, once the emulsion is packed inside the channel, we repeat the following protocols for several sets of acquisitions: the emulsion is let flow at low speed for 10 min, the flow is then stopped by progressive decrease of the applied pressure. We image the droplets at all undulations through spinning disk confocal microscopy using a 20× objective (Spinning Disk Xlight V2, Gataca Systems, Massy, France) and finally resume the flow to evacuate the previous emulsion.

For dynamic acquisitions, we acquire movies of 1000 images at 20 Hz in a single undulation of the channel. We image three undulations (5, 10, and 15) and vary the applied pressure, i.e., the flow velocity. The average flow velocities range from ~ 8 to ~ 50 μm/s. It thus takes the droplets between ~8.4 to ~52.5 s to travel across one undulation, which is sufficient to initiate new adhesion between droplets (45). Note that the lower bound of flow velocity roughly corresponds to the one used to flow the emulsion in between two static acquisitions.

Image analysis

The image analysis is performed in the manually detected equatorial plane of the emulsion, allowing us to work with a 2D approach. We analyze separately the channels corresponding to each droplet population. First, images are segmented using Ilastik (46) to separate the fluorescent contour of the droplets from the background. Using a homemade Fiji routine and the segmented images, we create a mask for both channels to identify the droplets and compute a surface Voronoi tessellation of the whole packing (in such tessellation, the whole contour of the droplet is considered to be the seed of the Voronoi cell, which automatically takes in account the polydispersity of the system). We exclude Voronoi cells on the edge of the image, i.e., we exclude droplets on the edge of the channel and partial droplet images. These steps are exemplified in the supporting material. The rest of the analysis is performed using the Sci-kit image Python module. For each image, we start by labeling the Voronoi tessellation and the combination of red/green masks of the droplets. Each droplet is associated to its Voronoi cell and its color. We compute the local packing fraction ϕ_{loc} as the ratio between the droplet area and its Voronoi cell area (Fig. S10 *A*). To determine the error associated to the manual detection of the equatorial plane, we imaged 3D emulsions in two conditions and measured packing fractions above and below the chosen equatorial plane (see supporting material). This analysis leads to relative errors of 0.1–0.4%, which is well below the standard deviation of the local packing fraction distributions within each plane.

Note that, to measure the packing fraction in the dynamic acquisition, we evaluated ϕ from the area of the triangles at tricellular junctions, which corresponds to a similar measurement (Fig. S10 *B*). The positions of the droplets are extracted from the centroid coordinates of a fitted ellipse. In addition, we extract the perimeter p and surface a of each droplet to calculate its corresponding shape factor $\mathcal{A} = p^2/4\pi a$ (see Fig. 2 *A*).

From the segmented images, we identify inner vertices (i.e., vertices involving at least three droplets, thus excluding droplets along the channel border) to triangulate the network as in (41) (see Fig. 2 *B* and details in the supporting material). Triangles elongation is characterized by the tensor $\boldsymbol{Q}$. The triangles and their connectivity are analyzed over time, allowing one to measure the shear contributions by droplet deformation and T1 transitions to compute the reversible fraction f_r.

Prediction of droplet shape

In Fig. 2 *C*, we use quantified reversible fraction curves, $f_r(Q_{\text{proj}})$, to predict the droplet shapes $Q_{xx}(x)$ as the emulsion is pushed through the undulated channel. To this end, we start in our 1D picture from the definition of the reversible fraction, which implies:

$$\frac{\mathrm{d}Q_{xx}}{\mathrm{d}t} = f_r(Q_{\text{proj}})\widetilde{V}_{xx}, \qquad \text{(Eq. 1)}$$

where $\mathrm{d}Q_{xx}/\mathrm{d}t = \partial Q_{xx}/\partial t + \mathrm{v}_x\partial_x Q_{xx}$ with v_x being the x component of the local velocity, and $Q_{\text{proj}} = \mathrm{sgn}(\widetilde{V}_{xx})\, Q_{xx}$. Using stationarity, $\partial Q_{xx}/\partial t = 0$, we thus obtain:

$$\partial_x Q_{xx} = f_r(\mathrm{sgn}(\widetilde{V}_{xx})\, Q_{xx})\frac{\widetilde{V}_{xx}}{\mathrm{v}_x}. \qquad \text{(Eq. 2)}$$

With incompressibility, we have $\widetilde{V}_{xx} = \partial_x \mathrm{v}_x$. Because the log function is monotonously increasing, $\mathrm{sgn}(\widetilde{V}_{xx}) = \mathrm{sgn}(\partial_x \log \mathrm{v}_x)$, implying:

$$\partial_x Q_{xx} = f_r(\mathrm{sgn}[\partial_x \log \mathrm{v}_x]\, Q_{xx})\partial_x \log \mathrm{v}_x. \qquad \text{(Eq. 3)}$$

If the velocity v_x was perfectly homogeneous along the width h of the channel, we would have $h\mathrm{v}_x = \text{const.}$ and thus $\partial_x \log \mathrm{v}_x = -\partial_x \log h$. Yet, we observe that there are some variations of v_x with channel width and

so we use a phenomenological fit to the measured $v_x(x)$, which we then insert into Eq. 3 (see supporting material for details).

To solve Eq. 3, we need to be able to compute f_r for any value of $Q_{proj} = \text{sgn}[\partial_x \log v_x]\, Q_{xx}$. Yet, for the experimentally determined f_r curves, we only have discrete (f_r, Q_{proj}) data points. We thus fit these data points to the phenomenological function $f_r = 1 - 0.5\exp([Q_{proj} - Q_*]/\lambda)$ with fit parameters Q_* and λ. This fit function is then inserted into Eq. 3.

Finally, we integrate Eq. 3 using an explicit Euler method with a spatial stepping of $\Delta x = 1$ μm. We ran the integration for a number of cycles corresponding to the cycle of the experimental observation (i.e., 5 or 15) and compare in Fig. 2 *C* only the last cycle with the experimentally determined $Q_{xx}(x)$ data.

Vertex model simulations

Our vertex model describes a 100% dense packing of $N = 400$ polygonal droplets, where the degrees of freedom are the positions of the polygon corners, called vertices. The mechanics of our model is defined by the following energy functional:

$$E = \frac{1}{2}\sum_{i=1}^{N} k_A (A_i - A_{0i})^2 + \sum_{\langle i,j \rangle} \lambda_{ij} \ell_{ij}. \qquad \text{(Eq. 4)}$$

Here, the first sum is over all droplets i, where k_A is parameter denoting an area elastic modulus, A_i denotes the actual droplet area, and A_{0i} is a droplet-dependent target area. The second sum is over all pairs $\langle i,j \rangle$ of neighboring droplets. These are unordered pairs, i.e., each pair $\langle i,j \rangle \equiv \langle j,i \rangle$ appears only once in the sum. In this sum, λ_{ij} denotes an effective interface tension and ℓ_{ij} is the interface length.

We use periodic boundary conditions, where we apply varying dimensions $L_x \times L_y$, but with a constant total area $L_x L_y = N$, i.e., each cell has on average an area of 1 available to it. We initialize our in silico emulsion as the Voronoi tessellation of a random point pattern. Afterward, we apply cyclic pure shear by setting $L_x = \sqrt{N}\, e^{\gamma}$, where the shear strain γ varies between $\gamma = -\hat{\gamma}$ and $\gamma = +\hat{\gamma}$ with $\hat{\gamma} = 0.27$ and constant shear step $\Delta\gamma = \hat{\gamma}/100 = 0.0027$. The strain amplitude is taken from the experiments, computed as: $\hat{\gamma} = \log(w/[w - 2\Delta])/2 \approx 0.266$, where $w = 315$ μm and $\Delta = 65$ μm (compare Fig. 1 *C*). The value of $\hat{\gamma} = 0.27$ was used in the all simulations (Figs. 4 *C* and S13). All results (Figs. 4 *C* and S13) were averaged over 50–150 simulation runs with different random realizations of the initial conditions.

After each shear step, we quasistatically minimize the energy in Eq. 4 using a custom conjugate-gradient algorithm. During the energy minimization, we test whether the interface length between any two droplets i and j is below the T1 cutoff $\ell_{T1;ij}$. If this is the case, we fuse the two vertices into a many-fold vertex (i.e., a vertex where more than four droplets meet). Moreover, we also test if forces applied to a many-fold vertex allow for the stable formation of a new interface, in which case we split it into a new interface with length $1.5\ell_{T1;ij}$.

We use the following parameter values. We set $k_A = 100$ to enforce an effective incompressibility of the droplets. To match the experimental polydispersity, we draw for each droplet i the target area A_{0i} from a Gaussian with a standard deviation of 0.4 and average of 1, where we impose a lower cutoff of 0.3 to prevent droplets from disappearing. We impose a number ratio of 1 : 1 between P0 and P10 droplets, corresponding to the approximate experimental value (Fig. S5, *left*). For simulations with a homogeneous interface tension, we always use $\lambda_{ij} = 1$ (Figs. 4, *C*, and S13, *C*, *E*, and *F*). For simulations with heterogeneous interface tensions (Fig. S13, *A*, *B*, and *D*), we use:

$$\lambda_{ij} = \begin{cases} 0.68 & \text{if } i \text{ and } j \text{ are both P10} \\ 1 & \text{if } i \text{ or } j \text{ is P0.} \end{cases} \qquad \text{(Eq. 5)}$$

Similarly, for the simulations with heterogeneous T1 cutoffs (Fig. S13, *E*, and *F*), we set $\ell_{T1;ij}$ to 0.1 if both i and j are P10, and to 0.25 if at least one of both is P0.

We realized that, in our vertex model simulations, for large ℓ_{T1} the energy minimization became problematic in the sense that 1) many-fold vertices tended to form (i.e., vertices that abut more than three cells), and 2) there were always new T1 events occurring during the minimization. To accommodate for problem (1), we ran simulations where many-fold vertices were allowed. We tried to also handle (2) by allowing only a given maximal number of three T1 transitions on any given edge or vertex. Yet, this resulted in pathological configurations (e.g., many triangular cells, many concave cells), which is likely due to T1s also getting "stuck" this way, preventing a proper minimization. That there will be problems at large ℓ_{T1} becomes clear already from the fact that we have a substantial area polydispersity with the minimal area cutoff at 0.3. Indeed, e.g., the side length of a hexagon with an area of 0.3 is $\sqrt{2 \times 0.3/3\sqrt{3}} \approx 0.34$. Thus, already from this very optimistic estimate one would expect problems related to "never-ending" T1 transitions at least for $\ell_{T1} \simeq 0.34$.

RESULTS

Introduction of an adhesion hierarchy into biomimetic emulsions

We use biomimetic emulsions that are made of athermal oil droplets dispersed in an aqueous solution (34,48,49). To control droplet-droplet adhesion, DNA strands are grafted onto the droplet surfaces through streptavidin bridges (43,44) (see Fig. 1, *A* and *B*, and materials and methods). Different droplet populations are created by using different DNA sequences, which are distinguished by fluorescently labeling the streptavidin bridges with different colors. Our DNA constructs are all made of a double-stranded backbone of 49 basepairs (bp) followed by a single passive base serving as a flexible junction, and a single strand, the "sticky end" (see Fig. 1, *A*, and *B*; supporting material for the full sequences). In this work we use sticky ends with palindromic sequences, i.e., sequences that bind to themselves. The binding energy of the sticky ends increases with their length (50), and their sequences are chosen such that two DNA strands with different sequences should not bind to each other. This allows us to mix together two populations of droplets that are self-adhesive but do not exhibit any cross adhesion.

Specifically, we work with four distinct constructs: the P0 construct only contains the double-stranded backbone in the DNA sequence, making these droplets nonadhesive; the P6, P10, and P14 constructs are made with sticky ends of lengths 6, 10, and 14 bp, corresponding to binding energies of ~ 8, ~ 13, and $\sim 20 k_B T$, respectively (50). We hereafter use these construct names to refer to the correspondingly functionalized emulsions. We studied homogeneous emulsions (adhesive P10 emulsions or nonadhesive P0 ones), but also mixed emulsions such as P0/P10, P6/P10, P0/P14, and P6/P14, with a 1:1 volume ratio, to probe various adhesion hierarchies. With these mixtures we vary two parameters independently of each other. The average binding energy in the emulsion depends on the sum of the binding

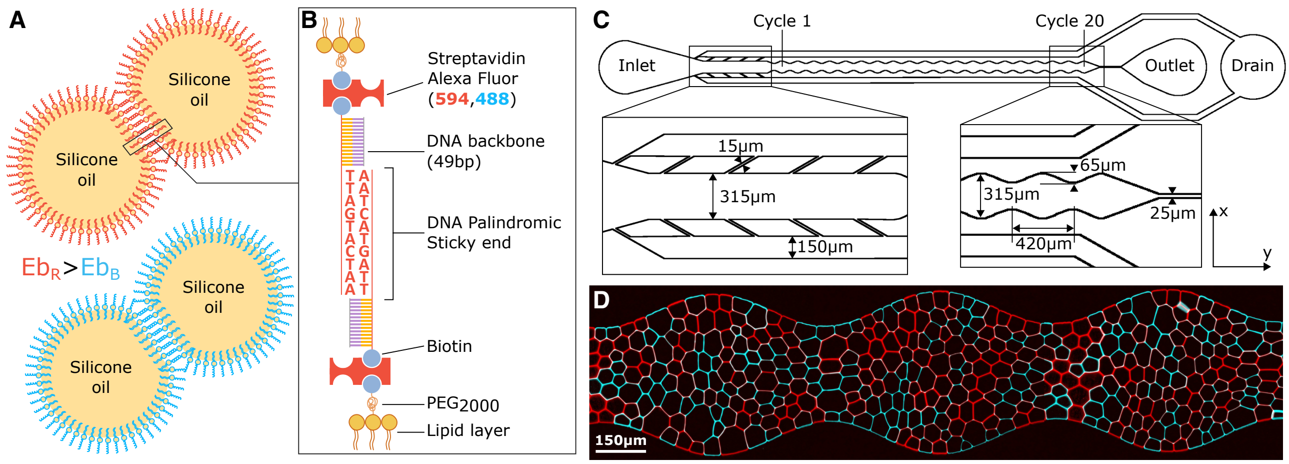

FIGURE 1 (*A*) Schematic representation of DNA-functionalized droplets. Silicone oil droplets are stabilized with SDS, egg L-α-phosphatidylcholine, and biotinylated phospholipids. Two droplets holding the same DNA strand will interact together upon contact with a given binding energy *Eb*, while there is no adhesion between droplets holding different strands. The average diameter of these functionalized droplets is 29.6 μm. (*B*) Biotinylated palindromic DNA strands are grafted on the lipids hydrophilic heads using a streptavidin bridge. Alexa Fluor 594 (*red*) or 488 (*blue*) is attached to the streptavidin, allowing to identify which DNA strand a given droplet carries. (*C*) 2D representation of the microfluidic channel that was designed to apply periodic shear deformation to the emulsions. The channel height is 30 μm, which is adapted to the droplets diameter. Note that the straight channel used for control experiments has the same design, except that the wavy borders are replaced by purely straight wall. (*D*) Stitched confocal image of a P0/P10 emulsion in the wavy microfluidic channel (from *left* to *right*, undulations 11 to 13; *blue*, P0; *red*, P10).

energies associated with each droplet type. In that sense, the P0/P10 mix exhibits a lower average binding energy than the P0/P14 or P6/P10 mixes. Alternatively, one can take into account the difference between the binding strength of the two droplet types, e.g., the adhesion differential is larger for P0/P14 and P0/P10 mixtures as compared with the P6/P10 one.

We flow these emulsions in microfluidic channels that are designed to apply repetitive pure shear deformations to the system (see Fig. 1, *C* and *D*). The total amplitude of each oscillation, which corresponds to a total strain amplitude of $\sim 50\%$, is well within the range of what can also be observed during animal development (51,52), and is sufficient to induce plastic rearrangements (see Video S1 and Fig. 3 *B*). We flow the emulsions sufficiently slowly to ensure that the droplets can re-adhere between constrictions (see materials and methods). Finally, the emulsions are imaged in two ways: either the flow is stopped and static images are acquired at all undulations, or the flow is maintained and a movie is acquired at a given undulation to track droplet dynamics and rearrangements (see materials and methods).

We first seek to understand if there is an effect of adhesion on the structure of the emulsion throughout the channel. To do so, we measure the number and total length of homotypic (*red/red* or *blue/blue*) and heterotypic (*red/blue*) contacts between neighboring droplets on static images. We find that, even with the largest adhesion differentials, the length proportion of heterotypic contacts does not change significantly as the emulsion progresses through the channel (see Fig. S5, *right*). This indicates that segregation between the two droplet populations does not take place over the 20 shear cycles of the channel. However, significant effects are unveiled regarding droplet shapes, as discussed in the next section.

Increase of droplet shape anisotropy along the channel

We explore droplet shape for different adhesion configurations on static acquisitions in the successive undulations. Indeed, adhesive emulsions flowing in constrictions are expected to be deformed as their plastic response is impaired by droplet-droplet adhesion (35,36). Here we study this effect in the presence of repeated shear and heterogeneous adhesion. To do so, we measure the asphericity $\mathcal{A} = p^2/4\pi a$, which compares the perimeter p of a droplet to its cross-sectional area a. It is one for a circular disk, and increases as the droplet shape deviates from that of a circle. We plot the asphericity averaged over all droplets in each undulation cycle of the channel as a function of the cycle number in the channel and for all adhesion conditions (Figs. 2 *A* and S4, *right*). Some conditions exhibit a substantial linear increase of droplet asphericity as the emulsion progresses through the channel. Notably, the sharpest increases are observed for emulsions that exhibit high adhesion differentials between droplet populations such as P0/P10 (*yellow circles*) and P0/P14 (see *pink crosses* in Fig. S4, *right*). This indicates that this increase in droplet asphericity under repeated shear depends on the adhesion differential in the system. Strikingly, the deformation value associated with the lowest average binding energy, i.e., the P0/P10 condition, even surpasses that of the P6/P10 condition after the 13th cycle in the channel (Fig. 2 *A*). This suggests that the presence of an adhesion energy differential can be more important than the

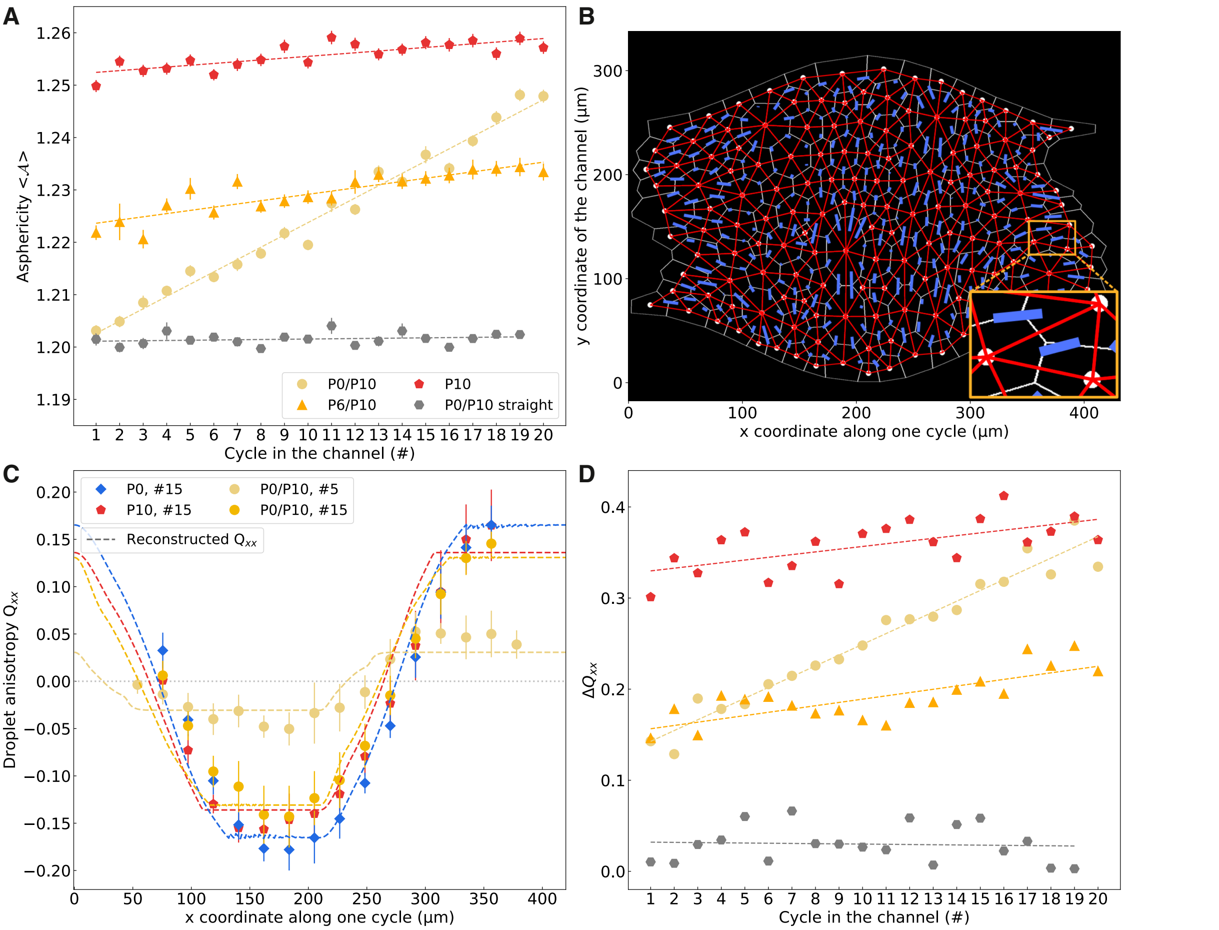

FIGURE 2 (*A*) Average asphericity $\langle \mathcal{A} \rangle$ computed over all the droplets in each undulation cycle in the channel for different conditions (P0/P10 emulsions, *yellow circles*; P6/P10, *orange triangles*; P10, *red pentagons*) or at equivalent cycles in the straight channel (*gray hexagons*). The analysis was performed on static images acquired after the flow was arrested in the channel. Error bars represent the standard error of the mean across experimental repetitions. Each data point was averaged over about 1500 droplets (min = 993, max = 2233, average = 1488). (*B*) Example of a triangulated droplet network (*red*) overlaid on the image of a segmented emulsion. Blue bars represent the orientation and magnitude of $\boldsymbol{Q}$, computed for each triangle (see magnified inset). (*C*) Evolution of Q_{xx} along the x axis for undulations 5 (*yellow circles*) and 15 (*gold circles*) of a P0/P10 heterogeneous emulsion as well as undulation 15 for P10 (*red pentagons*) and P0 (*blue diamonds*) homogeneous emulsions in the oscillatory channel. Values are measured in movies and averaged over time, error bars represent the standard deviation. Dashed lines represent the prediction of $Q_{xx}(x)$, reconstructed for each condition from the reversible fraction and the observed velocity field in the movie. (*D*) Evolution of ΔQ_{xx}, calculated as the amplitude of $Q_{xx}(x)$ variations within a channel oscillation, as a function of the undulation for each experimental condition (same color code as in (*A*)). Similarly to the results displayed in (*A*), P0/P10 (*yellow*) emulsion exhibits a specific linear increase, whereas other conditions display more stable ΔQ_{xx} values along the channel. In the case of the P0/P10 in the straight channel (*gray*), ΔQ_{xx} is constant near 0 as there is no geometrical constraint inducing any ordered anisotropy in the emulsion.

average adhesion energy in creating the progressive increase in droplet asphericity. Note, however, that this increase in asphericity did not correlate with any notable change in polydispersity along the channel.

This is in contrast with previous descriptions of static adhesive droplet packings in which the equilibrium shape of a droplet is given by the balance between the binding energy gain and the energetic cost of surface deformation due to the oil/water surface tension (34). According to these simple considerations, a stronger binding energy automatically induces larger droplet deformations. Here, the rheology of such adhesive emulsions under repeated shear seems incompatible with this static vision. To verify if this increase is indeed due to the applied shear, we use a control experiment in which a P0/P10 mixture experiences a plug flow inside a straight channel with otherwise similar dimensions (see supporting material). In this case, the average droplet shape remains constant throughout the whole channel (Fig. 2 *A*, *gray hexagons*), confirming the central role of repeated shear deformations for the increase of droplet asphericity.

To understand how channel geometry may affect droplet shape, we quantify not only the magnitude but also the orientation of droplet shape anisotropy. Indeed, previous work on foams (53,54), emulsions (35,36), and tissues (41,51,55–59) related the droplet or cell shape and its orientation to the overall material shear deformation. Here, following (41,51), we probe local droplet elongation using a symmetric, traceless tensor $\boldsymbol{Q}$, which quantifies both

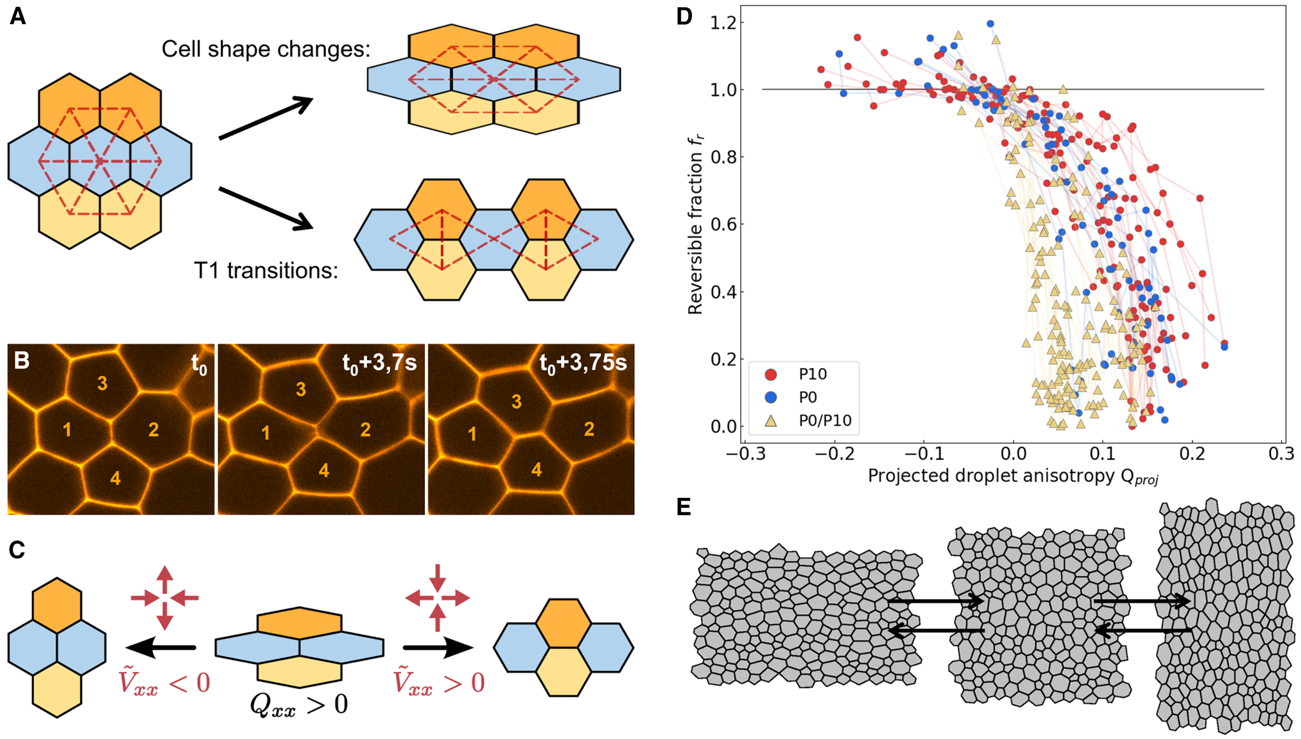

FIGURE 3 (*A*) The observed shear is decomposed into a contribution from cell shape changes (elastic deformation, reversible, *upper right sketch*) and a contribution from T1 transitions (plastic events, irreversible, *lower right sketch*). (*B*) Confocal images of a T1 transition in a P10 emulsion. Droplets 1 and 2, initially neighbors at t_0, are progressively pulled apart ($t_0 + 3.7s$), until they are no longer in contact, letting droplets 3 and 4 become new neighbors ($t_0 + 3.75s$). (*C*) Whether and how much shear is accommodated elastically versus plastically depends on its direction. If shear is oriented perpendicular to droplet reorientation (*left arrow*) one expects purely elastic deformation. If shear is oriented parallel to droplet shape, a part of the shear may be accommodated by plastic rearrangements. (*D*) Reversible fraction as a function of the projected elongation $Q_{\text{proj}} = \text{sgn}(\widetilde{V}_{xx})\, Q_{xx}$. All curves are labeled according to the experimental conditions (*red*, P10 emulsions; *blue*, P0 emulsions; *yellow*, P0/P10 emulsions), independently of channel undulation. Each curve represents the analysis of one movie. (*E*) Schematic of vertex model simulations. We use periodic boundary conditions, whose dimensions are modified to impose oscillatory pure shear deformations at a constant total area.

magnitude and orientation of droplet shape anisotropy. To this end, we first triangulate the emulsion by connecting the centers of neighboring droplets by triangles (Fig. 2 *B*, *inset*), leading to a triangulation of the whole emulsion without gaps or overlaps (Fig. 2 *B*, details in supporting material). For a given triangle, the magnitude of $\boldsymbol{Q}$ corresponds to $\log(\text{AR})/2$, where AR is the aspect ratio of an ellipse fitted to the triangle, and the orientation of the tensor $\boldsymbol{Q}$ corresponds to that of the long axis of the ellipse. For any given region of the emulsion, we define the local droplet shape as the area-weighted average of the triangle-based $\boldsymbol{Q}$ tensors (details in the supporting material).

In Fig. 2 *C*, we show examples of time-averaged Q_{xx}, which is the horizontal component of $\boldsymbol{Q}$, versus the position x along the channel within one undulation, averaged across the channel width y (see Fig. 2 *B*). A positive horizontal droplet shape component, $Q_{xx} > 0$, indicates a droplet elongated along the channel direction, whereas a negative component, $Q_{xx} < 0$, indicates a droplet elongated perpendicular to the channel direction. We find that Q_{xx} oscillates with positive values, i.e., horizontally aligned droplets, where the channel is narrow, and negative values, i.e., vertically aligned droplets, where the channel is wide (Fig. 2, *B* and *C*). We furthermore quantified $\Delta Q_{xx} := Q_{xx}^{\max} - Q_{xx}^{\min}$, the difference between maximal and minimal values of Q_{xx} within a single channel undulation (Figs. 2 *D* and S4, *left*), and find that it behaves very similarly to the asphericity $\mathcal{A}$ (Fig. 2 *A*). Specifically, we observe a substantial shift for emulsions with a large adhesion differential (Fig. 2, *A* and *D*), which for P0/P10 corresponds to an increase of the droplet aspect ratio from ≈1.15 at the beginning of the channel to ≈1.40 at the end of the channel.

Geometric analysis of the yielding behavior

To understand the evolution of the droplet shape anisotropy Q_{xx} for different emulsions both with and without oscillatory shear, we study the yielding behavior of these emulsions. To this end, we use the fact that any anisotropic deformation of a cellular material can be decomposed into contributions by cell shape changes and by cell rearrangements, so-called T1 transitions (Fig. 3, *A* and *B*). Specifically, for a given set of droplets that we experimentally track over time, we use the formalism from (41) to

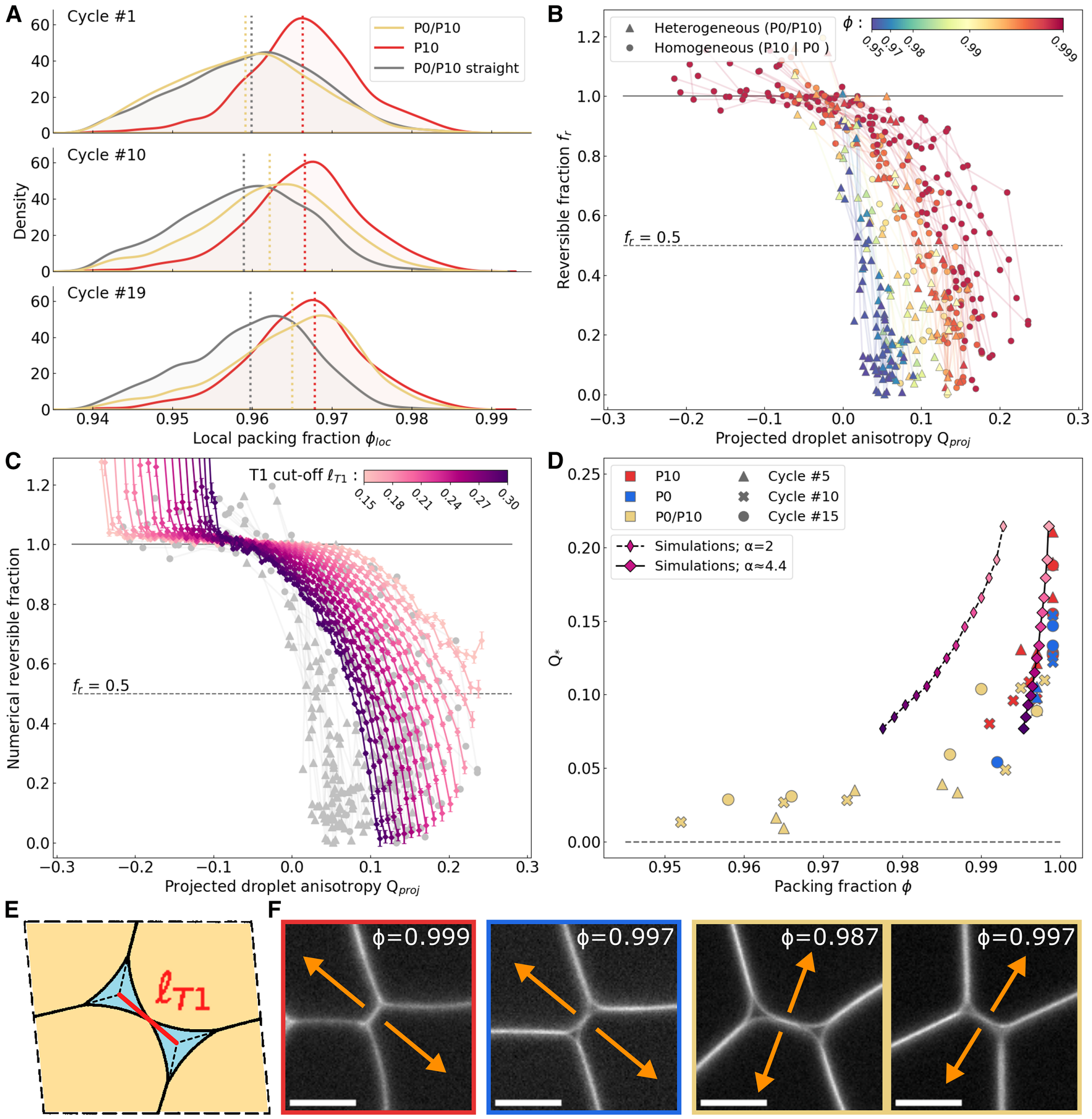

FIGURE 4 (*A*) Distribution of the packing fraction in static acquisitions for different experimental conditions and undulations in the channel. Heterogeneous emulsions (*yellow*, P0/P10) exhibit an overall shift toward higher values of packing fraction along the oscillatory channel (from *top*, undulation 1, to *bottom*, undulation 19), while heterogeneous emulsions in the straight channels (*gray*) start at the same value without exhibiting any evolution. The distributions of packing fraction for homogeneous emulsions in undulated channels (*red*) also remain constant across undulations. Vertical doted lines represent the mean value of each distribution. (*B*) Reversible fraction as a function of the projected droplet shape elongation Q_{proj}, colored according to the packing fraction ϕ measured for each movie. Triangles: heterogeneous P0/P10 emulsions; circles: homogeneous P0 and P10 emulsions. (*C*) Reversible fraction computed from vertex model simulations at different T1 cutoff length ℓ_{T1}. In gray, we reproduce the experimental curves from (*B*). (*D*) Evolution of Q_* as a function of the packing fraction ϕ for experiments and numerical simulations. In the case of numerical simulations, the T1 cutoff lengths ℓ_{T1} have been converted into packing fractions using Eq. 9, using either Princen's prefactor $\alpha_0 = 2$ or $\alpha \approx 4.4$, obtained through squared error minimization (see supporting material). Error bars represent the standard error of the mean across experimental repetitions. (*E*) Representation of the T1 cutoff length ℓ_{T1} (*red line*), defined at the moment when two tricellular junctions (*blue regions*) meet and are about to merge, thus triggering the T1 event. ℓ_{T1} is defined as the distance between the two tricellular junctions' centers in this geometry (adapted from (47)). (*F*) Last snapshot of the droplet-droplet interface before a T1 event for homogeneous (P10, *red border*; P0, *blue border*) and heterogeneous emulsions (P0/P10, *yellow borders*), 50 ms before the contact has disappeared (i.e., before the next snapshot). Scale bar, 10 μm. Arrows represent the direction of the T1 (i.e., of the separation between previously contacting interfaces).

decompose the rate tensor (i.e., the anisotropic part of the strain rate tensor) $\widetilde{\boldsymbol{V}}$ as follows:

$$\widetilde{\boldsymbol{V}} = \frac{\mathrm{D}\boldsymbol{Q}}{\mathrm{D}t} + \boldsymbol{R}. \qquad \text{(Eq. 6)}$$

with $\widetilde{\boldsymbol{V}}$ being the shear rate tensor averaged over the tracked region, which is decomposed into the rate of change of the average droplet shape anisotropy $\boldsymbol{Q}$, where $\mathrm{D}/\mathrm{D}t$ is an advective, corotational derivative, and a contribution from

T1 transitions $\boldsymbol{R}$ occurring within this region (Figs. 3 *A* and S8, *A* and *B*). In other words, the first term on the right-hand side in Eq. 6 represents reversible, elastic contributions to shear, while the second term represents irreversible, plastic contributions. Note that we slightly simplify Eq. 6 compared with (41). First, we neglected contributions that appear only in biological tissues, such as cell divisions. Second, for simplicity, we choose a quasi-1D description, neglecting the corotational contribution, and focusing only on the components along the channel direction:

$$\widetilde{V}_{xx} = \frac{\mathrm{d}Q_{xx}}{\mathrm{d}t} + R_{xx}, \qquad \text{(Eq. 7)}$$

where $\mathrm{d}/\mathrm{d}t = \partial Q_{xx}/\partial t + \mathrm{v}_x(\partial Q_{xx}/\partial x)$ denotes the advective derivative, with v_x being the x component of the local velocity. Note that, in our emulsions, we can measure all terms in Eqs. 6 and 7, where corotational effects are negligible (see Fig. S8).

The mechanical relaxation rate of our emulsions is much faster than the shear rate that we impose through the undulations, i.e., the emulsions are deformed quasistatically (see Fig. S6). This condition of quasistatic deformation allows us to go beyond the formalism of (41). Since the shear timescale does not play any role, we divide Eq. 7 by $\widetilde{V}_{xx}$:

$$1 = f_r + f_i, \qquad \text{(Eq. 8)}$$

where $f_r = (\mathrm{d}Q_{xx}/\mathrm{d}t)/\widetilde{V}_{xx}$ is the fraction of the overall shear that is due to reversible (i.e., elastic) droplet shape deformations, and $f_i = R_{xx}/\widetilde{V}_{xx}$ is the fraction of the overall shear that is due to irreversible (i.e., plastic) droplet rearrangements. If we can understand for each of our emulsions what fraction of the applied strain changes droplet shapes, f_r, versus what fraction is accommodated by droplet rearrangements, $f_i = 1 - f_r$, we will be able to predict droplet anisotropy Q_{xx} as the emulsion is pushed through the undulating channel. In other words, knowing the behavior of f_r amounts to knowing a constitutive relation. Note that, while the stress tensor does not explicitly appear in these equations, it can be easily computed from the droplet shape anisotropy and the interface tensions using the Bachelor formula (60–62).

In general, the reversible fraction f_r will depend on the state of the emulsion, particularly on the average droplet shape anisotropy Q_{xx}. Yet, it likely also depends on whether the emulsion is sheared parallel to the droplet elongation axis, i.e., $\widetilde{V}_{xx}Q_{xx} > 0$, or perpendicular to the droplet elongation axis, i.e., $\widetilde{V}_{xx}Q_{xx} < 0$ (Fig. 3 *C*). Specifically, in a simple picture, one would expect that if the emulsion is being sheared parallel to $\boldsymbol{Q}$ (Fig. 3 *C*, *right arrow*), this would in part increase droplet elongation and in part lead to droplet rearrangements. Meanwhile, when the emulsion is sheared perpendicular to $\boldsymbol{Q}$ (Fig. 3 *C*, *left arrow*), one would just expect that the droplet shape relaxes toward a less-elongated state, while almost no droplet rearrangements are expected to occur. Thus, we expect f_r to depend mostly on the projection of Q_{xx} on the shear direction, $Q_{\mathrm{proj}} := \mathrm{sgn}(\widetilde{V}_{xx})\, Q_{xx}$, where $\mathrm{sgn}(\widetilde{V}_{xx})$ denotes the sign of $\widetilde{V}_{xx}$.

To measure how the reversible fraction f_r depends on the projected droplet shape Q_{proj} for different emulsions, we acquired movies for conditions that exhibit the most salient differences: nonadhesive P0 homogeneous emulsions, adhesive P10 homogeneous emulsions, and P0/P10 mixtures of droplets. The movies were acquired in different undulations of the channel (5, 10, and 15). Note that droplets in these experiments are all labeled with the same fluorophore, even in the case of heterogeneous P0/P10 emulsions, to acquire the movies at a high enough frame rate so that individual droplets can be tracked. To measure the reversible fraction function $f_r(Q_{\mathrm{proj}})$ in these movies, we essentially divide each image into stripes across the whole channel height and with a width of 96 pixels ($\simeq 21.5$ μm). For each stripe, we quantify the average triangle elongation Q_{xx} and the shear rate $\widetilde{V}_{xx}$, which we average over all time points (see supporting material for details). We also check that for any given position, droplet shapes are essentially stationary over time (see supporting material, *green curve* in Fig. S8). We then compute the projected shape and reversible fraction for that stripe as $Q_{\mathrm{proj}} = \mathrm{sgn}(\langle \widetilde{V}_{xx}\rangle_t)\,\langle Q_{xx}\rangle_t$ and $f_r = \langle \mathrm{d}Q_{xx}/\mathrm{d}t\rangle_t / \langle \widetilde{V}_{xx}\rangle_t$, respectively, where $\langle\cdot\rangle_t$ denotes an average over time.

The resulting $f_r(Q_{\mathrm{proj}})$ curves for all experimental conditions are presented in Fig. 3 *D*, in which we pooled under the same label all the movies acquired at all undulations for one type of emulsion. The curves associated to the P0/P10 emulsions appear shifted to the left with respect to the homogeneous P0 and P10 emulsions: at a fixed value of $f_r < 1$, these emulsions yield at a lower value of Q_{proj}. In other words, for them to yield, the P0/P10 emulsions do not need to be deformed much, i.e., one does not need to elongate the droplets very much until they start to rearrange.

Note that we also observe values of $f_r > 1$ for the smallest $Q_{\mathrm{proj}} < 0$ (Fig. 3 *D*). By definition, a value of $f_r > 1$ means that droplet shape elongation in these cases is even larger than the shear strain, which essentially implies that T1 transitions occur perpendicular to the external shear direction. We make very similar observations in simulations of our emulsions (see below, Fig. 4 *C*). In experiments and simulations, these perpendicular T1 transitions occur for negative Q_{proj} right after the reversal of the shear direction. Our simulations indicate that they represent regions that were in the process of undergoing a T1 transition during the shear reversal (Fig. S8 *C*). The existence of these T1 transitions indicates a refinement of the simple picture given above, which suggests that there should be no T1 transitions for $Q_{\mathrm{proj}} < 0$ (more details in supporting material, section 8.5).

So far, we have hypothesized that f_r depends only on the local projected cell shape, Q_{proj}. Yet, there are also other possibilities, e.g., f_r could additionally depend on other aspects of the local emulsion packing structure. Moreover, f_r could also be affected by nonlocal effects, e.g., strain steps

elicited by T1 transition in other regions of the emulsion that are propagated through long-range elastic interactions (63) (more details in supporting material, section 8.5). If f_r depended mostly only on the local Q_{proj}, then $f_r(Q_{\text{proj}})$ should play the role of a constitutive relation and it should be possible to predict the measured $Q_{xx}(x)$ curves knowing only $f_r(Q_{\text{proj}})$ and the applied shear protocol (see materials and methods and supporting material). If this hypothesis was wrong, and other dependencies of f_r also played an important role, the reconstruction of $Q_{xx}(x)$ based on $f_r(Q_{\text{proj}})$ should fail. In Fig. 2 *C*, we compare the $Q_{xx}(x)$ curves predicted based on $f_r(Q_{\text{proj}})$ (*dashed lines*) to the measured ones (*circles*, *diamonds*, and *pentagons*). We find good agreements between reconstructed and measured $Q_{xx}(x)$ for most of the emulsions. Thus, a single dependency of f_r on Q_{proj} alone captures most of the observed $Q_{xx}(x)$ behavior. This includes specifically the shift in ΔQ_{xx} across shear cycles in the P0/P10 emulsions. This strongly suggests that the observed shift in droplet asphericity in heterogeneous emulsions is due to a shift in the $f_r(Q_{\text{proj}})$ curves toward higher Q_{proj} values.

Simulations exclude heterogeneous interface tensions or energy barriers to T1 transitions as reasons for the observed shift

To study possible reasons for the shift in the yielding behavior, we carried out simulations of 2D vertex models (see materials and methods) (64–69). Vertex models describe emulsions, foams, and biological tissues as polygonal tilings, where each polygon corresponds to one droplet. We subject the vertex model to an oscillatory pure shear, where, after each shear step, we quasistatically minimize the system energy stemming from the droplet-droplet adhesion. Droplets are allowed to rearrange if a droplet-droplet contact shrinks below a length given by a T1 cutoff parameter ℓ_{T1}, defined in terms of the average droplet area. Using a mixture of two kinds of droplets, we studied whether a shift in the yielding behavior could be created by adhesion heterogeneities, where we tested two different ways of representing adhesion in our simulations.

First, we tested whether adhesion heterogeneities could create substantially different droplet-droplet interface tensions, and whether this could create the observed shift. To experimentally obtain the interface tension ratio in our P0/P10 emulsions, we measured the contact angles between interfaces at three-droplet junctions and found an interface tensions ratio of at most 1 : 0.68 (see supporting material and Fig. S11). Using this maximally possible tension ratio, we ran simulations with heterogeneous interface tensions, and compared them with the case of homogeneous interface tensions. Indeed, there was a shift across subsequent shear cycles in the yielding behavior of the heterogeneous in silico emulsions (Fig. S13 *D*), as in the experiments. Yet, the amount of shift was small, and we observe the same amount of shift in the homogeneous in silico emulsions (Fig. S13 *C*) in contrast to the experiments. This is due to a known effect, where under oscillatory shear the structure of the emulsion shows an initial transient evolution (62). Furthermore, comparing Figs. 3 *D*, S5, *right*, and S13, *A* and *B*, we find that unmixing and yielding behavior of heterogeneous in silico emulsions is inconsistent with our experimental data. In short, in silico emulsions unmix much faster and/or yield less easily than our experimental emulsions. Taken together, the modulation of interface tensions by adhesion cannot explain the difference in shift that we observe between homogeneous and heterogeneous emulsions.

Second, given that adhesion also sets a barrier for droplet-droplet detachment, we tested whether heterogeneities in T1 transition barriers could create the observed shift. In the simulations, we describe the T1 transition barrier by the T1 cutoff length ℓ_{T1}. The most adhesive emulsions would thus be associated to smaller values of ℓ_{T1} compared with less-adhesive ones. In this scenario, the homogeneous, less-adhesive in silico emulsions yield more easily than the more adhesive ones (Fig. S13 *F*), which is in contradiction with our experimental observations (Fig. 3 *D*). Moreover, the reversible fraction function of the heterogeneous in silico emulsion lies in between the two homogeneous cases (Fig. S13 *F*), again in contrast to our experimental observations. Taken together, homogeneous emulsions with a lower barrier for T1 transitions will yield more easily, suggesting that T1 barrier heterogeneity cannot play a major role in our emulsions.

Progressive compaction explains the shift in yielding behavior

To understand what else might cause the observed shift in yielding behavior, we noted that, in the simulations, the T1 cutoff ℓ_{T1} strongly affected the yielding behavior (Fig. S13). While this parameter is not substantially tuned by adhesion (see previous section), it is expected that the barrier toward T1 transitions depends on the packing fraction ϕ, i.e., the area fraction of oil within the emulsion. Indeed, for a lower packing fraction, one would expect the droplets to rearrange more easily. To relate the T1 cutoff and packing fraction, Princen considered the triangular regions of continuous phase where three droplets meet (*blue regions* in Fig. 4 *E* as example) (47). He found:

$$\ell_{\text{T1}} = \frac{\alpha\sqrt{1-\phi}}{\sqrt{\rho}}, \qquad \text{(Eq. 9)}$$

where ρ is the average area number density of the droplets (in the simulations, $\rho = 1$). Crucially, Princen assumed that droplets would rearrange exactly at the moment when two triangular regions meet (Fig. 4 *E*). For ordered packings

of nonadhesive, monodisperse, hexagonally packed droplets, this implies $\alpha = \alpha_0 \equiv 2/\sqrt{3(2\sqrt{3} - \pi)} \approx 2.0$ (47).

We therefore measured the local packing fractions in our emulsions. Indeed, the heterogeneous P0/P10 emulsions display an increase in packing fraction across subsequent shear cycles (see *yellow curves* in Fig. 4 *A*), while the packing fraction distribution is maintained throughout the channel for homogeneous emulsions (*red curves*). Furthermore, heterogeneous P0/P10 emulsions that are not exposed to cyclic shear also show a roughly constant packing fraction distribution (*gray curves*). This suggests that the adhesion differential modifies the flow properties of our emulsion such that the water phase can be progressively expelled under the cyclic shear, thus increasing its packing fraction. Yet, this progressive compaction only occurs when both an adhesion differential and a repetitive strain are acting together.

To study the effect of the packing fraction on the yielding behavior, we color the reversible fraction curves from Fig. 3 *D* as a function of the packing fraction (Fig. 4 *B*). Indeed, we find a clear trend where, as the packing fraction increases, emulsions yield less easily, i.e., the droplets need to be deformed up to higher Q_{proj} until yielding. We compare these results with vertex model simulations, where we vary the T1 cutoff from $\ell_{\text{T1}} = 0.15$ to 0.3 (Fig. 4 *C*). We note that the f_r curves of our in silico emulsions have a similar qualitative shape as our experimental curves. Furthermore, the in silico emulsions yield less easily as the T1 cutoff is decreased.

To compare experiments and simulations more directly, we quantify the droplet shape Q_* where the reversible fraction is $f_r(Q_{\text{proj}} = Q_*) = 0.5$ (see *gray dashed lines* in Fig. 4, *B* and *C*, and supporting material). In Fig. 4 *D*, we plot Q_* as a function of the packing fraction ϕ. We first note that, when plotting our experimental data against ϕ, strikingly, our data almost collapse to a master curve, which means that there is no significant effect of adhesion hierarchy on yielding (*blue* versus *red* versus *yellow* data points). This observation is a central result of our work. We compare this experimental data with simulations (shades of *magenta*), where the packing fraction ϕ is computed from the T1 cutoff ℓ_{T1} using Eq. 9 with different values of α. For the packing fraction range $\phi \sim 0.97\ldots0.99$, covered by our experiments, the idealized value $\alpha_0 \approx 2.0$ captures qualitatively the experimentally observed trend but does not match quantitatively. Our experimental data are actually best fitted with $\alpha \sim 4.4$ (see supporting material). This indicates that the simple criterion from Princen is insufficient to capture our experimental findings, and yielding can occur already before the two continuous-phase triangular regions meet (see Fig. 4 *E*). Note that the vertex model simulations do not capture the experimental trend at low packing fractions ($\phi \leq 0.98$), i.e., high T1 cutoff length, which is due to the fact that vertex models are inherently not designed to model wet foams (see materials and methods).

To independently assess whether T1 fusions may occur before triangular regions touch, we created snapshots of droplet-droplet interfaces immediately before a T1 transition is about to happen (Fig. 4 *F*). In all cases, and for different kinds of emulsions, we find that droplet-droplet interfaces still have a length on the order of the size of the triangular regions immediately before the T1 transitions are triggered, quite different from the scenario assumed by Princen (Fig. 4 *E*). Because of the time resolution of our movies, these observations cannot be considered as a quantitative measurement of ℓ_{T1}. Nevertheless, they are in qualitative agreement with our findings in Fig. 4 *D*. This indicates that there could be a—so far unknown—instability through which droplet-droplet interfaces may already undergo a fast collapse at a finite length.

DISCUSSION

Here we studied the impact of adhesion architecture on the mechanical properties of biomimetic emulsions. In contrast to earlier work (35–37), we compared emulsions with homogeneous adhesion to emulsions composed of two distinct droplet populations, each with their own adhesion strength. We applied cyclic shear to these emulsions and developed a geometric framework to characterize their elastoplastic properties. We showed that the emulsions with an adhesion differential changed their yielding behavior across shear cycles. Comparing this approach with vertex model simulations revealed that this shift in yielding behavior was due to a progressive compaction.

It has long been known that viscosity, stiffness, and yielding behavior of particulate matter are closely linked to the packing fraction (70–73). Yet, here we demonstrated that for emulsions this relation can be quantified using imaging data only. To this end, we combined for the first time emulsion data with a theoretical method to decompose material-scale deformation into contributions by droplet-scale processes. Specifically, we extended the geometric formalism of (41) to the quasistatic limit by defining a reversible fraction function f_r, which corresponds to the fraction of shear created by droplet shape changes alone. We showed that the reversible fraction f_r depends mostly only on the local projected droplet shape, Q_{proj}, by reconstructing the observed droplet shape changes from the function $f_r(Q_{\text{proj}})$. This means that $f_r(Q_{\text{proj}})$ can be considered as a constitutive relation describing the yielding of the emulsion. While the stress tensor does not explicitly appear, it can be directly computed from the droplet shape tensor $\boldsymbol{Q}$ knowing the interface tensions (60–62,74). Our approach is quite general and relies only on the assumption of quasistaticity. It can thus be used to quantify yielding behavior of many foams or emulsions but also biological tissues, without the need of explicit force

measurements. Our approach could therefore also prove powerful to study morphogenetic processes that rely on transitions in tissue mechanics.

Future work can further refine the reversible fraction function $f_r(Q_{\mathrm{proj}})$. One extension could account for the observed values of $f_r > 1$ both in experimental and in simulated emulsions. These values likely stem from T1 transitions triggered immediately after the shear reversals. Yet, the trigger for these T1 transitions is possibly different between simulations and experiments. In our vertex model simulations, we prevent T1 transitions going forth and back more often than 3 times during any given quasistatic minimization (see materials and methods). This likely allows some T1 transitions to become "stuck" and occur a little bit later, including upon reversal of the shear direction (Fig. S8 *C*). Another extension could account for nonlocal interactions. Specifically, in some of the data, there were indications of T1 transitions that have been triggered by other T1 transitions through long-range elastic deformations (63). We further considered the system essentially as 1D. Yet, we observed small heterogeneities of the flows with respect to the channel height (see supporting material). In future work, it should be possible to predict the 2D flows using a single 2D constitutive relation akin to $f_r(Q_{\mathrm{proj}})$. Finally, it will be interesting to link our work to related ideas, e.g., studying the density of weak spots in cellular materials including biological tissues (75).

Based on the geometric analysis of the yielding behavior, vertex model simulations helped us test how important the effect of adhesion was on droplet-droplet interface tensions or energy barriers toward droplet rearrangements. We showed that any substantial effect of either would be inconsistent with our experimental observations. While the vertex model actually describes completely dry emulsions, we were able to account for packing fractions lower than 100% by varying the T1 cutoff length. Yet, this approach started to fail at packing fractions below $\sim 98\%$, corresponding to a T1 cutoff of ~ 0.3, essentially, both because of a high droplet area polydispersity, and because the geometry of the continuous-phase triangular regions is completely ignored in the simulations (see materials and methods for details). In the future, smaller packing fractions will be accessed by explicitly accounting for the continuous phase in our simulations (66). Nevertheless, our approach allowed us to show that packing fraction dominated in determining the yielding behavior of our emulsions. In future experiments, we will also vary more broadly the packing fraction and the shear protocol to further test our predictions of the packing-fraction-depending yielding behavior.

Our results also suggest a deviation from the long-standing assumption, initially by Princen (47), that in foams and emulsions, T1 transitions occur when two continuous-phase triangular regions meet. Specifically, we provide direct and indirect evidence that T1 transitions were already triggered much earlier in our emulsions. We are not aware of any previous report of such a deviation from the Princen rule, and, so far, it is unclear what mechanism creates it. For instance, it could be due to either a static instability that leads to an earlier triggering of the T1. Another possibility could be that frictional forces play a role, as previously discussed in foams (76,77). While the large-scale dynamics in our emulsions was quasistatic, friction might still influence the behavior of individual T1 transitions. Regardless of the precise mechanism, while today Princen-like rules are often used when studying cellular materials, e.g., as a criterion for T1 transitions in vertex models, our finding shows that such classical ideas may not be sufficient to describe emulsions, and possibly also biological tissues. It would thus be interesting to explore whether an early triggering of T1s can also be observed in other soft matter and biological systems.

In our experiments, we observed progressive compaction, which only occurred for emulsions with an adhesion differential and only under ongoing cyclic shear deformation. For such progressive compaction to occur, theoretical arguments predict that the flow speed of the continuous phase has to increase in magnitude across subsequent shear cycles (see supporting material). This is confirmed by preliminary data of tracer particles suspended in the continuous phase, which suggest that the continuous phase generally flows faster than the droplet phase (see supporting material, Video S2 and Fig. S15). In other words, the interplay of heterogeneous adhesion and cyclic shear may create an effective pumping of the continuous phase with respect to the droplet phase. However, the precise mechanism driving such a pumping remains to be elucidated.

Several elements of our emulsion findings are reminiscent of features of animal morphogenesis. First, adhesion and compaction play an important role in regulating the fluidity and yielding behavior of biological tissue (4,13,20). Indeed, spatial gradients or temporal increases in compaction have been observed in several developing animals and correlated with corresponding tissue rigidification (4,13,14). Our work relates compaction and tissue rheology, and it provides a new way of quantifying such rigidification from image data alone. Second, cyclic deformations are critical during morphogenetic processes such as heart valve formation (78) or vascular remodeling (79), and axis determination in regenerating *Hydra* (80). Our work shows that such cyclic deformation may under certain conditions create extracellular fluid flows across densely packed cells. More generally, our work highlights how the rheology of a cellular material can be modulated through cellular packing, which is in turn driven by externally applied deformation.

DATA AND CODE AVAILABILITY

Data have been deposited on Zenodo under the https://doi.org/10.5281/zenodo.15585808 and are publicly available as of the article publication date.

ACKNOWLEDGMENTS

We thank Clement Nizak and Raphaël Doineau for their help with the design of the microfluidic chip, as well as Jacques Fattaccioli and Heloise Uhl for the use of the membrane emulsifier. We thank Georges Debregeas, Benjamin Dollet, and Alexandre Kabla for fruitful conversations regarding foam Physics. We also acknowledge funding from EMERGENCE(s) Ville de Paris. Finally, this work was granted access to the HPC resources of the SACADO MeSU platform at Sorbonne Université.

AUTHOR CONTRIBUTIONS

L.-L.P. and Q.G. designed the experiments. Q.G. performed the experiments. Q.G., M.B., and M.M. analyzed the data. M.M., M.B., and R.V. developed the model and performed simulations. Q.G., M.B., R.V., A.M.P., E.W., M.M., and L.-L.P. interpreted the results. M.M. and L.-L.P. wrote the manuscript.

DECLARATION OF INTERESTS

The authors declare no competing interests.

SUPPORTING MATERIAL

Supporting material can be found online at https://doi.org/10.1016/j.bpj.2026.03.004.

REFERENCES

1. Wolpert, L., C. Tickle, and A. M. Arias. 2015. Principles of Development. Oxford University Press.
2. Goodwin, K., and C. M. Nelson. 2021. Mechanics of Development. *Dev. Cell.* 56:240–250. https://pubmed.ncbi.nlm.nih.gov/33321105/.
3. Noll, N., M. Mani, …, B. I. Shraiman. 2017. Active Tension Network Model Suggests an Exotic Mechanical State Realized in Epithelial Tissues. *Nat. Phys.* 13:1221–1226.
4. Mongera, A., P. Rowghanian, …, O. Campàs. 2018. A fluid-to-solid jamming transition underlies vertebrate body axis elongation. *Nature.* 561:401–405. http://www.nature.com/articles/s41586-018-0479-2.
5. Petridou, N. I., and C.-P. Heisenberg. 2019. Tissue rheology in embryonic organization. *EMBO J.* 38:e102497. https://www.embopress.org/doi/abs/10.15252/embj.2019102497.
6. Montel, L., Q. Guigue, and L. L. Pontani. 2022. Adhesion regulation and the control of cellular rearrangements: From emulsions to developing tissues. *Front. Phys.* 10:1014428.
7. Dessalles, C. A., N. Cuny, …, G. Saibreux. 2025. Interplay of actin nematodynamics and anisotropic tension controls endothelial mechanics. *Nat. Phys.* 21:999–1008.
8. Bi, D., J. H. Lopez, …, M. L. Manning. 2015. A Density-Independent Rigidity Transition in Biological Tissues. *Nat. Phys.* 11:1074–1079.
9. Stirbat, T. V., A. Mgharbel, …, H. Delanoë-Ayari. 2013. Fine Tuning of Tissues' Viscosity and Surface Tension through Contractility Suggests a New Role for \alpha-Catenin. *PLoS One.* 8:e52554.
10. Sadeghipour, E., M. A. Garcia, …, B. L. Pruitt. 2018. Shear-induced damped oscillations in an epithelium depend on actomyosin contraction and E-cadherin cell adhesion. *eLife.* 7:e39640.
11. D'Angelo, A., K. Dierkes, …, J. Solon. 2019. In Vivo Force Application Reveals a Fast Tissue Softening and External Friction Increase during Early Embryogenesis. *Curr. Biol.* 29:1564–1571.e6.
12. Khalilgharibi, N., J. Fouchard, …, G. Charras. 2019. Stress relaxation in epithelial monolayers is controlled by the actomyosin cortex. *Nat. Phys.* 15:839–847. https://www.nature.com/articles/s41567-019-0516-6.
13. Petridou, N. I., B. Corominas-Murtra, …, E. Hannezo. 2021. Rigidity percolation uncovers a structural basis for embryonic tissue phase transitions. *Cell.* 184:1914–1928.e19.
14. Michaut, A., A. Mongera, …, O. Pourquié. 2025. Extracellular Volume Expansion Drives Vertebrate Axis Elongation. *Curr. Biol.* 35:843–853.e6.
15. Kashkooli, L., D. Rozema, …, F. Fagotto. 2021. Ectoderm to Mesoderm Transition by Down-Regulation of Actomyosin Contractility. *PLoS Biol.* 19:e3001060.
16. Townes, P. L., and J. Holtfreter. 1955. Directed Movements and Selective Adhesion of Embryonic Amphibian Cells. *J. Exp. Zool.* 128:53–120.
17. Schötz, E.-M., R. D. Burdine, …, -gnskip-->R. a Foty. 2008. Quantitative Differences in Tissue Surface Tension Influence Zebrafish Germ Layer Positioning. *HFSP J.* 2:42–56.
18. Morita, H., S. Grigolon, …, C. P. Heisenberg. 2017. The Physical Basis of Coordinated Tissue Spreading in Zebrafish Gastrulation. *Dev. Cell.* 40:354–366.e4. https://pubmed.ncbi.nlm.nih.gov/28216382/.
19. Wallmeyer, B., S. Trinschek, …, T. Betz. 2018. Collective Cell Migration in Embryogenesis Follows the Laws of Wetting. *Biophys. J.* 114:213–222.
20. Rustarazo-Calvo, L., C. Pallares-Cartes, …, N. I. Petridou. 2025. Adhesion-driven tissue rigidification triggers epithelial cell polarity. *bioRxiv.* https://www.biorxiv.org/content/10.1101/2025.03.18.644006v1https://www.biorxiv.org/content/10.1101/2025.03.18.644006v1.abstract.
21. Gsell, S., S. Tlili, …, P.-F. Lenne. 2025. Marangoni-like tissue flows enhance symmetry breaking of embryonic organoids. *Nat. Phys.* 21:644–653.
22. Cachat, E., W. Liu, …, J. A. Davies. 2016. 2- and 3-Dimensional Synthetic Large-Scale de Novo Patterning by Mammalian Cells through Phase Separation. *Sci. Rep.* 6:20664.
23. Courte, J., C. Chung, …, L. Morsut. 2024. Programming the elongation of mammalian cell aggregates with synthetic gene circuits. *Preprint at bioRxiv* 2024.12.11.627621. https://doi.org/10.1101/2024.12.11.627621.
24. Steinberg, M. S. 1963. Reconstruction of Tissues by Dissociated Cells. *Science.* 141:401–408.
25. Foty, R. a., and M. S. Steinberg. 2005. The differential adhesion hypothesis: a direct evaluation. *Dev. Biol.* 278:255–263. http://www.ncbi.nlm.nih.gov/pubmed/15649477.
26. Brodland, G. W. 2002. The Differential Interfacial Tension Hypothesis (DITH): A Comprehensive Theory for the Self-Rearrangement of Embryonic Cells and Tissues. *J. Biomech. Eng.* 124:188–197.
27. Manning, M. L., R. a. Foty, …, E.-M. Schoetz. 2010. Coaction of intercellular adhesion and cortical tension specifies tissue surface tension. *Proc. Natl. Acad. Sci. USA.* 107:12517–12522. http://www.pubmedcentral.nih.gov/articlerender.fcgi?artid=2906578&tool=pmcentrez&rendertype=abstract.
28. Maître, J. L., H. Berthoumieux, …, C.-P. Heisenberg. 2012. Adhesion Functions in Cell Sorting by Mechanically Coupling the Cortices of Adhering Cells. *Science.* 338:253–256.
29. Amack, J. D., and M. L. Manning. 2012. Knowing the Boundaries : Extending the Differential Adhesion Hypothesis. *Science.* 338:212–215.
30. Graner, F., and J. A. Glazier. 1992. Simulation of Biological Cell Sorting Using a Two-Dimensional Extended Potts Model. *Phys. Rev. Lett.* 69:2013–2016.
31. Gsell, S., and M. Merkel. 2022. Phase Separation Dynamics in Deformable Droplets. *Soft Matter.* 18:2672–2683.
32. Pawlizak, S., A. W. Fritsch, …, J. A. Käs. 2015. Testing the Differential Adhesion Hypothesis across the Epithelial-mesenchymal Transition. *New J. Phys.* 17:083049.
33. Erdemci-Tandogan, G., and M. L. Manning. 2021. Effect of Cellular Rearrangement Time Delays on the Rheology of Vertex Models for Confluent Tissues. *PLoS Comput. Biol.* 17:e1009049.
34. Pontani, L. L., I. Jorjadze, …, J. Brujic. 2012. Biomimetic emulsions reveal the effect of mechanical forces on cell-cell adhesion. *Proc.*

Natl. Acad. Sci. USA. 109:9839–9844. http://www.pnas.org/cgi/doi/10.1073/pnas.1201499109.

35. Golovkova, I., L. Montel, …, L. L. Pontani. 2020. Depletion attraction impairs the plasticity of emulsions flowing in a constriction. *Soft Matter.* 16:3294–3302. https://pubs.rsc.org/en/content/articlehtml/2020/sm/c9sm02343g.

36. Golovkova, I., L. Montel, …, L.-L. Pontani. 2021. Adhesion as a trigger of droplet polarization in flowing emulsions. *Soft Matter.* http://xlink.rsc.org/?DOI=D1SM00097G.

37. Montel, L., I. Golovkova, …, L. L. Pontani. 2021. Adhesion Percolation Determines Global Deformation Behavior in Biomimetic Emulsions. *Front. Phys.* 9:744006.

38. Dahmann, C., A. C. Oates, and M. Brand. 2010. Boundary formation and maintenance in tissue development. *Nat. Rev. Genet.* 12:43–55. https://www.nature.com/articles/nrg2902.

39. Togashi, H. 2016. Differential and cooperative cell adhesion regulates cellular pattern in sensory epithelia. *Front. Cell Dev. Biol.* 4:104. www.frontiersin.org.

40. Nagendra, K., A. Izzet, …, J. Brujic. 2023. Push-pull mechanics of E-cadherin ectodomains in biomimetic adhesions. *Biophys. J.*. https://pubmed.ncbi.nlm.nih.gov/37528581/.

41. Merkel, M., R. Etournay, …, F. Jülicher. 2017. Triangles bridge the scales: Quantifying cellular contributions to tissue deformation. *Phys. Rev. E.* 95:032401. https://journals.aps.org/pre/abstract/10.1103/PhysRevE.95.032401.

42. Dumortier, J. G., M. Le Verge-Serandour, …, J.-L. Maître. 2019. Hydraulic Fracturing and Active Coarsening Position the Lumen of the Mouse Blastocyst. *Science.* 365:465–468.

43. Zhang, Y., A. McMullen, …, P. M. Chaikin. 2017. Sequential self-assembly of DNA functionalized droplets. *Nat. Commun.* 8:21. http://www.nature.com/articles/s41467-017-00070-0.

44. Pinon, L., L. Montel, …, J. Fattaccioli. 2018. Kinetically Enhanced Fabrication of Homogeneous Biomimetic and Functional Emulsion Droplets. *Langmuir.* 34:15319–15326. http://pubs.acs.org/doi/10.1021/acs.langmuir.8b02721.

45. Bourouina, N., J. Husson, …, D. R. Wilson. 2011. Formation of specific receptor–ligand bonds between liquid interfaces. *Soft Matter.* 7:9130. http://xlink.rsc.org/?DOI=c1sm05659j.

46. Berg, S., D. Kutra, …, A. Kreshuk. 2019. Ilastik: Interactive Machine Learning for (Bio)Image Analysis. *Nat. Methods.* 16:1226–1232.

47. Princen, H. M. 1983. Rheology of foams and highly concentrated emulsions. *J. Colloid Interface Sci.* 91:160–175. http://www.sciencedirect.com/science/article/pii/0021979783903235.

48. Feng, L., L.-L. Pontani, …, J. Brujic. 2013. Specificity, flexibility and valence of DNA bonds guide emulsion architecture. *Soft Matter.* 9:9816. http://xlink.rsc.org/?DOI=c3sm51586a.

49. Pontani, L.-L., I. Jorjadze, and J. Brujic. 2016. Cis and Trans Cooperativity of E-Cadherin Mediates Adhesion in Biomimetic Lipid Droplets. *Biophys. J.* 110:391–399. http://www.ncbi.nlm.nih.gov/pubmed/26789762.

50. SantaLucia, J., O. Theodoly, …, P. M. Chaikin. 1998. A unified view of polymer, dumbbell, and oligonucleotide DNA nearest-neighbor thermodynamics. *Proc. Natl. Acad. Sci. USA*. 95:1460–1465. http://www.ncbi.nlm.nih.gov/pubmed/9465037http://www.pubmedcentral.nih.gov/articlerender.fcgi?artid=PMC19045.

51. Etournay, R., M. Popović, …, S. Eaton. 2015. Interplay of cell dynamics and epithelial tension during morphogenesis of the Drosophila pupal wing. *eLife.* 4:e07090.

52. Kong, D., F. Wolf, and J. Großhans. 2017. Forces Directing Germ-Band Extension in Drosophila Embryos. *Mech. Dev.* 144:11–22.

53. Graner, F., B. Dollet, …, P. Marmottant. 2008. Discrete rearranging disordered patterns, part I: Robust statistical tools in two or three dimensions. *Eur. Phys. J. E Soft Matter.* 25:349–369. https://link.springer.com/article/10.1140/epje/i2007-10298-8.

54. Dollet, B., and C. Bocher. 2015. Flow of foam through a convergent channel. *Eur. Phys. J. E Soft Matter.* 38:123. https://link.springer.com/article/10.1140/epje/i2015-15123-3.

55. Blanchard, G. B., A. J. Kabla, …, R. J. Adams. 2009. Tissue tectonics: morphogenetic strain rates, cell shape change and intercalation. *Nat. Methods.* 6:458–464. http://www.ncbi.nlm.nih.gov/pubmed/19412170.

56. Guirao, B., S. U. Rigaud, …, Y. Bellaïche. 2015. Unified Quantitative Characterization of Epithelial Tissue Development. *eLife.* 4:e08519.

57. Tlili, S., M. Durande, …, H. Delanoë-Ayari. 2020. Migrating Epithelial Monolayer Flows like a Maxwell Viscoelastic Liquid. *Phys. Rev. Lett.* 125:088102.

58. Butler, L. C., G. B. Blanchard, …, B. Sanson. 2009. Cell shape changes indicate a role for extrinsic tensile forces in Drosophila germ-band extension. *Nat. Cell Biol.* 11:859–864. https://pubmed.ncbi.nlm.nih.gov/19503074/.

59. Dye, N. A., M. Popović, …, F. Jülicher. 2021. Self-Organized Patterning of Cell Morphology via Mechanosensitive Feedback. *eLife.* 10:e57964.

60. Batchelor, G. K. 1970. The Stress System in a Suspension of Force-Free Particles. *J. Fluid Mech.* 41:545–570.

61. Kraynik, A. M., D. A. Reinelt, and F. Van Swol. 2003. Structure of Random Monodisperse Foam. *Phys. Rev.* 67:031403.

62. Kabla, A., and G. Debregeas. 2007. Quasi-Static Rheology of Foams. Part 1. Oscillating Strain. *J. Fluid Mech.* 587:23–44.

63. Nicolas, A., E. E. Ferrero, …, J.-L. Barrat. 2018. Deformation and Flow of Amorphous Solids: Insights from Elastoplastic Models. *Rev. Mod. Phys.* 90:045006.

64. Farhadifar, R., J. C. Röper, …, F. Jülicher. 2007. The Influence of Cell Mechanics, Cell-Cell Interactions, and Proliferation on Epithelial Packing. *Curr. Biol.* 17:2095–2104.

65. Alt, S., P. Ganguly, and G. Salbreux. 2017. Vertex Models: From Cell Mechanics to Tissue Morphogenesis. *Philos. Trans. R. Soc. Lond. B Biol. Sci.* 372:20150520.

66. Kim, S., M. Pochitaloff, …, O. Campàs. 2021. Embryonic Tissues as Active Foams. *Nat. Phys.* 17:859–866.

67. Chen, Y., Q. Gao, …, H. Jiang. 2022. Activation of Topological Defects Induces a Brittle-to-Ductile Transition in Epithelial Monolayers. *Phys. Rev. Lett.* 128:018101. https://journals.aps.org/prl/abstract/10.1103/PhysRevLett.128.018101.

68. Li, C. H., X. Yin, …, G. K. Xu. 2025. Time-dependent active force drives periodic reversal in collective cell migration. *Biophys. J.* 124:3542–3554. https://www.sciencedirect.com/science/article/pii/S0006349525005235.

69. Karnat, M., G. H. Narayana, …, J. F. Rupprecht. 2025. Noninvasive rheological inference from stable flows in confined tissues. *Preprint at arXiv* arXiv:2511.20155.

70. Raufaste, C., B. Dollet, …, F. Graner. 2007. Yield drag in a two-dimensional foam flow around a circular obstacle: Effect of liquid fraction. *Eur. Phys. J. E Soft Matter.* 23:217–228.

71. Forterre, Y., and O. Pouliquen. 2008. Flows of Dense Granular Media. *Annu. Rev. Fluid Mech.* 40:1–24.

72. Liu, A. J., and S. R. Nagel. 2010. The Jamming Transition and the Marginally Jammed Solid. *Annu. Rev. Condens. Matter Phys.* 1:347–369.

73. Das, R., S. Sinha, …, D. Thirumalai. 2024. Free volume theory explains the unusual behavior of viscosity in a non-confluent tissue during morphogenesis. *eLife.* 12. https://elifesciences.org/reviewed-preprints/87966.

74. Weaire, D., and S. Hutzler. 1999. The Physics of Foams. Clarendon Press.

75. Popović, M., V. Druelle, …, M. Wyart. 2021. Inferring the Flow Properties of Epithelial Tissues from Their Geometry. *New J. Phys.* 23:033004.

76. Durand, M., and H. A. Stone. 2006. Relaxation time of the topological T1 process in a two-dimensional foam. *Phys. Rev. Lett.* 97:226101. https://journals.aps.org/prl/abstract/10.1103/PhysRevLett.97.226101.

77. Biance, A. L., S. Cohen-Addad, and R. Höhler. 2009. Topological transition dynamics in a strained bubble cluster. *Soft Matter.* 5:4672–4679. https://pubs.rsc.org/en/content/articlehtml/2009/sm/b910150k.

78. Vermot, J., A. S. Forouhar, …, S. E. Fraser. 2009. Reversing Blood Flows Act through klf2a to Ensure Normal Valvulogenesis in the Developing Heart. *PLoS Biol.* 7:e1000246. https://journals.plos.org/plosbiology/article?id=10.1371/journal.pbio.1000246.

79. Lucitti, J. L., E. A. V. Jones, …, M. E. Dickinson. 2007. Vascular remodeling of the mouse yolk sac requires hemodynamic force. *Development (Cambridge, England).* 134:3317–3326. https://pubmed.ncbi.nlm.nih.gov/17720695/.

80. Ferenc, J., P. Papasaikas, …, C. D. Tsiairis. 2021. Mechanical oscillations orchestrate axial patterning through Wnt activation in Hydra. *Sci. Adv.* 7:6897. https://doi.org/10.1126/sciadv.abj6897?download=true.

Biophysical Journal, Volume *125*

Supplemental information

Adhesion differentials control the rheology of biomimetic emulsions

Quentin Guigue, Marc Besse, Raphael Voituriez, Alexis M. Prevost, Elie Wandersman, Matthias Merkel, and Lea-Laetitia Pontani

Supplementary information:

Adhesion differentials control the rheology of biomimetic emulsions

Quentin Guigue, Marc Besse, Raphael Voituriez, Alexis M. Prevost, Elie Wandersman, Matthias Merkel[†], Lea-Laetitia Pontani[†]

[†] Corresponding authors:

lea-laetitia.pontani@sorbonne-universite.fr
matthias.merkel@univ-amu.fr

This PDF file includes:

- Supplementary text
- Figures S1 to S15
- SI References

Contents

1 DNA sequences **3**

2 Experimental set-up **3**

3 Straight channel geometry **4**

4 Image analysis **4**

5 P14 sequences **6**

6 Red/Green droplet proportion across conditions and unmixing **6**

7 Quasi-staticity **7**

8 Droplet shape and shear decomposition **7**
- 8.1 Triangle-based quantities . . . 7
- 8.2 Locally averaged quantities . . . 8
- 8.3 Quantification of droplet shape variation . . . 9
- 8.4 Fitting of Q_* from experimental f_r curves . . . 9
- 8.5 Beyond a Q_{proj}-dependent f_r . . . 9

9 Quantification of the flow velocity for the prediction of $Q_{xx}(x)$ **11**

10 Packing fraction evolution **12**

11 Estimation of interface tension ratio from contact angles at triple junctions **12**

12 Estimation of the parameter α **16**

13 A gradient in packing fraction suggests pumping **16**
- 13.1 Hypothesis . . . 16
- 13.2 Flow velocity measurements . . . 16

14 Supplementary Videos **18**
- 14.1 Movie S1: flow of an heterogeneous emulsion . . . 18
- 14.2 Movie S2: flow of an heterogeneous emulsion with fluorescent tracers in the continuous phase . . 18

1 DNA sequences

The DNA sequences presented below share the same structure : a common backbone spacer sequence of 49 bp and a specific sticky end of various length (underlined part in the sequences) separated by a single non-hybridizable base, serving as a flexible junction. A biotin group for grafting of the sequence onto the lipids is ligated to the 3' end of the sequences through the intermediary of a triethylene glycol spacer. Sequences are named after the number of base pairs constitutive of the sticky end, varying between 0 bp and 14 bp.

A complementary sequence (CS) to the backbone is hybridized to these sequences before their grafting onto the droplets. This stiffens the spacer to the sticky and favors adhesion [3, 2].

Palindromic DNA sequences

P0: 5'-G CAT TAC TTT CCG TCC CGA GAG ACC TAA CTG ACA CGC TTC CCA TCG CTA[BtnTg]-3'

P6: 5'- <u>TAC GTA</u> A G CAT TAC TTT CCG TCC CGA GAG ACC TAA CTG ACA CGC TTC CCA TCG CTA[BtnTg]-3'

P10: 5'-<u>AAT CAT GAT T</u> A G CAT TAC TTT CCG TCC CGA GAG ACC TAA CTG ACA CGC TTC CCA TCG CTA[BtnTg]-3'

P14: 5'-<u>TAT GCA TAT GCA TA</u> A G CAT TAC TTT CCG TCC CGA GAG ACC TAA CTG ACA CGC TTC CCA TCG CTA[BtnTg]-3'

Complementary sequence

CS: 5'-TAG CGA TGG GAA GCG TGT CAG TTA GGT CTC TCG GGA CGG AAA GTA ATG C-3'

2 Experimental set-up

Once the emulsion is prepared, the emulsion mix is left to cream, in a tube with pressure control, and in a water bath at ≈ 40°C to avoid any preliminary interaction between the droplets. The emulsion is then injected into the passivated microchannel through a Tygon connected to a pressure pump (MFCS-8C Fluigent), at a typical pressure of ∼20 mBar. We let the emulsion flow in the channel until a high packing fraction is reached (note that imposed pressure can be modulated to help the filling and packing processes). The outlet and drain of the channel are both connected to a waste collection Eppendorf.

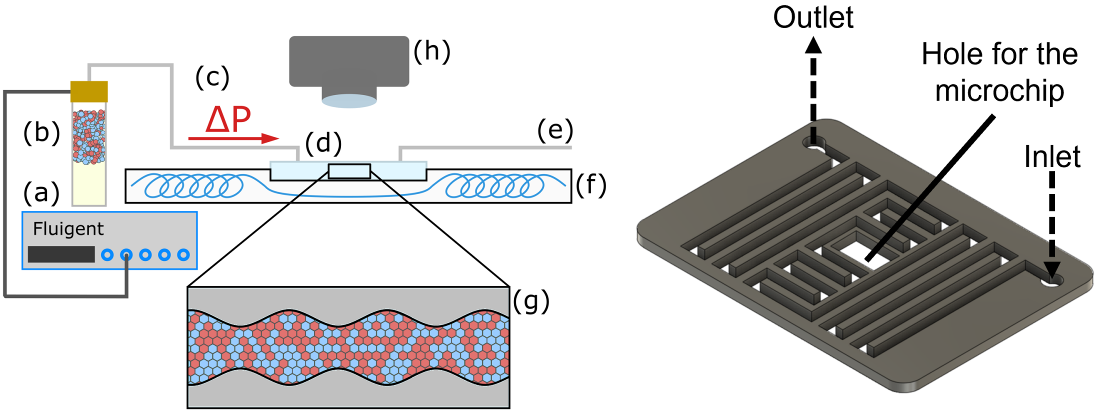

Figure 1: (Left) Microfluidic setup. A pressure controller (a) is connected to a reservoir containing the creamed emulsion (b). We impose a pressure (c) to push the emulsion from the reservoir to the microchip (d) set on a cooling stage (f). The emulsion flow in the microchip undergoes oscillatory shear due to the design of the chip (g). Waste is collected at the outlet of the chip (e). Images are acquired using spinning disk confocal microscopy (h). (Right) Schematic of the custom cooling stage.

Due to the sensibility of DNA hybridization to temperature, the microfluidic chip is mounted on a custom cooling microscope stage. It consists of three PMMA plates assembled together with optical glue. The middle plate contains channels, which allow us to circulate cold water through the stage. All three plates of the stage have a 15×25 mm rectangular hole in the middle to accommodate for the micro-chip and its tubing during the experiment. The custom stage is connected to a CC-K6 Cooling bath thermostat (Huber). Cold water is therefore circulated into the microscope stage which cools down the micro-chip by conduction. This allows us to maintain the chip temperature at ≈ 18°C, ensuring proper adhesion of all DNA sequences.

3 Straight channel geometry

The design of the straight channel is overall similar to the undulated one, except that the oscillations were removed. In order to find the equivalent position between the two channels, we added some marks along the straight channel that are separated by 420µm, the periodicity of the undulated channel.

Note that the constriction at the exit of the channel is still present to help for the packing of the droplets. As this constriction inherently applies a mechanical perturbation on the droplets, no acquisition was proceeded in this position.

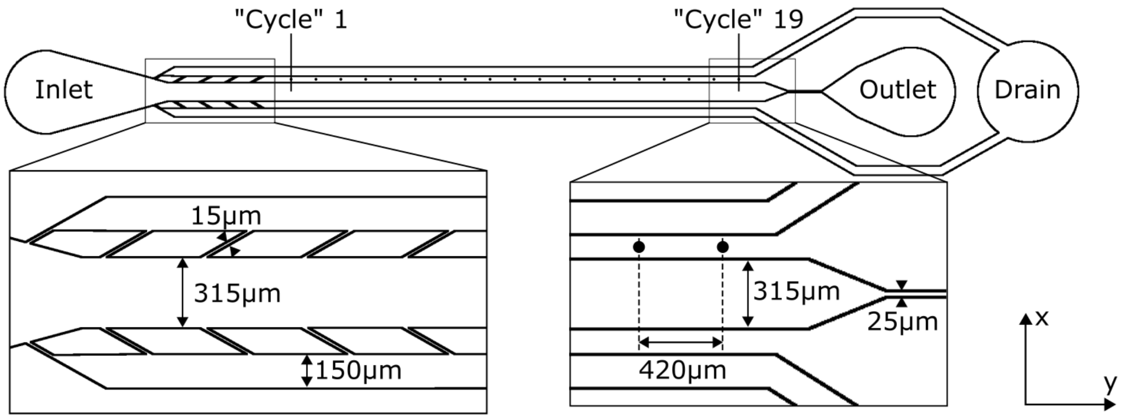

Figure 2: Schematic and dimensions of the straight channel used for control experiments.

4 Image analysis

Fluorescence channels are initially analyzed separately. In Figure 3A, we show the two superimposed channels of fluorescence for better visualization. A typical static experiment corresponds to 4 to 10 acquisitions of the twenty positions of the channel (nineteen in the case of the straight channel; an example of a raw image is given in Figure 3A). For each experiment and each fluorescence channel, we therefore construct a sample consisting of 2 images per acquisition of the full channel. This sample is then used to classify pixels between foreground (droplets contour) and background using a Random Forest Algorithm with Ilastik [1] parameterized with the following features: Color/Intensity 3.5-5-10 σ, Edge 3.5-5 σ, Texture 3.5-5 σ. The algorithm is manually trained until the classifier is able to correctly predict the contour of the droplets. A manual check is performed for each image in order to limit detection errors. An example of the resulting segmentation is shown in Figure 3B. A custom Fiji routine is then used to identify the droplets out of the resulting binary images. In particular, we use the Analyze Particles module to detect objects that are larger than 1500 pixels^2 ($\simeq 76\mu m^2$) and with a circularity above 0.70.

Once the process is completed for both channels, the obtained binarized masks are summed to recover a complete picture of the image and a surface Voronoi tessellation is produced using Fiji as shown in Figure 3C. This tessellation will later allow us to select the droplets that will be use or not in the upcoming analysis, identify the neighborhood of each droplet and evaluate their local packing fraction. Next, we use the Python Sci-kit image library to geometrically characterize each droplet. We focus on the droplets whose corresponding Voronoi cell are not touching neither the left/right borders of the image (as they correspond to cropped droplet in between two waves) nor the top/bottom borders (corresponding to outer droplets touching the border of the channel and therefore inherently having a different neighborhood). An example of those selected droplets is shown by yellow colored cells on Figure 3C.

For each remaining droplet we use the regionprops function to measure its position, orientation, area and perimeter. Position and orientation are obtained by fitting the droplets with an ellipse that has the same second moment (resulting orientation and example of fitted ellipse are shown in Figure 3D-E). The area is evaluated by pixel counting and droplets with an area larger than 10^5 pixels$^2 \simeq 5000\,\mu m^2$ are excluded. The perimeter is approached by the length of a line fitting the border pixels using a 4-connectivity, an example of those identified contours is shown in Figure 3F-G.

The area a is then used together with the area of the corresponding Voronoi cell to compute a local packing fraction as $\phi_{\text{loc}} = a/a_v$, a_v being the area of the Voronoi cell. Droplets with a local packing fraction lower than 0.94 are discarded in further quantification.

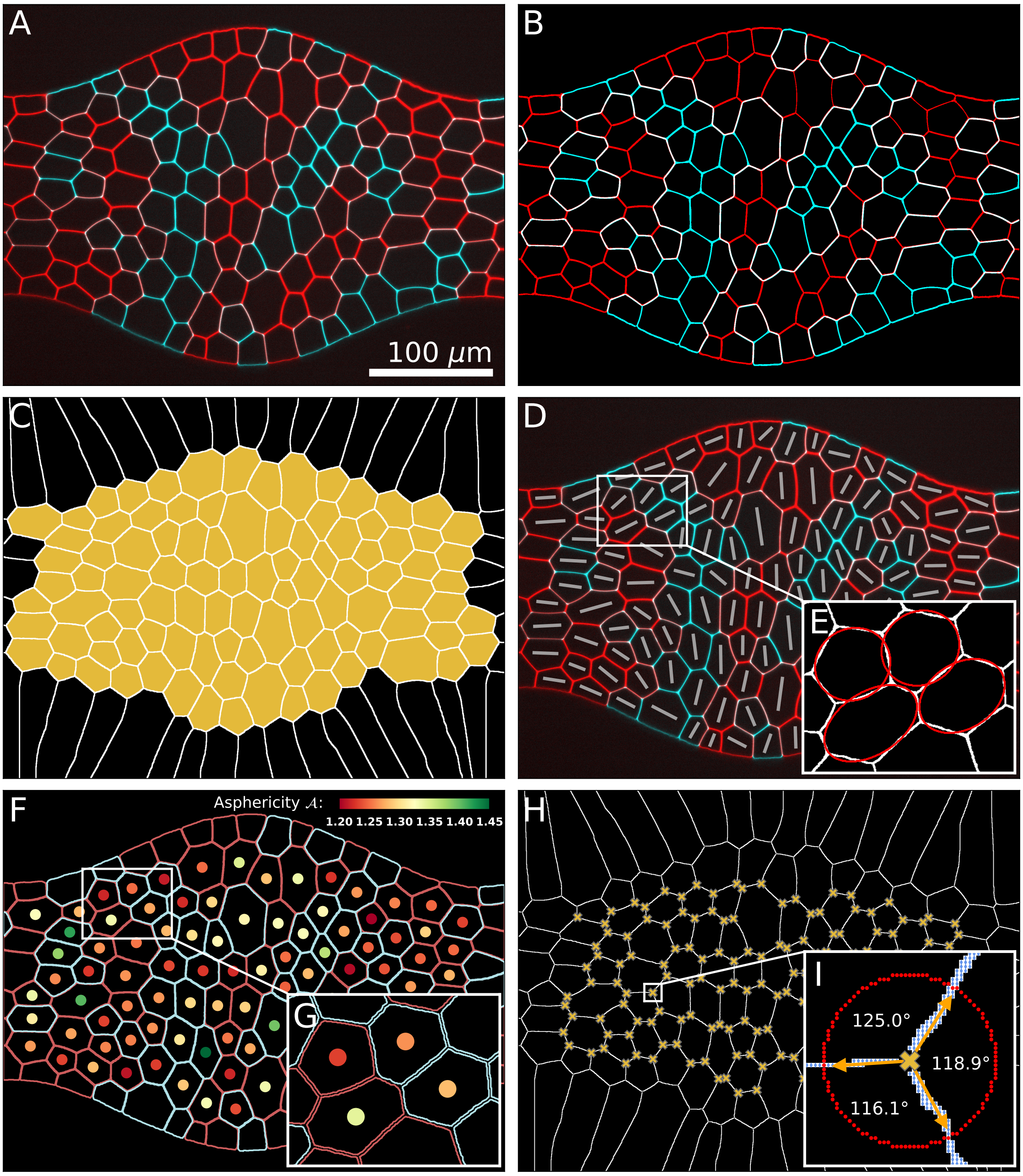

Figure 3: (A) Confocal image of two populations of droplets grafted with palindromic sequences. Red-red droplets interact through 10bp, blue-blue droplets interact through 0bp, i.e. they do not interact through specific adhesion, and similarly, red-blue droplets do not interact. (B) Result of the segmentation after training on Ilastik. (C) A surface Voronoi tessellation is performed in order to exclude droplets on the edge of the channel and select the inner droplets (here highlighted in yellow). (D-E) Droplets are fitted by ellipses (E) in order to extract their position and orientation (grey segments) with respect to the horizontal axis (D). (F-G) The area a of each droplet is approximated through pixel counting while the perimeter p is approximated by the length of a line fitting the pixels of the contour (red and blue lines). Based on this information, we compute the shape factor as $\mathcal{A} = p^2/4\pi a$ (colored dots). (H-I) Edges and vertex of the network can be identified separately. Each inner vertex (yellow cross, vertex constituted exclusively by inner droplets identified in (C)) is associated to its contributing edges from which we identify the point coordinates (blue dots) that we use to fit a line and find the direction vector (orange arrow) to further quantify the pairwise angles between edges around the vertex (I). Inset (I) shows an example of the resulting quantification.

A shape factor index is also computed for each droplet as $\mathcal{A} = p^2/4\pi a$, with p the perimeter of the droplet. This shape factor represents the deviation of a given shape compared to a perfect disk which would yield $\mathcal{A} = 1$, the higher the deformation the higher $\mathcal{A}$ is. Colored dots on Figure 3F-G represent the intensity of the measured shape factor of the associated droplet.

Using the Voronoi tessellation Figure 3C, we are able to separate vertex and edges of the network Figure 3H (yellow crosses and white lines respectively) and keep only inner vertices, i.e. vertices formed exclusively by inner droplets. Constitutive edges of the vertex are identified by drawing a small circle around its center (red dotted circle in Figure 3I). We focused only on cases where the vertex is formed by exactly three droplets. Each vertex is then broken down into pixel-points coordinates (blue dots in Figure 3I) that are fitted by a straight line, allowing us to approximate a direction vector (orange arrows in Figure 3I). Once the three vectors of the vertex are computed, we pair-wisely quantify the angles between the three edges of the vertex and repeat for each vertex of the image.

5 P14 sequences

Similarly to the results presented in Fig. 2A, we imaged emulsions made with P0/P14 (pink crosses) and P6/P14 (purple diamonds) mixtures as they progress inside the channel cycles. Compared to the PO/P10 and P6/P10 experiments, these conditions are thus associated with a higher average binding energy, which in turn leads to a higher deformation at the beginning of the channel (see Figure 4-Left for shape factor and Figure 4-Right for ΔQ_{xx} measurements). In addition to this, the highest adhesion differential, namely P0P14, also leads to a larger increase in deformation across cycles.

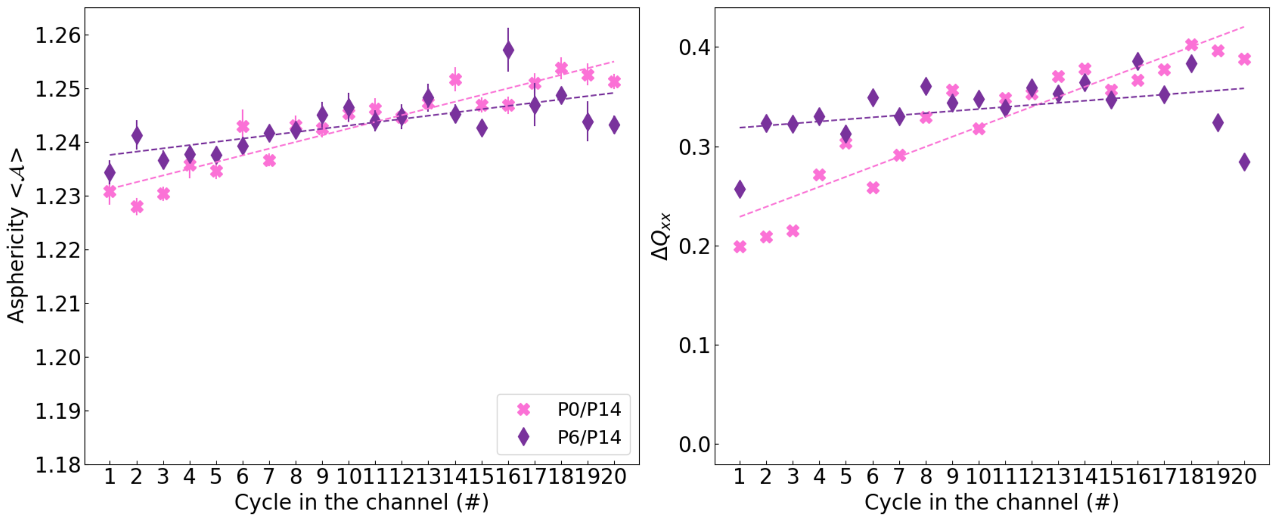

Figure 4: Deformation of droplets in P0/P14 (pink crosses) and P6/P14 (purple diamonds) emulsions. (Left) Average asphericity $\langle\mathcal{A}\rangle$ computed over all the droplets in each undulation cycle for both conditions. The analysis was performed on static images acquired after the flow was arrested in the channel. Error bars represent the standard error of the mean across experimental repetitions. (Right) Evolution of ΔQ_{xx}, calculated as the amplitude of $Q_{xx}(x)$ variations within a channel oscillation, as a function of the undulation.

6 Red/Green droplet proportion across conditions and unmixing

The proportion of droplets from each population remains stable over the successive shear cycles (Figure 5 Left), indicating an absence of emulsion sorting along the channel. A slight deviation from a 1:1 proportion of each population appears only when the most adhesive droplets P14 are involved but still no sorting of the emulsion along the channel is observed in that case. Moreover, these emulsions are not used for most of the characterization and modelling in the main text.

In addition, we quantified the mixing in heterogeneous emulsions as the ratio between the total heterotypic interface length (i.e. red/blue contacts) and the total interface length (red/blue, blue/blue and red/red contacts), along the oscillatory channel's positions (Figure 5 Right). This analysis reveals that the mixing remains stable across shear cycles.

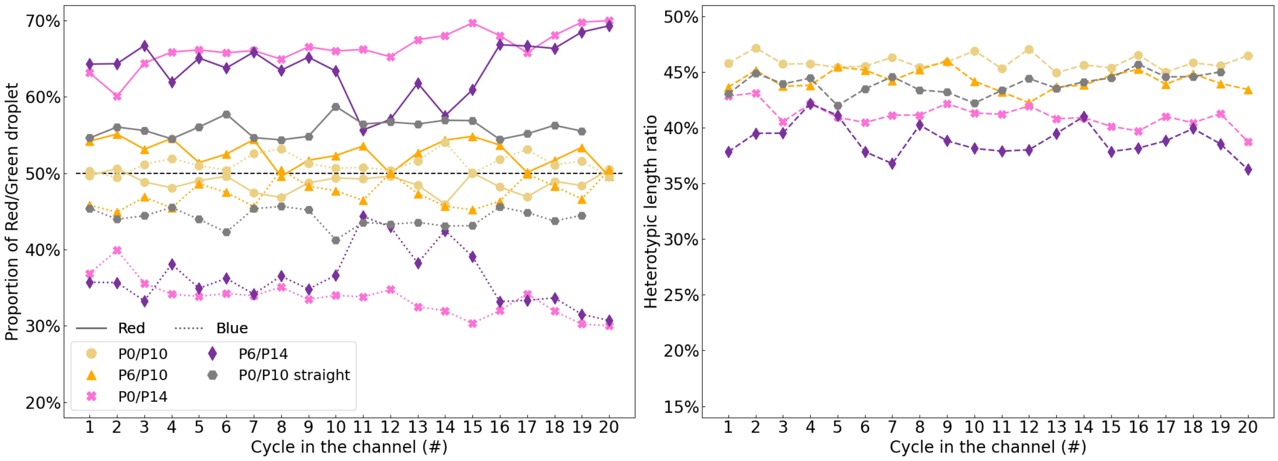

Figure 5: (Left) Proportion of red (solid lines) and blue (doted lines) droplets across channel positions averaged over all heterogeneous emulsions used in static acquisitions. (Right) Heterotypic length ratio, i.e. total heterotypic interface length (red/blue contacts) divided by the total interface length, as a function of the position in the channel averaged over all heterogeneous emulsions used in static acquisitions.

7 Quasi-staticity

T1 transitions and the associated elastic relaxation typically take place on a time scale below the image acquisition time interval of $\Delta t = 5 \times 10^{-2}$ s. This is much faster than the applied deformation: the fastest flow speed of 50 µm/s corresponds to a travel time of 8.4 s for any droplets across a single shear cycle. Therefore, T1 events and elastic relaxation occur on much shorter timescales than the shear timescale, which is defined by the channel geometry.

In addition, we examined how droplet asphericity ($\mathcal{A}$) and anisotropy (Q_{xx}) depend on flow velocity. Figure 6 displays the results for P10 homogeneous emulsions. Both the asphericity (Figure 6 Left) and the droplet anisotropy (Figure 6 Right) are independent of the average flow speed.

8 Droplet shape and shear decomposition

8.1 Triangle-based quantities

We follow Ref. [5] in quantifying triangle and droplet shape. Briefly, in a first step, we triangulate the segmented droplet image. To this end, we take any vertex (or triangular aqueous region). If the three adjacent droplets are fully visible, we define a triangle by connecting the three barycenters of the droplets. If more than three droplets meet at an aqueous region, we proceed as for many-fold vertices in Ref. [5]. Briefly, in this case, we compute the average $\boldsymbol{c}$ of the barycenters of all adjacent droplets, and then define triangles between two adjacent droplet centers and $\boldsymbol{c}$, respectively.

For each triangle n, we then compute a shear rate tensor $\tilde{V}^n_{ij}$ and a shape anisotropy tensor Q^n_{ij} in the following way. Briefly, to compute the shear rate tensor for some time step $t \to t' = t + \delta t$, we consider the affine transformation matrix M^n_{ij} required to transform triangle n at time t into triangle n at time t'. Then, the triangle-based velocity gradient is computed as $V^n_{ij} = (M^n_{ji} - \delta_{ij})\delta t$. Finally, the shear rate tensor $\tilde{V}^n_{ij}$ is computed as the symmetric, traceless part of V^n_{ij}.

To compute the shape tensor Q^n_{ij} of any triangle at any time point t, we proceed again following Ref. [5]. Briefly, we first consider the affine transformation matrix $\boldsymbol{S}^n$ that transforms an equilateral reference triangle into triangle n at time t. The matrix $\boldsymbol{S}^n$ can be expressed as a tensor product:

$$\boldsymbol{S}^n = \left(\frac{a^n}{a_0}\right)^{1/2} \exp(\boldsymbol{Q}^n) \cdot \mathbf{R}(\theta^n). \tag{1}$$

The first term represents an isotropic scaling, where a^n and a_0 are the area of the triangle and the reference triangle, respectively. The second term is a shear transformation parameterized by the triangle shape anisotropy tensor Q^n_{ij}, which is symmetric and traceless. θ^n is an absolute rotation angle of the triangle, where $\mathbf{R}(\theta^n)$ denotes a counter-clockwise rotation by θ^n. Note that the orientation angle of Q^n_{ij} and θ^n two different and completely independent angles. The product decomposition in (1) is unique. In particular, it uniquely defines

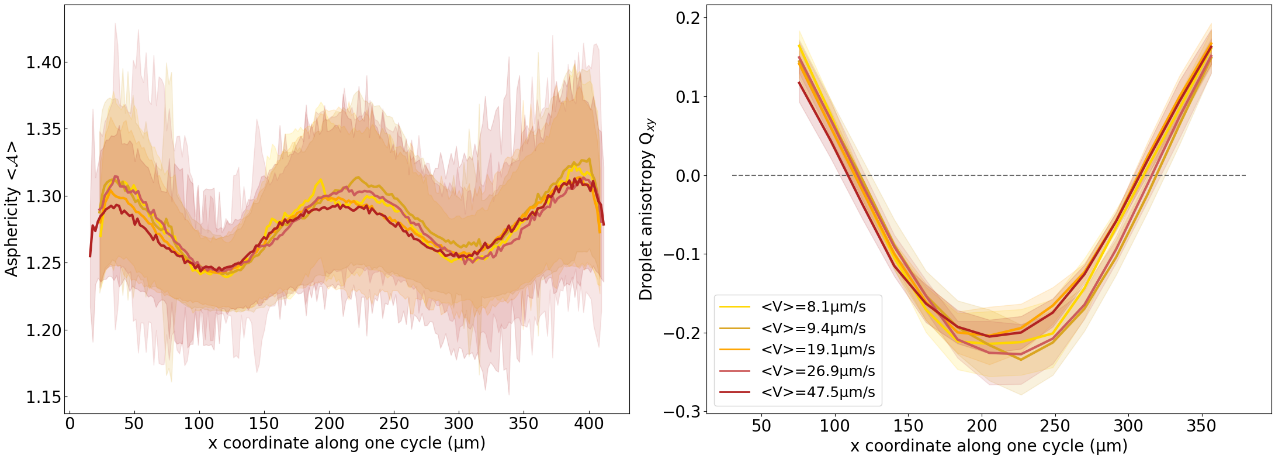

Figure 6: Droplet shape properties over one shear cycle in the channel for different flow velocities, here shown for P10 homogeneous emulsion in the fifth position of the undulated channel. (Left) Shape factor $\mathcal{A} = p^2/4\pi a$ as a function of the position. (Right) Droplet anisotropy Q_{xx} as a function of the position (see section 8). For both $\mathcal{A}$ and Q_{xx}, shaded areas represent the standard deviation. Both plots indicate that droplet shape is independent of flow velocity.

Q^n_{ij} [5]. Furthermore, one can show that its magnitude, $Q = \sqrt{Q^2_{xx} + Q^2_{xy}}$, corresponds to $\log(\mathrm{AR})/2$, where AR is the aspect ratio of the uniquely defined ellipse whose perimeter goes through all three triangle points and whose barycenter coincides with the triangle barycenter [5].

8.2 Locally averaged quantities

To obtain locally averaged quantities for droplet shape anisotropy Q_{xx}, shear rate $\tilde{V}_{xx}$, and the reversible fraction f_r, we first divide our images into a grid of $a^b_0 = 96 \times 96\,\mathrm{pixel}^2$ (i.e. $a^b_0 = 21.5\,\mu\mathrm{m} \times 21.5\,\mu\mathrm{m}$) boxes, which we label here by their center positions $b = (x, y)$. We next compute the intersection of each box b with each triangle n, which respectively results in a polygon of area $a^{n\cap b}$. Clearly, the sum of these polygon areas over all boxes corresponds to the corresponding triangle area $\sum_b a^{n\cap b} = a^n$. Conversely, the sum over all polygon areas over all triangles is $\sum_n a^{n\cap b} = a^b$, which can be smaller than the full box area a^b_0, since some boxes (i.e. those at the at the image boundaries) are not entirely covered by triangles. Yet, in all our quantifications, we only took boxes into account that, at the time point of interest, are entirely covered by triangles, i.e. those boxes for which $a^b = a^b_0$.

Then, the average droplet shape anisotropy for any given box b is given by the area-weighted average of the shape tensors of the covering triangles:

$$Q^b_{ij} = \frac{1}{a^b} \sum_n a^{n\cap b} Q^n_{ij} \tag{2}$$

with $i, j \in \{x, y\}$. Similarly, the average shear rate of a given box b is given by:

$$\tilde{V}^b_{ij} = \frac{1}{a^b} \sum_n a^{n\cap b} \tilde{V}^n_{ij}. \tag{3}$$

To be able to compute the reversible fraction, we also need to quantify the droplet shape derivative $\mathrm{d}\boldsymbol{Q}/\mathrm{d}t = \partial\boldsymbol{Q}/\partial t + \mathrm{v}_i \partial_i \boldsymbol{Q}$ for a given box for some time interval $t \to t' = t + \delta t$. The partial derivative part is computed using (2) at two subsequent time points, and dividing their difference by δt. Yet, we observe that this contribution is negligible as compared to the advective term (stationary state; see Figure 8A). To compute the advective term, we consider for each triangle n at time t a modified version n' at time t'. We therefore remember that n was created by placing its corners onto the barycenters of three droplets. We then construct n' from the barycenters of these droplets *at time* t'. Note that n' is not necessarily in the usual triangulation at n', since the three droplets may not necessarily be neighbors at time t' any more. Then, the advective term for box b is computed as

$$\Delta Q^b_{\mathrm{adv.},ij} = \frac{1}{2a^b_0} \sum_n \left(a^{n\cap b} - a^{n'\cap b}\right)\left(Q^n_{ij} + Q^{n'}_{ij}\right). \tag{4}$$

We further compute a corotational term $\Delta Q^b_{\mathrm{corot.},ij}$ following Ref. [5], which we find to be negligible as well (Figure 8A).

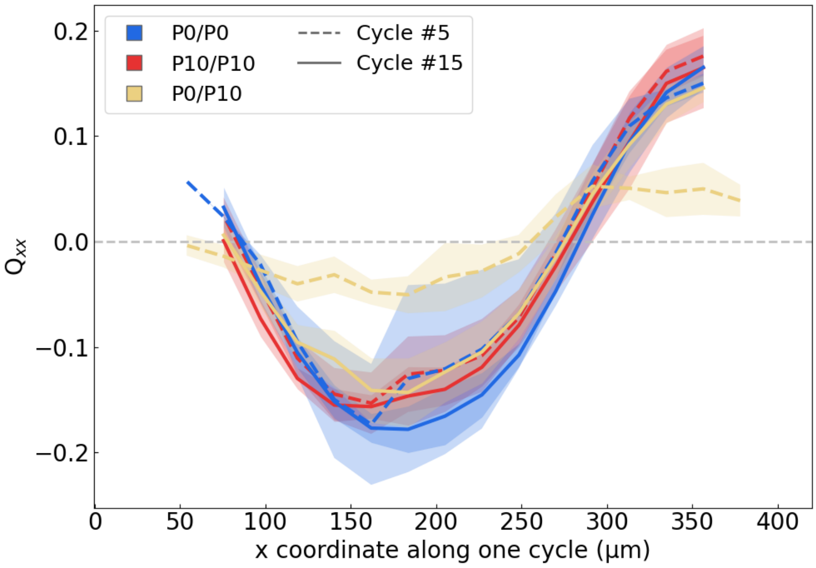

Figure 7: Examples of the Q_{xx} component of droplet shape plotted as a function of the x position in the channel, and averaged over time and across the channel width y. An increase of amplitude between position #5 (dashed lines) and position #15 (solid lines) can be observed for heterogeneous emulsions (P0/P10) (yellow curve), but not for the homogeneous emulsions P10 (red curves) and P0 (blue curves). Shaded areas represent the standard deviation.

In a next step, we average these box-based quantities, Q_{xx}, $\tilde{V}_{xx}$, $\Delta Q^b_{\mathrm{adv.},ij}$, and $\Delta Q^b_{\mathrm{corot.},ij}$, among all boxes with the same x coordinate. For this averaging, we first average over time, where we select only those boxes that are fully covered at least 95% of all time points. This yields a grid of boxes with the time-averaged quantities. At the end, we average over y in order to obtain averages for each column, i.e. quantities that only depend on x. Finally, for each x column, we compute the reversible fraction as:

$$f_r(x) = \frac{\left\langle Q^b_{xx}(t+\delta t) - Q^b_{xx}(t) + \Delta Q^b_{\mathrm{adv.},xx} + \Delta Q^b_{\mathrm{corot.},xx} \right\rangle_{t,y}}{\left\langle \tilde{V}_{xx} \right\rangle_{t,y}}. \tag{5}$$

Here δt is the length of the time interval between two movie frames and $b = (x, y)$ denotes the box position.

8.3 Quantification of droplet shape variation

To quantify how droplet shape $\boldsymbol{Q}$ changes across a cycle, we fit the measured x-dependent $Q_{xx}(x)$ to a polynomial of 5th degree, and then determine its minimum Q^{min}_{xx} and maximum Q^{max}_{xx}. We then define the amplitude for that cycle $\Delta Q_{xx} := Q^{\mathrm{max}}_{xx} - Q^{\mathrm{min}}_{xx}$. This is shown in Figure 9 (left) for data from a P0/P10 emulsion in a wavy channel. Indeed, the amplitudes in Q_{xx} increase with cycle.

8.4 Fitting of Q_* from experimental f_r curves

In the main text Figure 4D, we plot the 50% droplet anisotropy, Q_*, for given experimental reversible fraction curves $f_r(Q_{\mathrm{proj}})$. These curves are given by pairs of (Q_{proj}, f_r) values, each corresponding to one of the 96 pixel-wide columns described before. To obtain Q_* from these data, we consider values of f_r lying between 0 and 1.2 with their associated Q_{proj} and sort them. We then construct a set of consecutive data points that include the up to two lowest (respectively highest) f_r values larger (respectively smaller) than 0.5. We require to have at least one data points below and one above $f_r = 0.5$, and ignore $f_r(Q_{\mathrm{proj}})$ curves that do not fulfill this criterion. Thus, the size of the set of selected data points varies between 2 and 4. A linear fit is performed across those data points and the Q_* value is computed as the intercept of this linear fit with $f_r = 0.5$.

8.5 Beyond a Q_{proj}-dependent f_r

While we showed that a single dependency of f_r on Q_{proj} can explain most of our experimental observations, we also saw indications of additional effects.

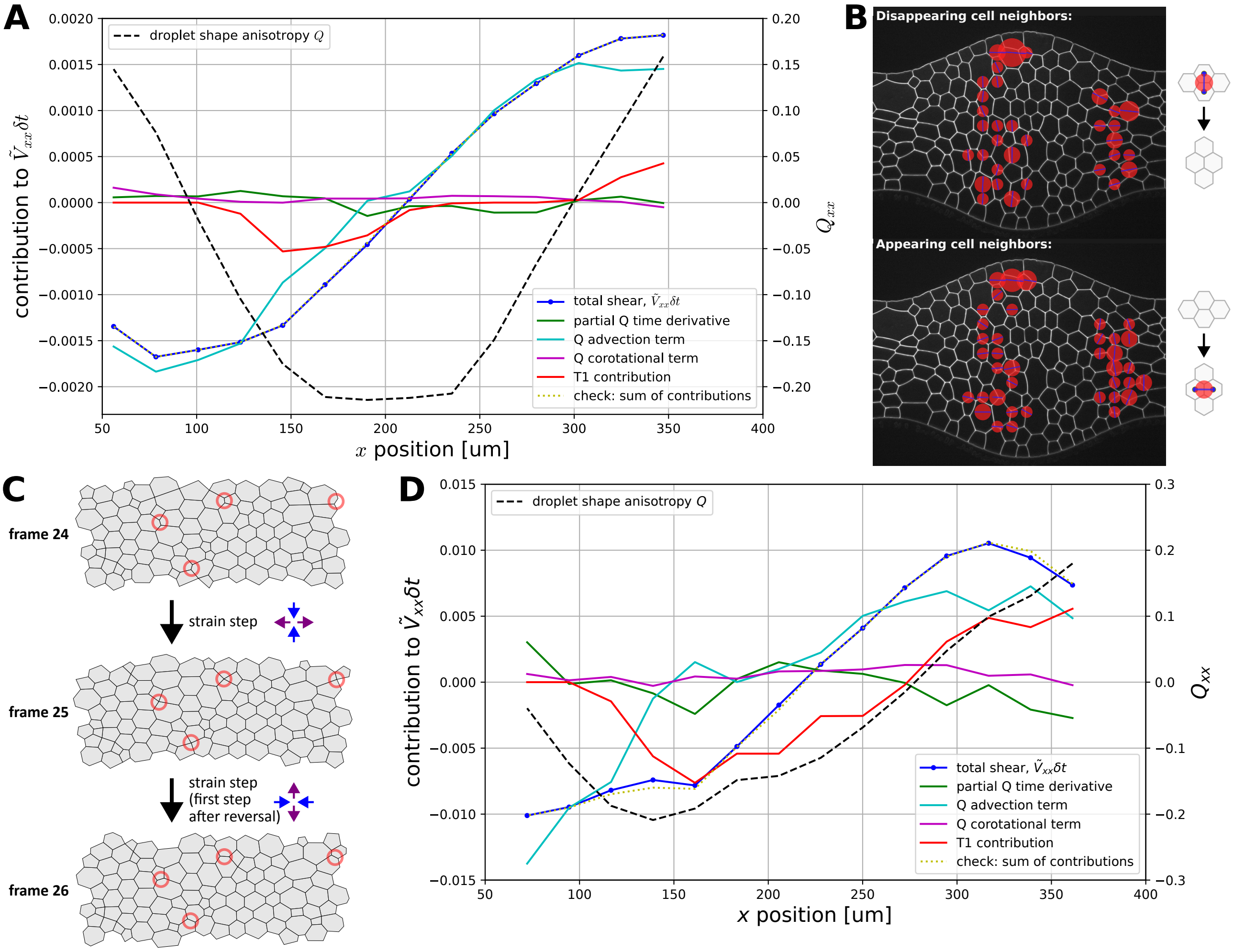

Figure 8: (A) Example of the shear rate decomposition (Eq. (1) in the main text) for a P10 emulsion across the length of a single cycle. All quantities are averaged over time and y. The overall local shear between two subsequent frames, $\tilde{V}_{xx}\delta t$, is shown as blue solid curve. We decompose this into four contributions: shear due to local change in Q_{xx} (partial time derivative, green solid curve), the advective contribution $\Delta Q^b_{\text{adv.},xx}$ (turquoise solid curve), a corotational contribution $\Delta Q^b_{\text{corot.},xx}$ (magenta solid curve), and the contribution due to T1 transitions (red solid curve). The yellow dotted curve is the direct sum of these four contributions; it overlaps with the overall shear curve as a sanity check. The black dashed line shows the droplet shape Q_{xx}. (B) As another sanity check, for the same experiment as in A, we detect individual T1 transitions by either a disappearing cell-cell neighborship (top) or an appearing cell-cell neighborship (bottom). The circle area indicates the number of T1 transitions in that region, and the blue bar indicates the average orientation of the cell-cell connection (scaled such that the bar length equals the circle diameter if all events are aligned). These data are consistent with the red curve in panel A. (C) In our simulations at shear reversals, we find that $f_r > 1$. As illustration, we show vertex model simulation snapshots for 100 cells, a strain amplitude of 0.5, and a T1 cutoff of 0.3. We observe a strong $f_r > 1$ signature in these simulations. This is consistent with T1 transitions observed in these snapshots: With the shear reversal occurring at frame 25, the red circles indicate three places where a T1 fusion occurs from frame 24 to frame 25, which continues by a T1 resolution from frame 25 to frame 26. Notably, the T1 resolution in frames 25-26 does *not* revert the fusion, but continues in the way it has started, thus leading to a T1 transition extending along the horizontal in these snapshots. Meanwhile, while overall the tissue is stretched along the vertical in frames 25-26. As a consequence of the transversal T1 transitions, droplet shape stretches along the vertical more than the tissue overall shear, implying $f_r > 1$. (D) Shear rate decomposition like in panel A, but for a P0 emulsion. Note that around $x = 250\,\mu\text{m}$, T1 transitions occur opposite to the local shear direction. We interpret this as a consequence of non-local interactions between adjacent regions of the emulsions.

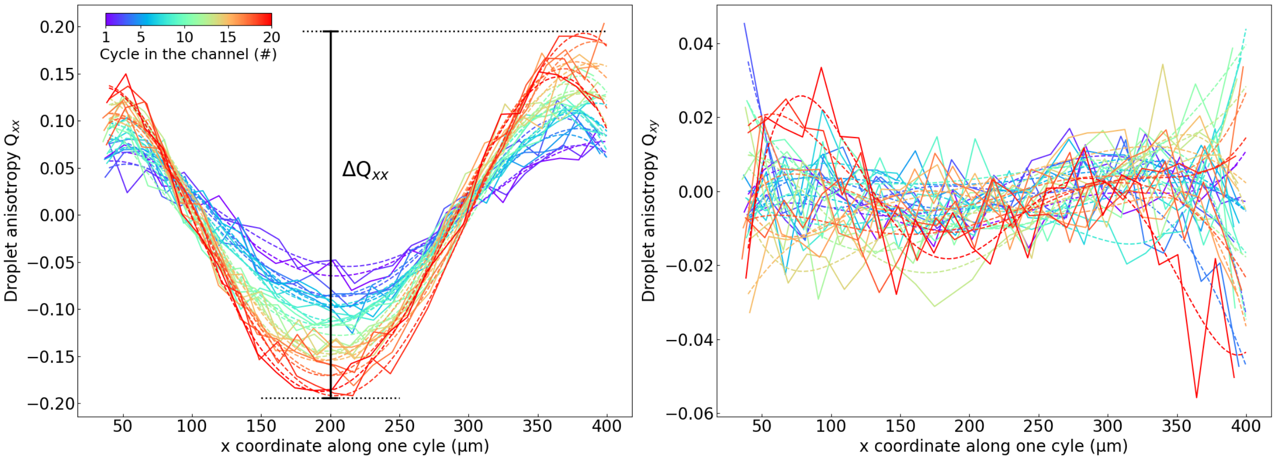

Figure 9: (Left) Spatial variation of the Q_{xx} component of droplet shape, averaged over the channel width for each cycle (color) for the case of a P0/P10 emulsion. Dashed lines represent the fit of the curves by a degree-5 polynomial. (Right) Quantification of the Q_{xy} component of droplet shape in the same experimental condition.

For instance, we saw that both in experiments and simulations, f_r became larger than one for the smallest, negative values of Q_{proj}. In the simulations, we saw that this was related to the switching of the shear direction: In Figure 8C, we show three frames for a vertex model simulation around the switch of the shear direction. We find that in the transition leading up to the switch, frames 24-25, a few edges fuse into manyfold vertices (T1 fusions). Meanwhile, after the switch of shear directions, in frames 25-26, these manyfold vertices revolve, but instead of going back to how they were before, they continue. In other words, the T1 transitions continue to expand horizontally, while tissue overall expands vertically (contracting horizontally). As a consequence, droplet shapes expand vertically, but due to the T1 orientation more than the tissue overall, i.e. $f_r > 1$ for the transition between frames 25-26. This is some form of short-term memory.

In the experiment, the reason for $f_r > 1$ might be the same. Alternatively or additionally, $f_r > 1$ in the experiments may also be created by elastic non-local effects: Whenever there is some local deformation of a given droplet in the emulsion, this will also affect the shapes of close-by droplets. We can observe this for example during T1 transitions, where we see a quasi-instantaneous deformation of surrounding droplets. This deformation will slightly change the shapes of the surrounding droplets, and may trigger another T1 transition. The transitions that we observe at shear reversal in the experiments may in part due to such non-local effects.

We also see indications for such non-local effects in our shear decomposition for some emulsions. In Figure 8D, we show the shear decomposition for a P0 emulsion. Notably, around $x = 250\,\mu\mathrm{m}$, the emulsion is sheared extending along the horizontal (positive values of blue curve), but T1 transitions occur along the vertical (negative values of red curve). This would be impossible if emulsion behavior would be only controlled locally, since shearing horizontally would induce T1 transitions horizontally. Hence, this suggests a role of non-local effects, for instance by the nearby region between $x = 100 \dots 200\,\mu\mathrm{m}$ undergoing vertically extending T1 transitions, whose elastic propagators may push a sufficient number of droplets in the $x = 250\,\mu\mathrm{m}$-region to also undergo vertically extending T1 transitions.

9 Quantification of the flow velocity for the prediction of $Q_{xx}(x)$

In order to predict $Q_{xx}(x)$ for the reversible fraction function, we need the velocity v_x, at least up to a prefactor (see Methods in main text). Because the emulsions are essentially incompressible, the total flux of emulsion should be the same at every position x when integrated across the width of the channel. Using the approximation that v_x only depends on x, but not on y, and denoting the channel width at some position x by $h(x)$, we thus have:

$$\mathrm{v}_x(x) = \frac{h(0)\mathrm{v}_x(0)}{h(x)}. \tag{6}$$

Yet, we found that this yields a slightly imprecise prediction for the shear rate $\tilde{V}_{xx}$ averaged over time and y as described in the previous section. This is due to the fact that the triangulations do not cover the entire channel; there is a region close to the boundary not covered by triangles since the triangles only reach up until the barycenters of the boundary droplets. This boundary region is captured by (6), but it is not captured by $\tilde{V}_{xx}$. Since what is most relevant for the prediction of the Q_{xx} is what happens to the triangular region, we thus

decided to base our Q_{xx} prediction on $\tilde{V}_{xx}$.

To compute the prediction described in the Methods part of the main text, we need a smooth function, while experimentally we measured $\tilde{V}_{xx}$ only averaged over the discrete x columns. We thus fitted the velocity and velocity gradient data to a generic Fourier expansion:

$$\mathrm{v}_x^{\mathrm{fit}}(x) = a_0 + a_1 \cos\left(\frac{2\pi(x - x_1)}{\lambda}\right) + a_2 \cos\left(\frac{4\pi(x - x_2)}{\lambda}\right) \tag{7}$$

Here, $a_{0/1/2}$ are fit parameters corresponding to Fourier mode amplitudes and $x_{1/2}$ are fit parameters corresponding to phase shifts. The constant $\lambda = 420\,\mu\mathrm{m}$ is the wave length of the cycles. In practise, we first fit $\mathrm{dv}_x^{\mathrm{fit}}/\mathrm{d}x$ to the measured $\tilde{V}_{xx}$ over x data (note that $\tilde{V}_{xx} = \partial_x v_x$ because of incompressibility). This allows us to fix $a_{1/2}$ and $x_{1/2}$. Afterwards, we obtain a_0 by fitting $\mathrm{v}_x^{\mathrm{fit}}$ to the column-wise averaged velocities of the droplet centers, keeping the previously determined $a_{1/2}$ and $x_{1/2}$ fixed and focusing on the triangulated region.

10 Packing fraction evolution

The packing fraction of the emulsions is estimated from 2D Voronoi images as shown in Figure 10A-B. Since the equatorial plane used for this quantification is chosen manually, we estimate the error induced by this choice by measuring the local packing fractions in two slices that are located 1 µm above and 1 µm below the chosen equatorial plane. Doing this for two distinct conditions, namely P0/P10 and P6/P10 emulsions (data not shown), yields differences in the average packing fractions measured above and below the identified equatorial planes that are significantly smaller than the standard deviation of the values measured within each plane.

In the case of heterogeneous emulsions (P0/P10) submitted to oscillatory perturbations, the progressive increase of deformation is accompanied by a progressive increase of local packing fraction (quantified for each droplet as the ratio between droplet area and area of its associated Voronoi cell) as observed in Figure 10 (C, middle row) by a continuous shift of the packing fraction distributions towards higher values as the emulsions progress in the channel. Conversely, unperturbed P0/P10 emulsions (Figure 10, C, top row) and homogeneous P10 emulsions (Figure 10, C, bottom row) do not display the same range of evolution.
Alternatively, one can also examine specific emulsions in static acquisitions. As shown in Figure 10 (D), no matter the initial value, heterogeneous P0/P10 emulsions show a significant increase of packing fraction, all tending towards $\phi \sim 1$. In contrast, unperturbed heterogeneous P0/P10 emulsions and homogeneous P10 emulsions exhibit no increase of packing fraction even when the initial packing fraction at the entry of the channel compares to that of their heterogeneous counterpart.

11 Estimation of interface tension ratio from contact angles at triple junctions

Any internal angle between interfaces of an emulsion with heterotypic interface tensions is given by [6]:

$$\cos\theta_1 = \frac{\lambda_1^2 - \lambda_2^2 - \lambda_3^2}{2\lambda_2\lambda_3}, \tag{8}$$

where θ_1 and $\lambda_{1/2/3}$ are defined as indicated in Figure 11 left. In the special case where $\lambda_1 = \lambda_2 = \lambda_3$, the right-hand side evaluates to $-1/2$, which corresponds to the Plateau-rule angle of $\theta_1 = 120°$.

In our heterogeneous P0/10 emulsions, there are four possibilities for the kinds of droplets that meet in a triplet junction: (i) three P10 droplets, (ii) two P10 and one P0 droplets, (iii) one P10 and two P0 droplets, and (iv) three P0 droplets. For cases (i), (iii), and (iv), we would expect equal interface tensions areound the triple junction, and thus Plateau angles of 120°. The only possibility where we would expect heterotypic interface tensions around the triple junction is (ii), two P10 and one P0 droplets (Figure 11 right). In this case, we expect a 10-bp adhesion between the two P10 droplets, which leads to an effective interface tension of λ_{P10}, but no adhesion between P10 and P0 droplets, corresponding to an interface tension of λ_{P0} (Figure 11 right). In this case, we obtain from (8):

$$\frac{\lambda_{\mathrm{P10}}}{\lambda_{\mathrm{P0}}} = \sqrt{2\big(1 + \cos\theta_{\mathrm{P10}}\big)} \qquad \text{and} \qquad \frac{\lambda_{\mathrm{P10}}}{\lambda_{\mathrm{P0}}} = -2\cos\theta_{\mathrm{P0}}, \tag{9}$$

where the angles θ_{P10} and θ_{P0} are defined as indicated in Figure 11 right. Given that we adhesion should reduce reduce the effective interface tension, we expect the upper limit $\lambda_{\mathrm{P10}}/\lambda_{\mathrm{P0}} \leq 1$.

From the segmented snapshots, we quantified the inter-interface angles around each inner vertex by fitting each edge with a straight lines from the junction (see Section 4 for analysis details and Figure 3I for examples).

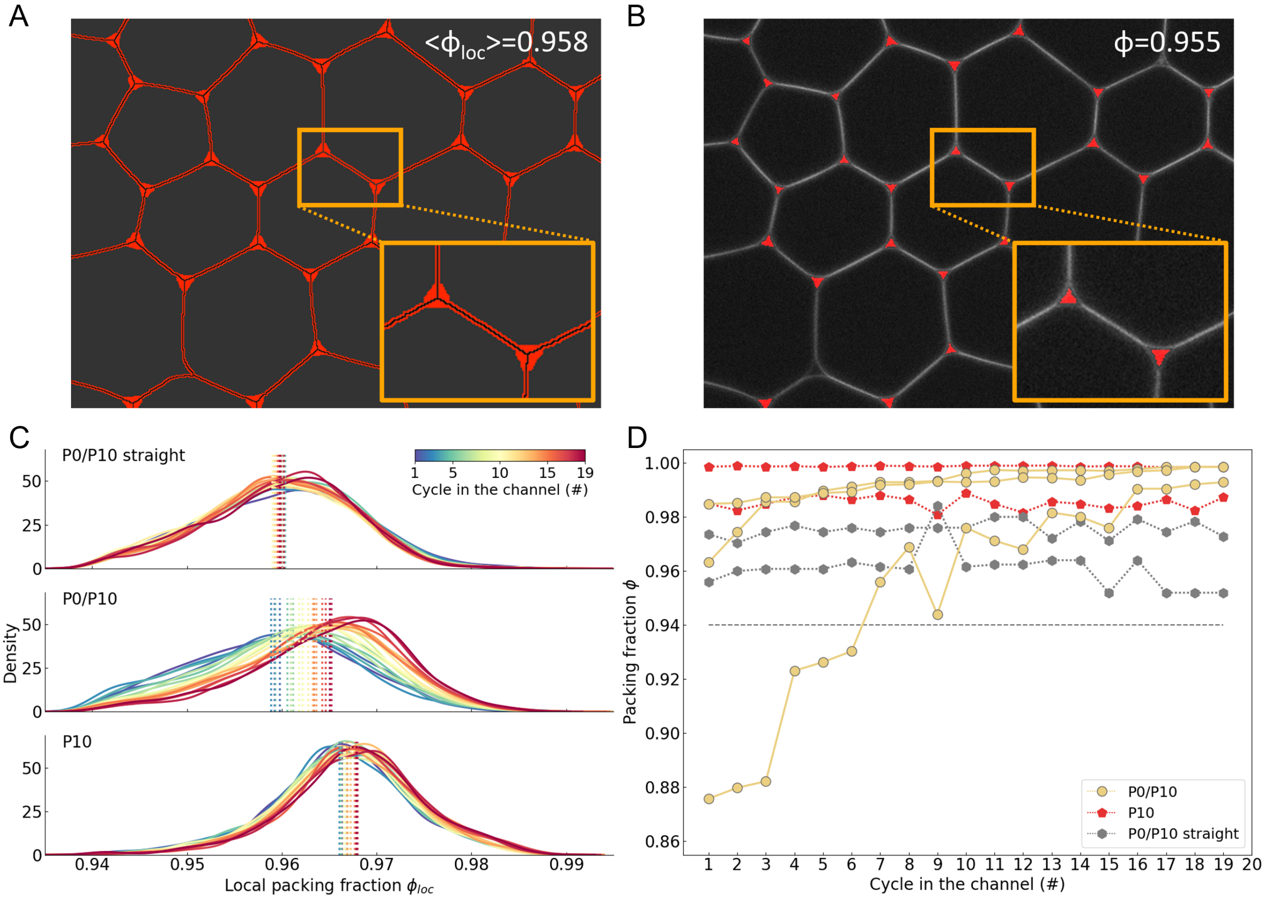

Figure 10: (A) Superimposition of the detected droplets (grey) on the corresponding surface Voronoi tessellation (red). Dividing the droplet area by the area of the corresponding Voronoi cell yields a local packing fraction ϕ_{loc}. On this image, the average local packing fraction is of about 0.958. (B) Packing fraction can also be estimated from the area of the triangles at tri-cellular junctions, which is the method we used in panel D, and to analyze the movies. With this method the packing fraction yields ∼0.955 (on the same image as (A)). (C) Distribution of the local packing fraction in static acquisitions for different experimental conditions (top to bottom: heterogeneous emulsions P0/P10 in a straight channel, heterogeneous emulsions P0/P10 in a wavy channel and homogeneous emulsions P10 in a wavy channel), for all position (purple (position #1) to red (position #19)). Periodically perturbed heterogeneous emulsions exhibit a continuous shift towards higher values of packing fraction along the oscillatory channel until matching the distribution of the homogeneous emulsions in the same channel, which remains constant across positions. In contrast, heterogeneous emulsions in the straight channels exhibits a similar initial distribution as the same emulsions in the wavy channel but no evolution as moving further in the channel. Dashed lines represent the average packing fraction corresponding to each distributions. (D) Examples of the evolution of ϕ across channels measured in static acquisitions. Noticeably, all curves from the P0/P10 emulsions subject to oscillatory shear (P0/P10, yellow circles) exhibit an increase of packing fraction towards a similar high value, independent of the initial packing fraction. In contrast, for unperturbed heterogeneous emulsions (P0/P10 in straight channel, grey hexagons) or homogeneous ones (P10, red pentagons), even with a similar starting point as the P0/P10 perturbed emulsion, the curves remains stable across the whole channel. The grey dashed line at $\phi = 0.94$ represents the cutoff used to filter the data in static acquisitions.

We measure these angles automatically for more than 10^5 tri-cellular junctions in P0/P10 and P10 emulsions in undulated channels, as well as P0/P10 emulsions in straight channels (respectively: 196155,142833 and 220332 angles were measured), all positions of the channel combined. This analysis yields distributions with the following averages and standard deviations: P0/P10 emulsion in undulated channel: $120 \pm 8.85°$, P10 emulsion in undulated channel: $120 \pm 10.48°$ and P0/P10 emulsion in straight channel: $119.98 \pm 8.58°$ (Fig. 12).

For now, our angle analysis code does not take into account the droplet identity. In other words, the measured values of the standard deviation includes all angles in all three-droplet junctions independent of droplet identity. Thus, to turn the obtained results into an upper bound estimate for the interface tension ratio, we first derive an expression for the angle standard deviation σ_θ: The average measured angle is, as

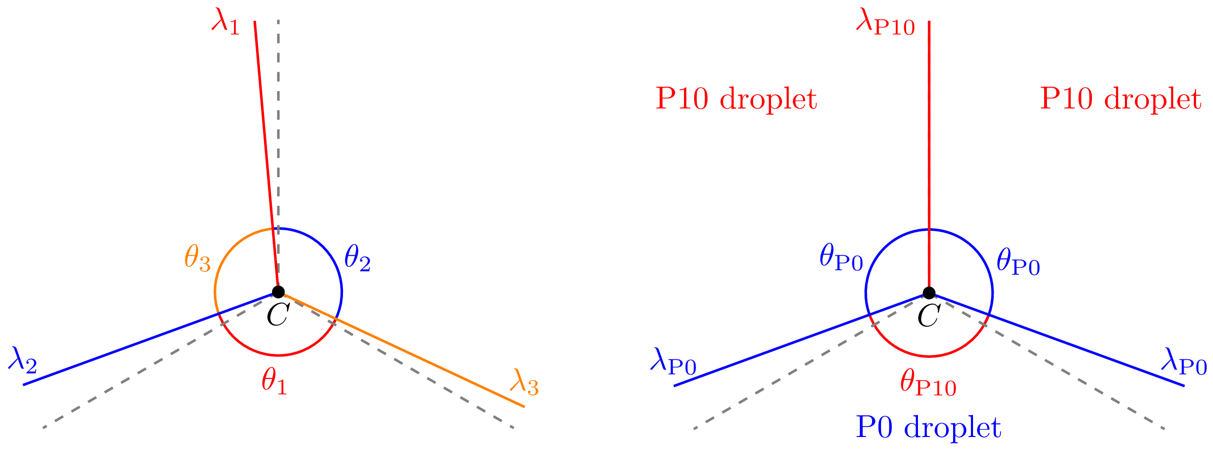

Figure 11: Mechanical equilibrium at the tricellular junction. The dashed gray lines corresponds to the 120°-Plateau rule. (Left) Generic case with three distinct line tensions λ_1, λ_2 and λ_3. (Right) Situation of two P10 droplets and one P0 droplet.

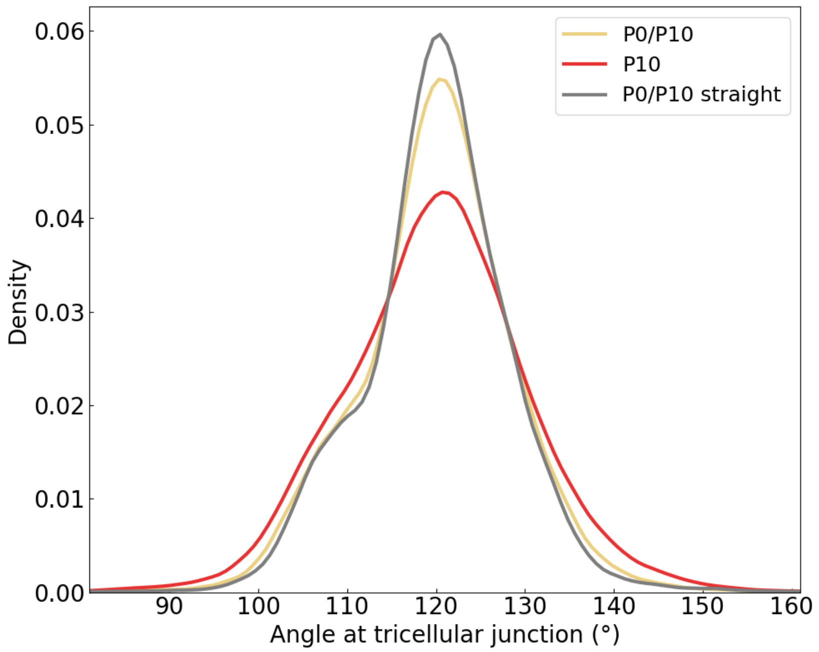

Figure 12: Distributions of measured angles around triple junctions in P0/P10 and P10 emulsions in undulated channels, as well as P0/P10 emulsions in straight channels.

expected $120°$. To obtain the expected variance assuming a heterogeneous interface tension, we note that only in the droplet configuration "two P0 and one P10", we expect to see angles other than $120°$. In a well mixed sample, this three-droplet configuration appears with a probability of $3/8$, and it contributes three angles to the variance: two angles are $\theta_{\mathrm{P0}} = 120° - \Delta\theta_0$, with some constant deviation $\Delta\theta_0$, and the third angle is $\theta_{\mathrm{P10}} = 120° + 2\Delta\theta_0$ (compare Fig. 11 right). Combined with the fact that our measured standard deviation also includes measurement noise, we obtain the following lower bound: $\sigma_\theta \geq \Delta\theta_0\sqrt{3}/2$. For the P0/P10 emulsion in the undulated channel, we got $\sigma_\theta = 8.85°$, and using (9), we obtain the lower limit for the line tension ratio $\lambda_{\mathrm{P10}}/\lambda_{\mathrm{P0}} = -2\cos\left(120° - \Delta\theta_0\right) \geq -2\cos\left(120° - 2\sigma_\theta/\sqrt{3}\right) \approx 0.68$.

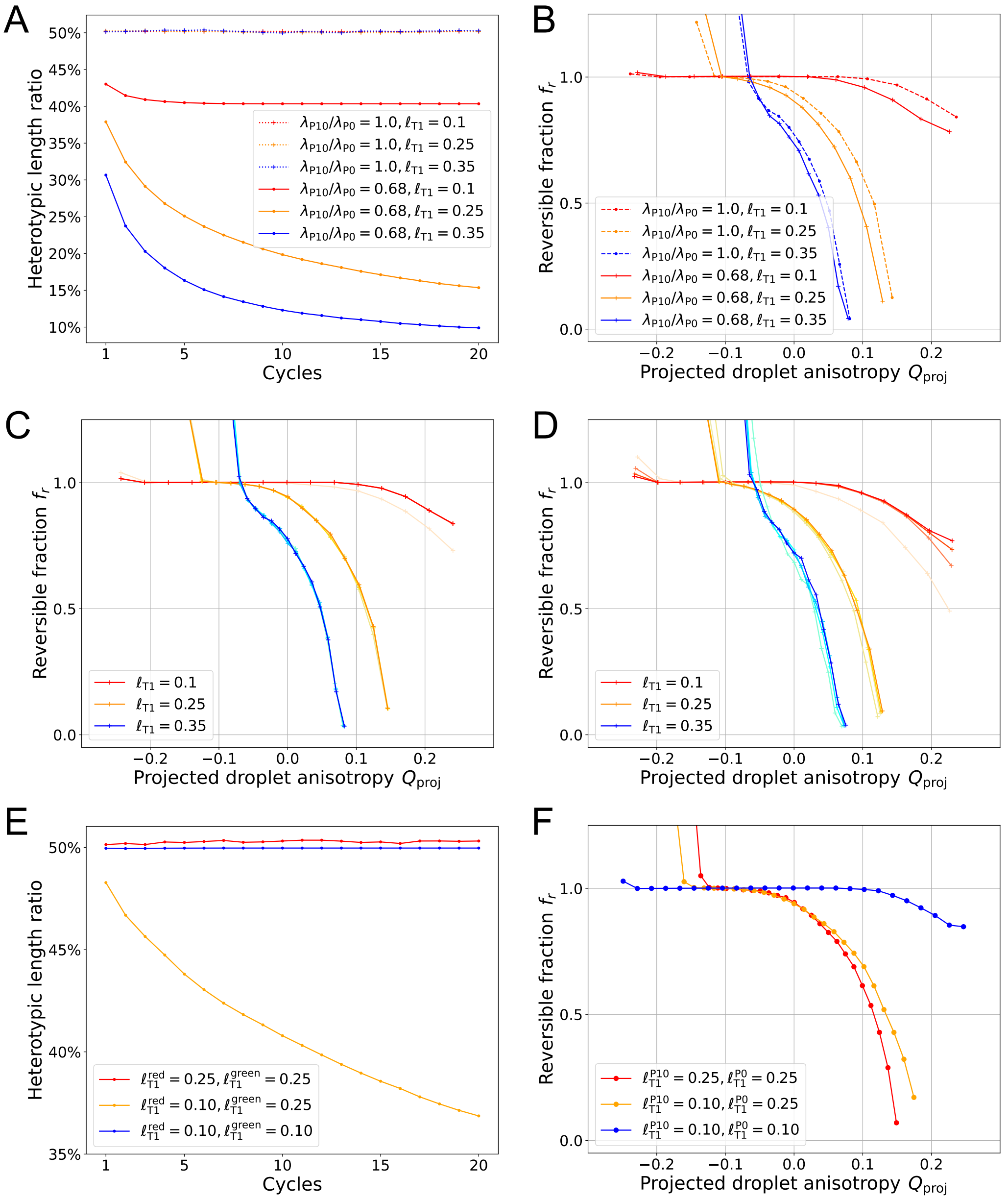

Figure 13: Vertex model simulation results to test whether adhesion directly affects heterogeneous interface tensions or the T1 cutoff. (A) Evolution of the heterotypic length ratio (total length of heterotypic interfaces didived by total length of all interfaces) as a function of the number of shear cycles. The heterogeneous line tension vertex model demixes (solid lines, $\lambda_{P10}/\lambda_{P0} = 0.68$), and demixing speed increases with the value of the T1 cutoff, ℓ_{T1}, as more T1 transitions are allowed. (B) Reversible fraction curves $f_r(Q_{\mathrm{proj}})$ for the simulations in panel A, each averaged over the last 5 out of 20 cycles. The heterogeneous vertex model with $\lambda_{P10}/\lambda_{P0} = 0.68$ (solid lines) yields slightly more easily than its homogeneous counterpart (dashed lines). Moreover, an increase in the T1 cutoff ℓ_{T1} generally leads to an easier yielding. (C,D) Drift in the yielding behavior: reversible fraction curves $f_r(Q_{\mathrm{proj}})$ for different T1 cutoffs (curve color), averaged over cycles: 1-5 (curve of the lightest shade for a given color), 6-10, 11-15, and 16-20 (curve of the darkest shade for a given color). We show both homogeneous (C) and heterogeneous interface tensions with $\lambda_{P10}/\lambda_{P0} = 0.68$ (D). For both homogeneous and heterogeneous interface tensions, and for small T1 cutoff ($\ell_{T1} = 0.1$, red curves), we observe a transient drift in the yielding behavior, roughly over the first 5 cycles (from light to dark shades), where yielding becomes harder over time. This is likely related to some transient relaxation of the initial structural disorder [4]. Such a drift is not observed for higher ℓ_{T1}, possibly because such a structural relaxation may occur much faster in these cases. (E) Evolution of the heterotypic length ratio as a function of the number of shear cycles. We observe that a vertex model with heterogeneous T1 cutoffs demixes (orange curve), contrary to its homogeneous counterparts (blue and red curves). (F) Associated reversible fraction curves f_r, averaged over the last 5 out of 20 cycles. We first observe that the vertex models with homogeneous T1 cutoffs yield easier for larger ℓ_{T1} (consistent with panel B). Second, we see that the yielding behavior for heterogeneous T1 cutoffs (orange curve) is intermediate between the homogeneous cases (blue and red curves), but closer to the one with the larger T1 cutoff. The values for the T1 cutoffs in these simulations were chosen to roughly cover the range that corresponds to our experimental data (see Figure 4C,D in the main text).15

12 Estimation of the parameter α

In order to estimate the parameter α in Figure 4D in the main text, we used the `minimize` function from the `scipy.optimize` python library to perform a least-squares fit comparing the packing fraction values ϕ between experiments and vertex model simulations.

To evaluate the square deviation function for a given choice of α, we compute for each experimental data point $(\phi^{\text{exp}}, Q_*^{\text{exp}})$ an interpolated theoretical value ϕ^{interp}. To this end, we first use the vertex model simulation data, which consists of data points $(\ell_{\text{T1}}^{\text{sim}}, Q_*^{\text{sim}})$ to linearly interpolate the $\ell_{\text{T1}}^{\text{interp}}$ value corresponding to Q_*^{exp}. We do this using the `interp` function from the `numpy` python library. Finally, we compute the packing fraction ϕ^{interp} from the interpolated T1 cutoff using $\phi^{\text{interp}} = 1 - (\ell_{\text{T1}}^{\text{exp}}/\alpha)^2$. The function to be minimized is then given by $(\phi^{\text{exp}} - \phi^{\text{interp}})^2$ summed over all experimental data points whose Q_*^{exp} lies between the minimal and maximal Q_*^{sim}.

This fitting procedure yields a value of $\alpha \approx 4.4$.

13 A gradient in packing fraction suggests pumping

13.1 Hypothesis

The continuous phase is incompressible. Hence, under stationary conditions, the total volume flux of the continuous phase should be independent of the position in the channel:

$$h(x)\phi_c(x)\mathrm{v}_c(x) = \text{const.} \tag{10}$$

Here, h is the channel width, $\phi_c = 1 - \phi$ is the packing fraction of the continuous phase, and v_c is the velocity of the continuous phase. Using periodicity of the channel width, $h(x) = h(x + n\lambda)$ for any $n \in \mathbb{Z}$ and λ being the wavelength, we obtain:

$$\phi_c(x)\mathrm{v}_c(x) = \phi_c(x + n\lambda)\mathrm{v}_c(x + n\lambda). \tag{11}$$

Hence, a decrease in ϕ_c across cycles, as we observe it for instance in P0/P10 emulsions, corresponds to an increase in the magnitude of v_c across cycles. Yet, note that in principle, the sign of v_c could in principle be either positive or negative. Yet, preliminary observations using tracer particles suggest that it is positive.

Meanwhile, the same equation as (11) holds also for the discontinuous/droplet phase:

$$\phi_d(x)\mathrm{v}_d(x) = \phi_d(x + n\lambda)\mathrm{v}_d(x + n\lambda). \tag{12}$$

Here, $\phi_d \equiv \phi \simeq 1$, and thus the velocity v_d of the droplet phase is essentially constant among subsequent cycles.

Taken together, a decrease of $\phi_c \ll 1$ corresponds to a change in the velocity of the continuous phase across cycles, while the velocity of the droplet phase is essentially constant. Since there is friction between both phases, such a difference in velocity can only be maintained if there is an effective mechanism that "pumps" both phases with respect to each other, i.e. that drives a non-vanishing relative flow.

13.2 Flow velocity measurements

In order to evidence this potential pumping mechanism, we performed preliminary experiments aiming to analyze the flows of both the droplets and the continuous aqueous phase. To do so, we studied these flows in a heterogeneous P0/P10 emulsion, in which the continuous phase is supplemented with 0.1% v/v of fluorescent particles (FluoSpheres carboxylate, 0.1μm red 580/605, Invitrogen). The emulsion was then injected in the channel and movies were acquired in the red channel at 10 Hz. Note that here the particles were quite big compared to the interdroplet space in a compressed emulsion and therefore acquisitions were performed slightly above the focal plane from which they would be otherwise excluded. In the following we describe the procedure developed in order to analyze those movies and present some preliminary results.

The flow of the continuous phase is analyzed through imaging of the tracers (see Figure 14A and movie S2). We selected a small window of $560 \times 315 px^2$ in the center of the image and applied a threshold in order to isolate the tracers from the background. Since some tracers remained stuck to the glass wall of the channel, we got rid of them but summing all the slices of the movie and by making a mask in which we only kept the dots of highest intensity corresponding to the stuck particles. Subtracting this mask from the original thresholded movie, we obtained a clear binary movie of our tracers that we then tracked using the LAP tracker from Trackmate. We discarded tracks shorter than 1/3 of the track of maximal duration (in terms of timepoints) which represents 3965 tracks. The 246 remaining tracks were then used to compute the speed of the particles in the continuous phase.

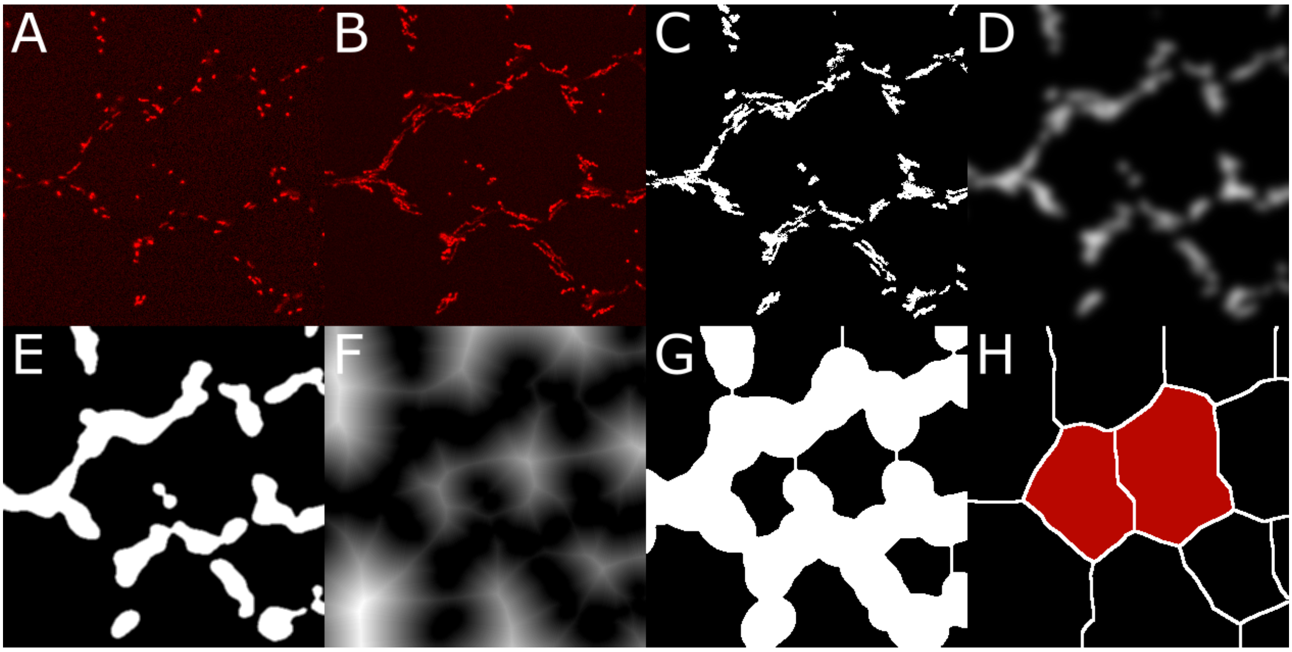

Figure 14: Droplet identification from movies with fluorescent tracers. (A) We define a small window in the middle of the channel on the raw image. (B) Images of the movie are projected five by five to recreate a movie in which each image is the projection of the max intensity of five consecutive images from the original movie. (C) Projections are manually segmented to get a binary image of the tracers in which stuck immobile particles are filtered out. (D) We apply a Gaussian blue filter of 5px to image (C), and threshold this image to obtain (E), from which we perform a distance map of the pixels compared to the identified contours (F). (G) The distance map is thresholded and we apply a watershed on the obtained binary mask. (H) Last, we perform a surface Voronoi tessellation of the mask to get the final contour of the droplets.

Since the droplets could not be imaged together with the tracers, they were only identified from the negative of the particles in the fluid phase. Although this doesn't give the precise contours of the droplets, it allows one to analyze their position and hence their flow. To do so, we first used the "grouped z projection" function from Fiji to obtain a movie in which each image is the projection of the max intensity of five consecutive images (dividing the number of images in the movie by 5, thanks to the relatively low velocity of the droplets this did not impaired the detection of droplets' movement). This process allowed us to get some slightly more continuous contours for the droplets (see Figure 14B). We then applied a manual threshold on this image, removed immobile tracers as described above, and created a binary image of the contours (see Figure 14C). We next applied a Gaussian blur of 5px and once again manually thresholded the image (see Figure 14D-E). After inversion of the image, we created a distance map of our droplets (see Figure 14F). After thresholding, we used the Fiji watershed function to separate groups of droplets identified as one result after this step (see Figure 14G). Finally, we performed a surface Voronoi tessellation which gave us the final contour of the droplets visible in Figure 14H.

In the following, we considered only the droplets that were not touching the borders of the image and that were continuously detected over time (red droplets in Figure 14H). Droplets were tracked using the LAP tracker from Trackmate yielding instantaneous displacements along x and y. As each image was obtained from the projection of 5 consecutive images, in a first approximation we divided by 5 these displacements before computing the speed. From this analysis, we find that the velocity along the x-axis (Figure 15-Left) is two to three times higher for the tracers in the continuous phase than for the droplets, while the velocity along the y-axis is in both cases oscillating around zero (Figure 15-Right). Interestingly, we can observe some spikes of velocity of the droplets both along the x and the y directions that may correspond to T1 events (e.g. around 5, 10, 25 and 30 in Figure 15).

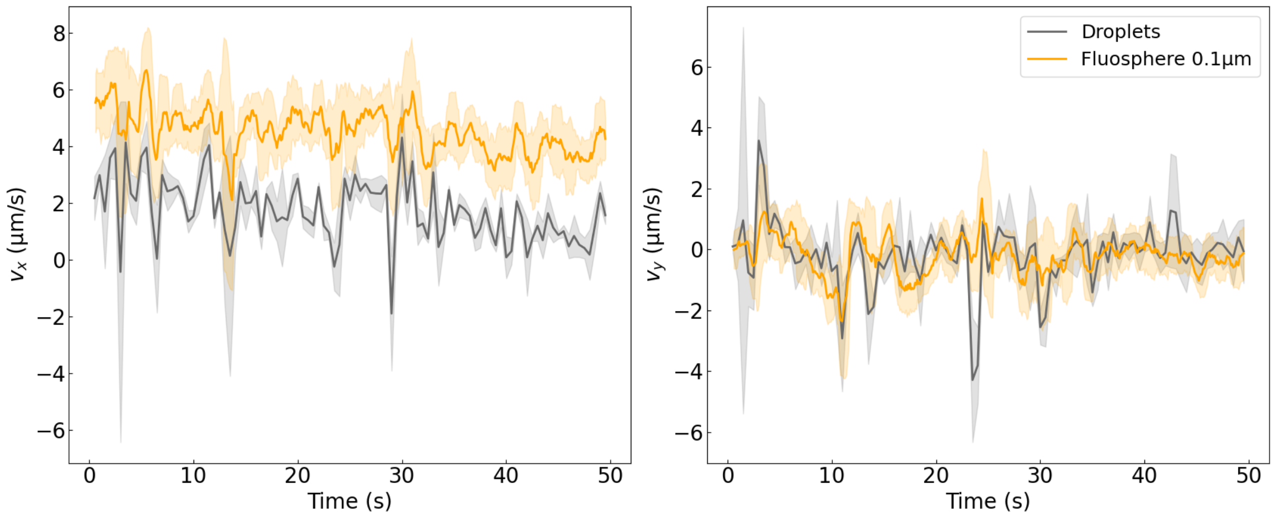

Figure 15: Measured velocity of the droplets and the continuous phase in a P0P10 emulsion. (Left) and (Right) show the velocity along the x and y directions respectively for the tracers (yellow curves) and the identified droplets (grey curves). In the case of the droplets, each point is averaged over all droplets of 5 consecutive timepoints, errorbars represent the standard deviation. For the tracers, each point is an average over all particles combined with a rolling window of 10 frames, errorbars represent the standard deviation of the average in this time-window.

14 Supplementary Videos

14.1 Movie S1: flow of an heterogeneous emulsion

Video showing a P0/P10 emulsion flowing through the fifth undulation of the wavy channel with an average measured speed of $24\mu m/s$. The packing fraction of the emulsion was evaluated at $\phi = 0.997$ using the holes measurement method. Original frame rate is twenty images per second.

14.2 Movie S2: flow of an heterogeneous emulsion with fluorescent tracers in the continuous phase

Video showing a P0/P10 emulsion flowing through the fifth undulation of the wavy channel. Only the small window analyzed in subsection 13.2 is shown. Droplets were functionalized as usual and the continuous phase was supplemented with fluorescent particles (FluoSpheres carboxylate, $0.1\mu m$ red 580/605, Invitrogen) in order to characterize the flow of the continuous phase. Acquisition was performed slightly above the focal plane. Original frame rate is ten images per second.

References

[1] Stuart Berg et al. "Ilastik: interactive machine learning for (bio) image analysis". In: *Nature methods* 16.12 (2019), pp. 1226–1232.

[2] Rémi Dreyfus et al. "Simple quantitative model for the reversible association of DNA coated colloids". In: *Physical review letters* 102.4 (2009), p. 048301.

[3] Lang Feng et al. "Specificity, flexibility and valence of DNA bonds guide emulsion architecture". In: *Soft Matter* 9.41 (2013), p. 9816. ISSN: 1744-683X. DOI: 10.1039/c3sm51586a. URL: http://xlink.rsc.org/?DOI=c3sm51586a.

[4] Alexandre Kabla and Georges Debregeas. "Quasi-Static Rheology of Foams. Part 1. Oscillating Strain". In: *Journal of Fluid Mechanics* 587 (2007), pp. 23–44. ISSN: 14697645. DOI: 10.1017/S0022112007007264.

[5] Matthias Merkel et al. "Triangles bridge the scales: Quantifying cellular contributions to tissue deformation". In: *Physical Review E* 95.3 (Mar. 2017), p. 032401. ISSN: 24700053. DOI: 10.1103/PHYSREVE.95.032401/FIGURES/17/MEDIUM. arXiv: 1607.00357. URL: https://journals.aps.org/pre/abstract/10.1103/PhysRevE.95.032401.

[6] John Shipley Rowlinson and Benjamin Widom. *Molecular theory of capillarity*. Courier Corporation, 2013.
